# Supplementary figures and images for: Metabolic clogging of mannose triggers dNTP loss and genomic instability in human cancer cells
Source: eLife. 2023 Jul 18;12:e83870. doi: 10.7554/eLife.83870 (PMC10353863; doi:10.7554/eLife.83870)

Figure 1-source data 1

full raw unedited blots ( $\beta$ -Actin)

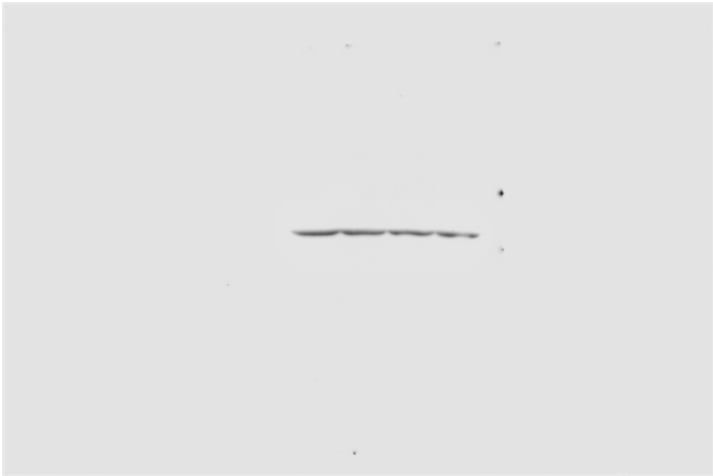

full raw unedited blots (MPI)

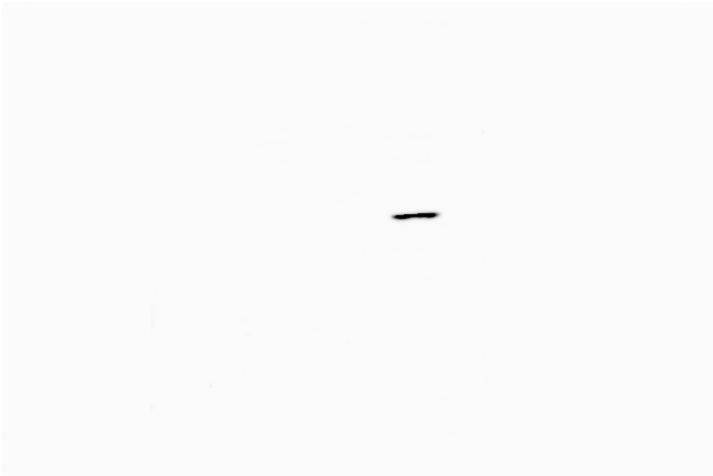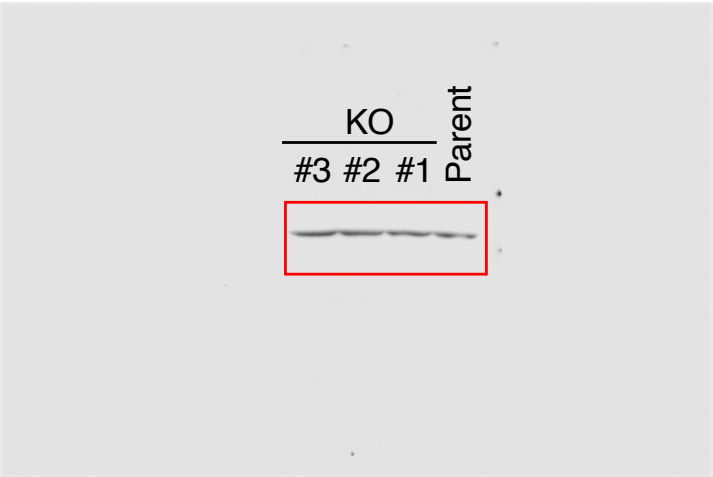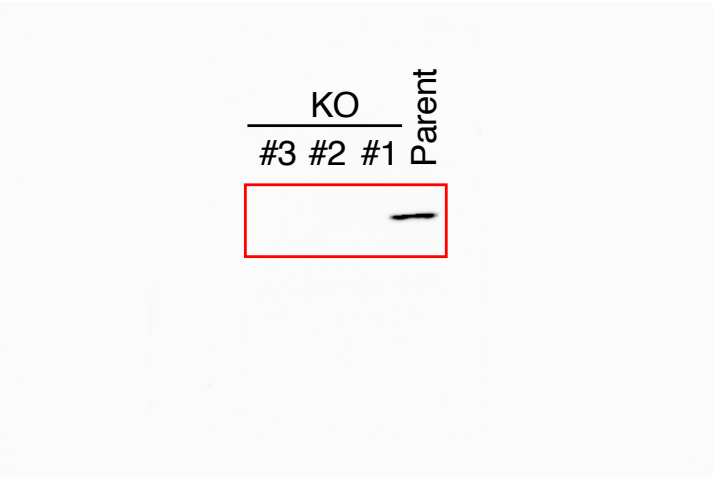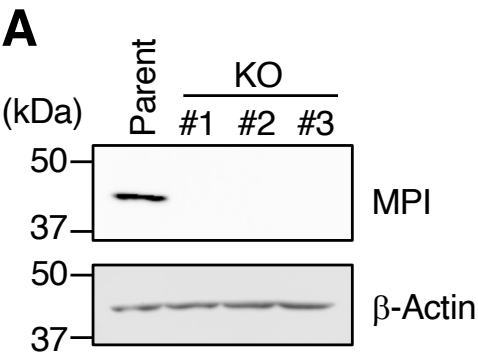

Supplement: Figure 1—source data 1. [file elife-83870-fig1-data1.zip › Figure 1-source data 1/Figure 1-source data 1.pdf]

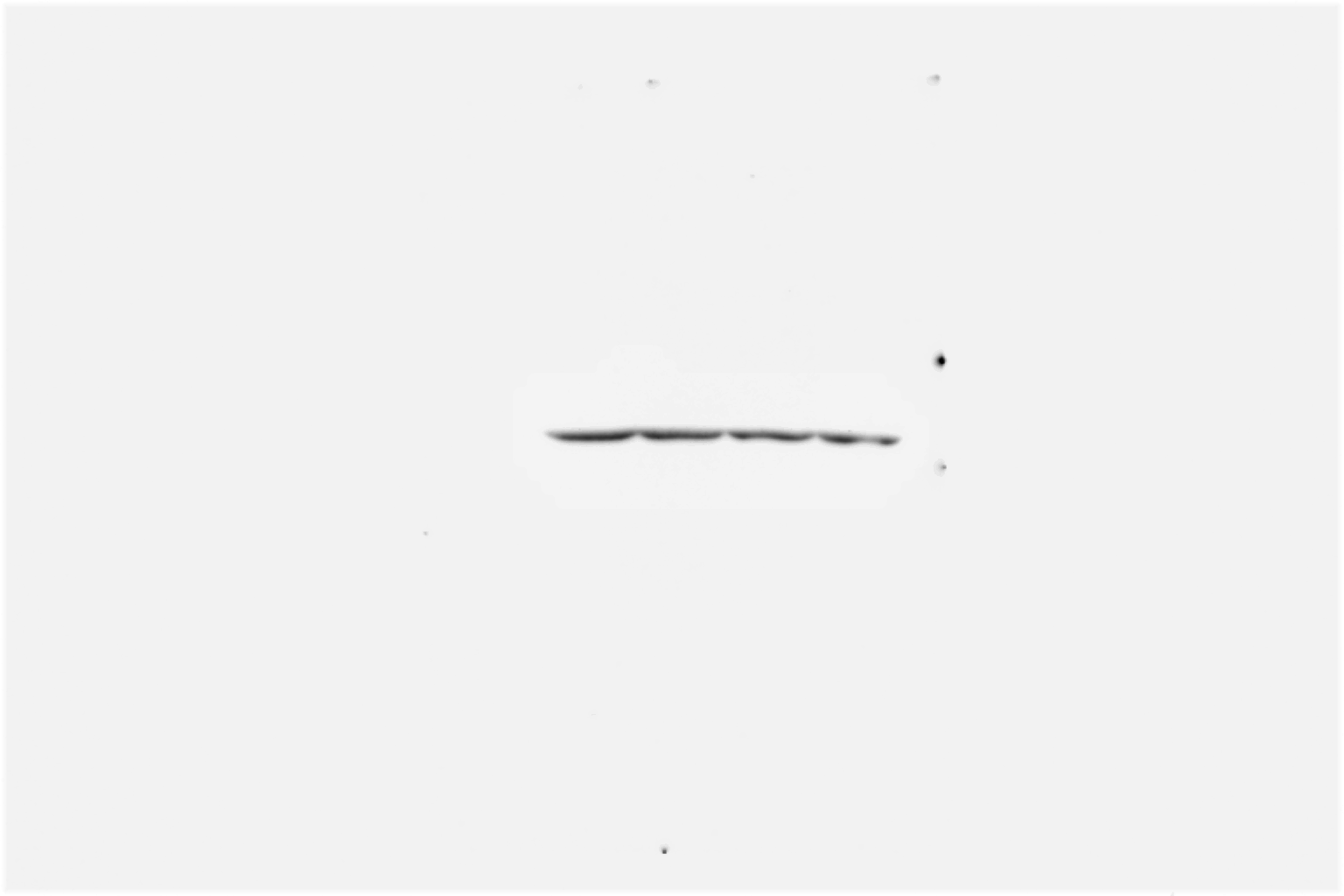

Supplement: Figure 1—source data 1. [file elife-83870-fig1-data1.zip › Figure 1-source data 1/Figure 1-source data 1 (ACTB).tif]

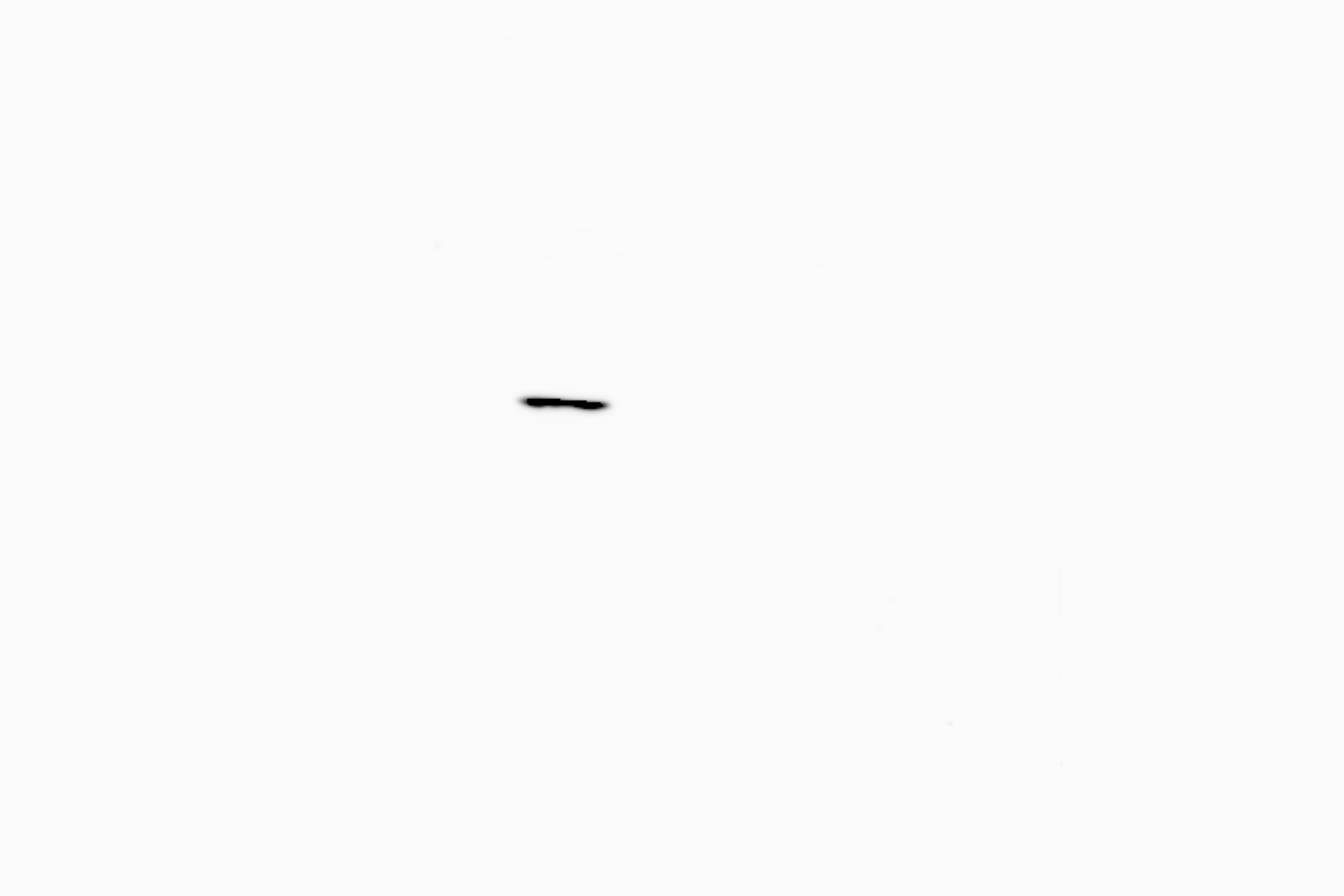

Supplement: Figure 1—source data 1. [file elife-83870-fig1-data1.zip › Figure 1-source data 1/Figure 1-source data 1 (MPI)_flipped.tif]

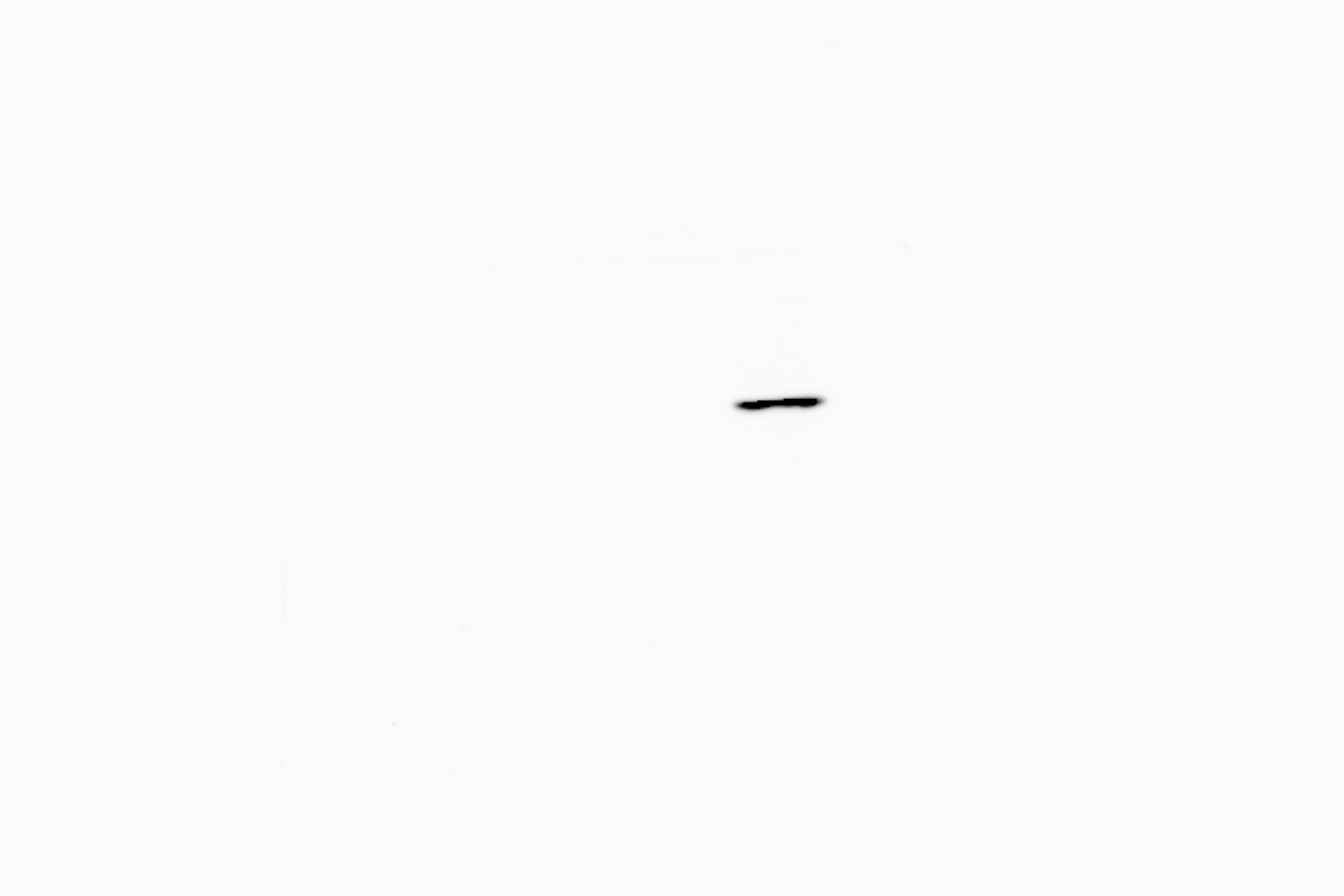

Supplement: Figure 1—source data 1. [file elife-83870-fig1-data1.zip › Figure 1-source data 1/Figure 1-source data 1 (MPI).tif]

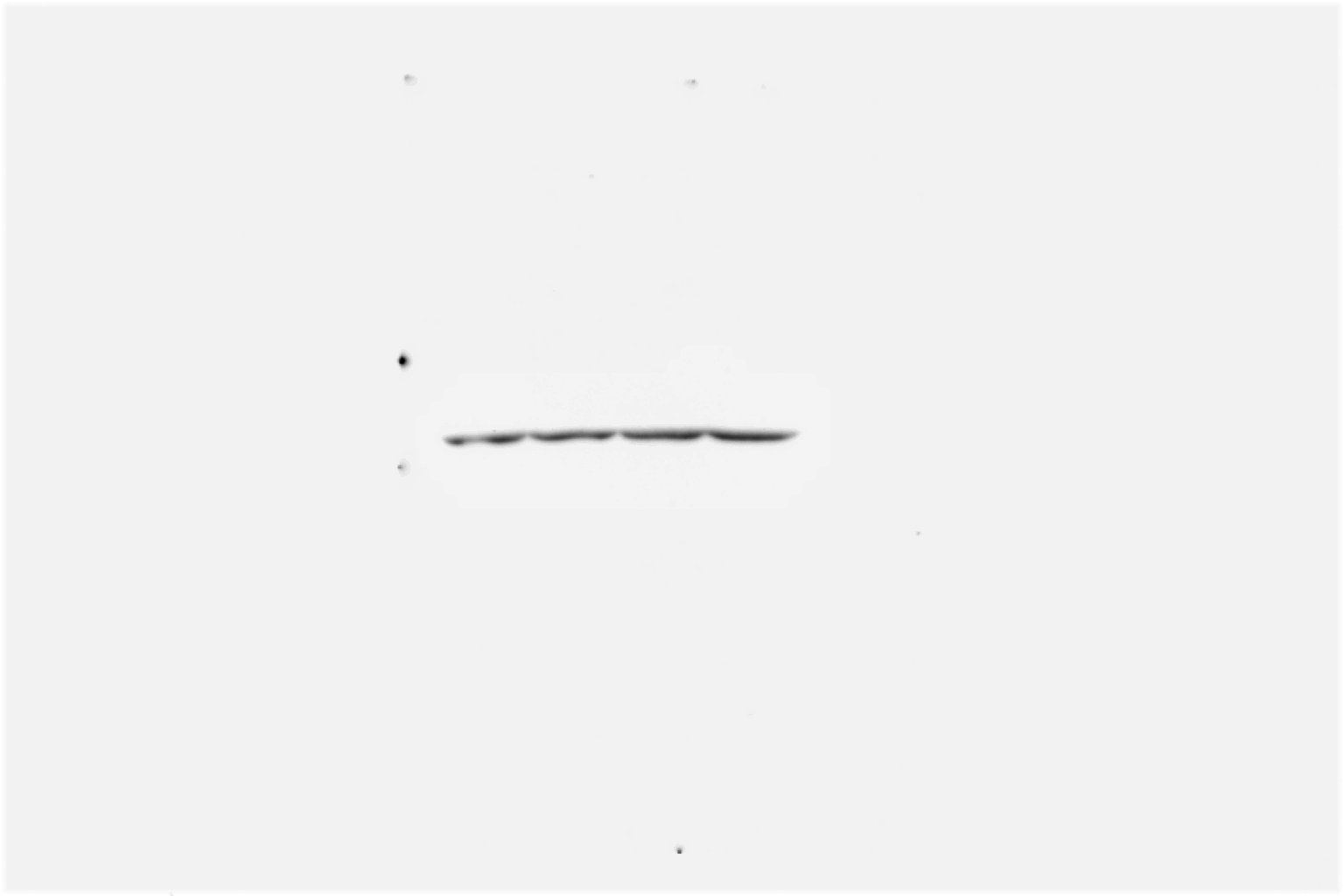

Supplement: Figure 1—source data 1. [file elife-83870-fig1-data1.zip › Figure 1-source data 1/Figure 1-source data 1 (ACTB)_flipped.tif]

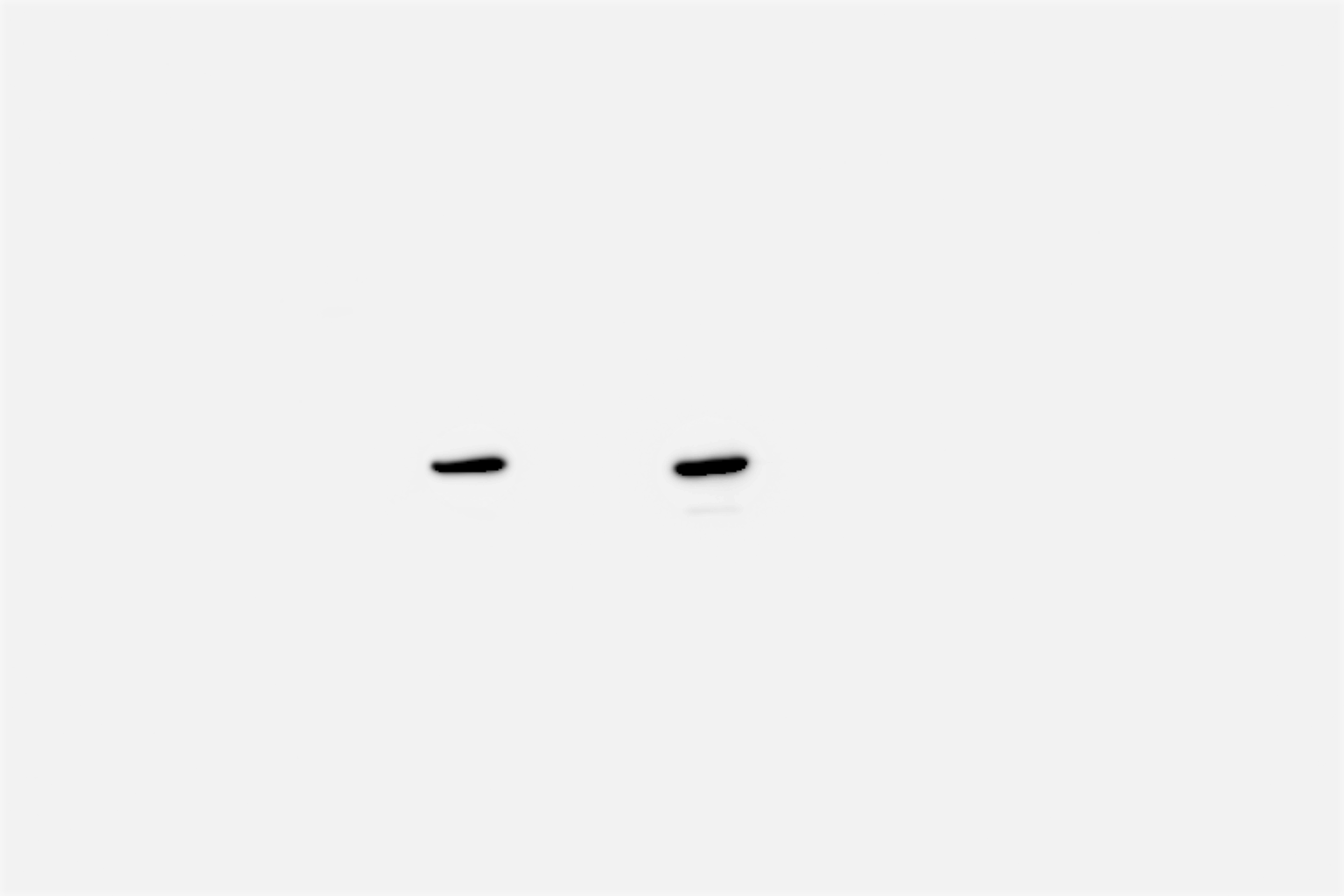

Supplement: Figure 1—source data 2. [file elife-83870-fig1-data2.zip › Figure 1-source data 2/Figure 1-source data 2 (MPI).tif]

Figure 1-source data 2

full raw unedited blots ( $\beta$ -Actin)

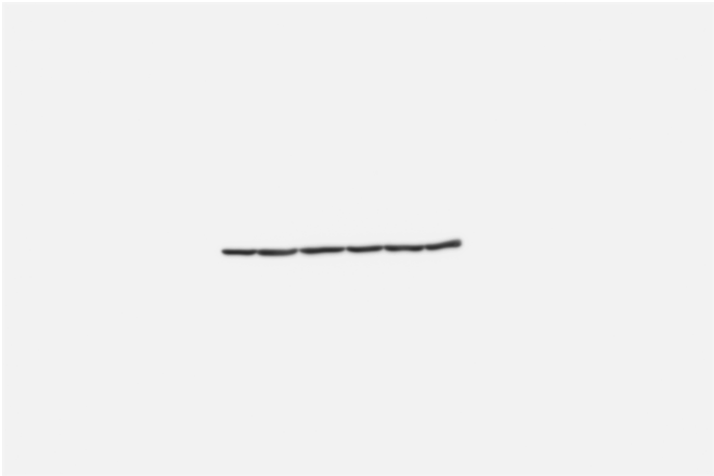

full raw unedited blots (MPI)

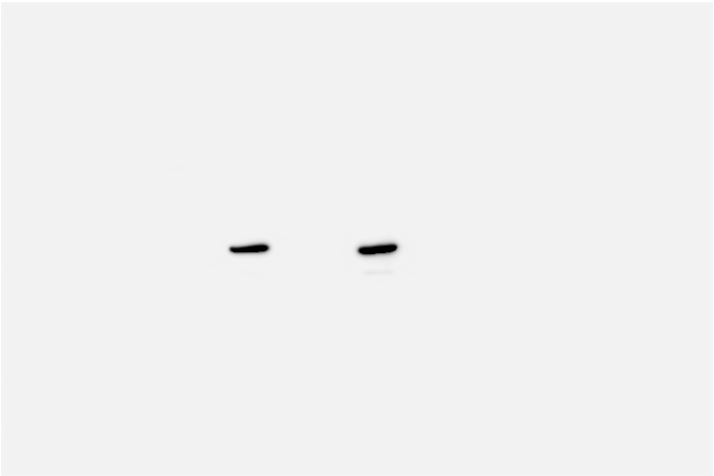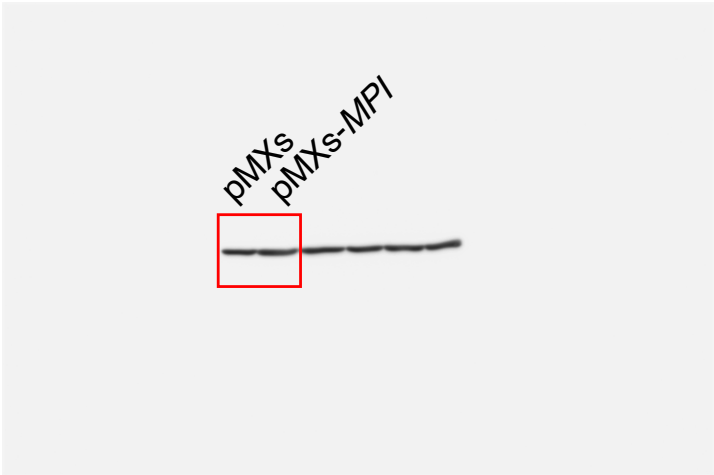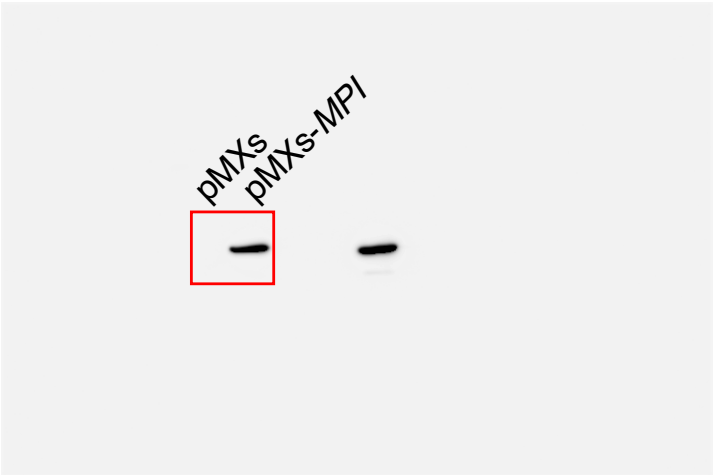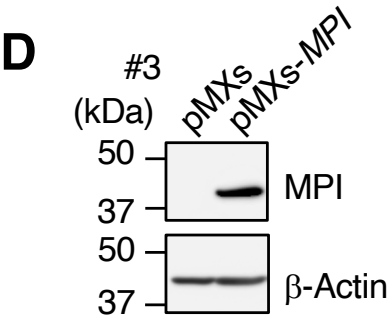

Supplement: Figure 1—source data 2. [file elife-83870-fig1-data2.zip › Figure 1-source data 2/Figure 1-source data 2 .pdf]

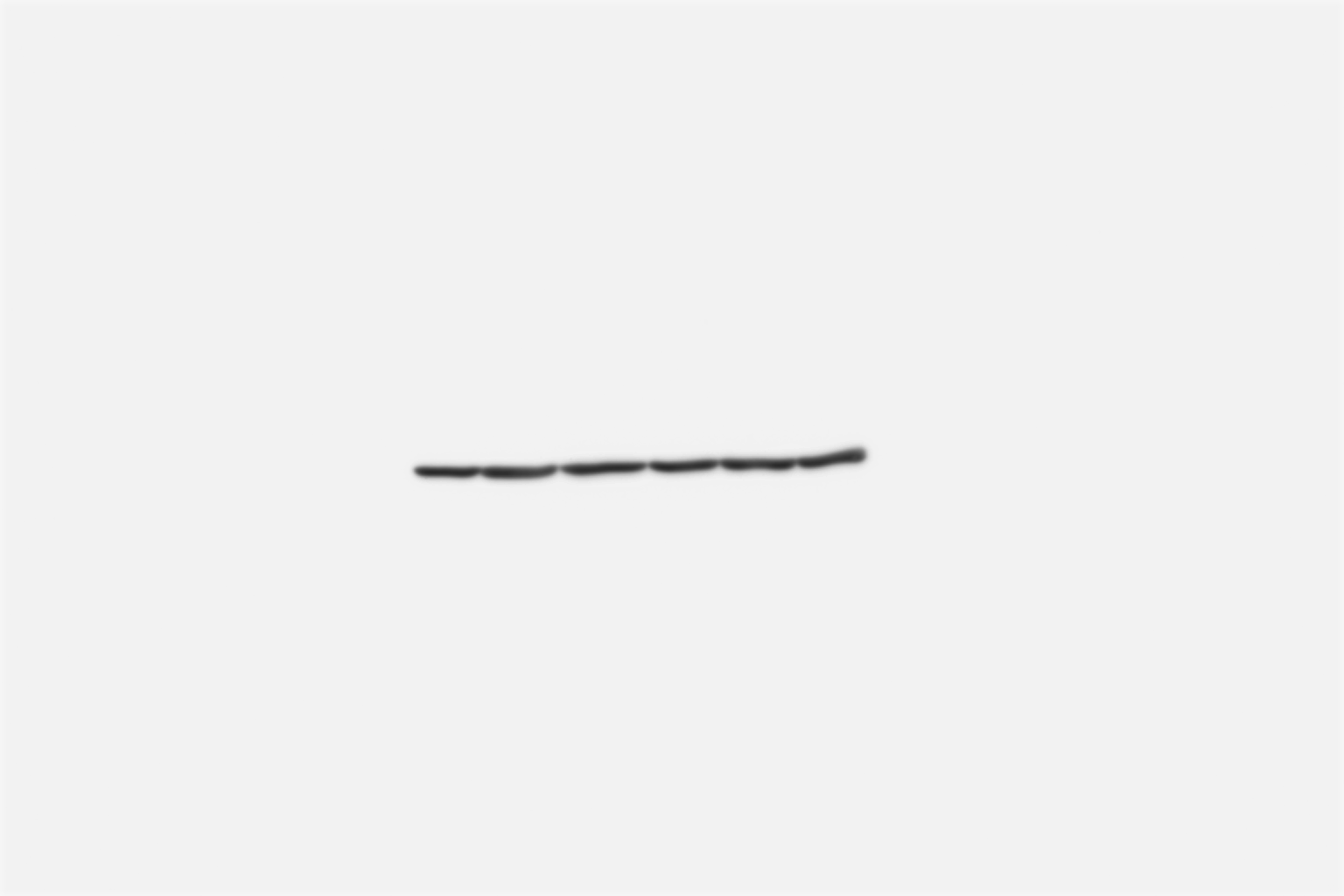

Supplement: Figure 1—source data 2. [file elife-83870-fig1-data2.zip › Figure 1-source data 2/Figure 1-source data 2 (ACTB).tif]

Figure 1-source data 3

full raw unedited blots ( $\beta$ -Actin)

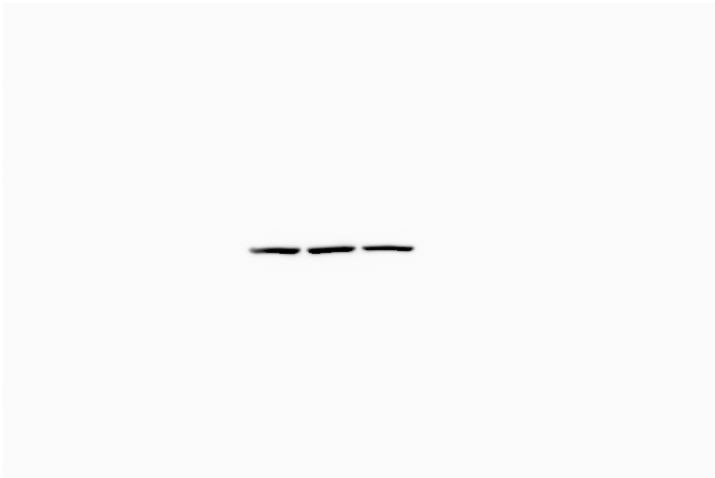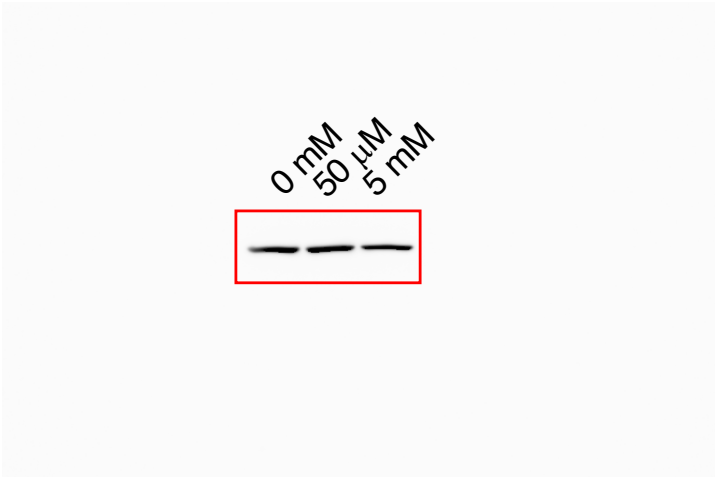

full raw unedited blots (Con A)

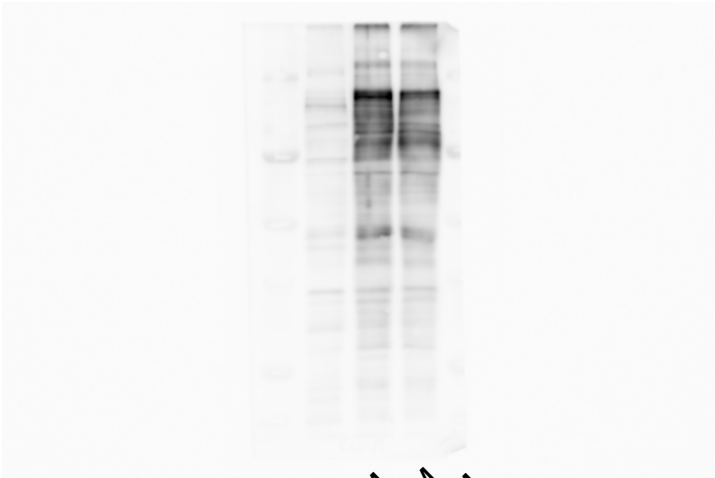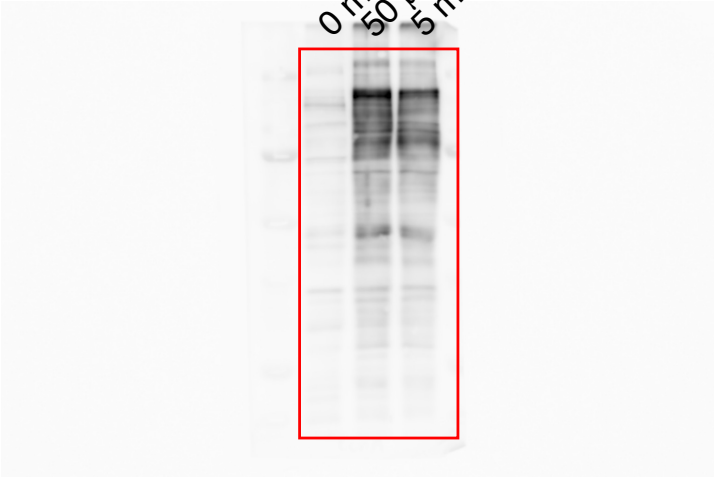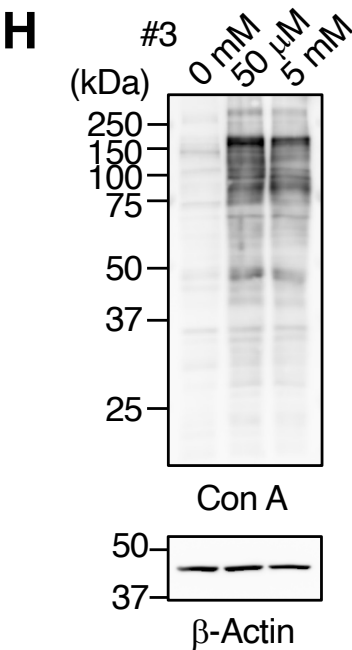

Supplement: Figure 1—source data 3. [file elife-83870-fig1-data3.zip › Figure 1-source data 3/Figure 1-source data 3.pdf]

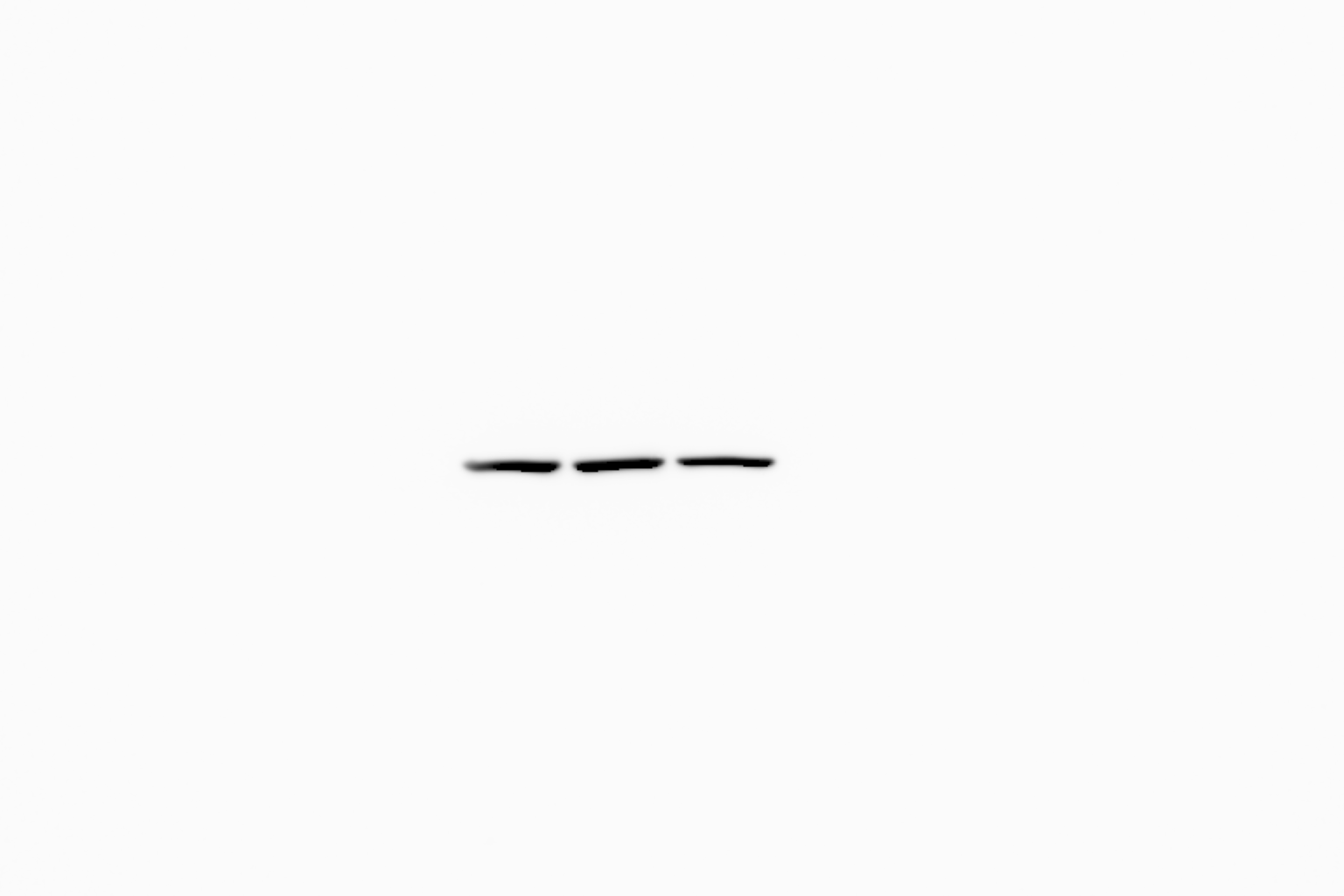

Supplement: Figure 1—source data 3. [file elife-83870-fig1-data3.zip › Figure 1-source data 3/Figure 1-source data 3 (ACTB).tif]

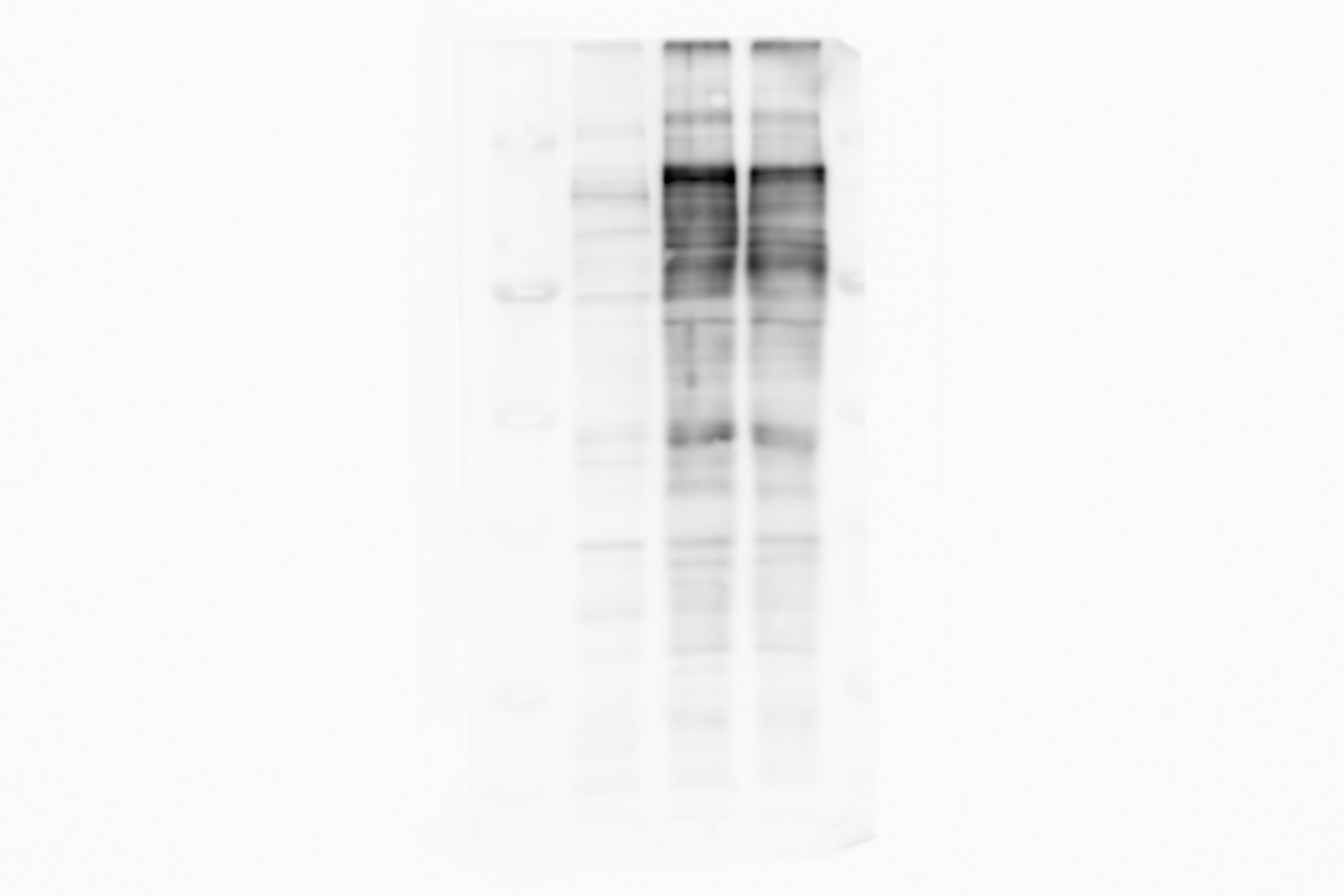

Supplement: Figure 1—source data 3. [file elife-83870-fig1-data3.zip › Figure 1-source data 3/Figure 1-source data 3 (ConA).tif]

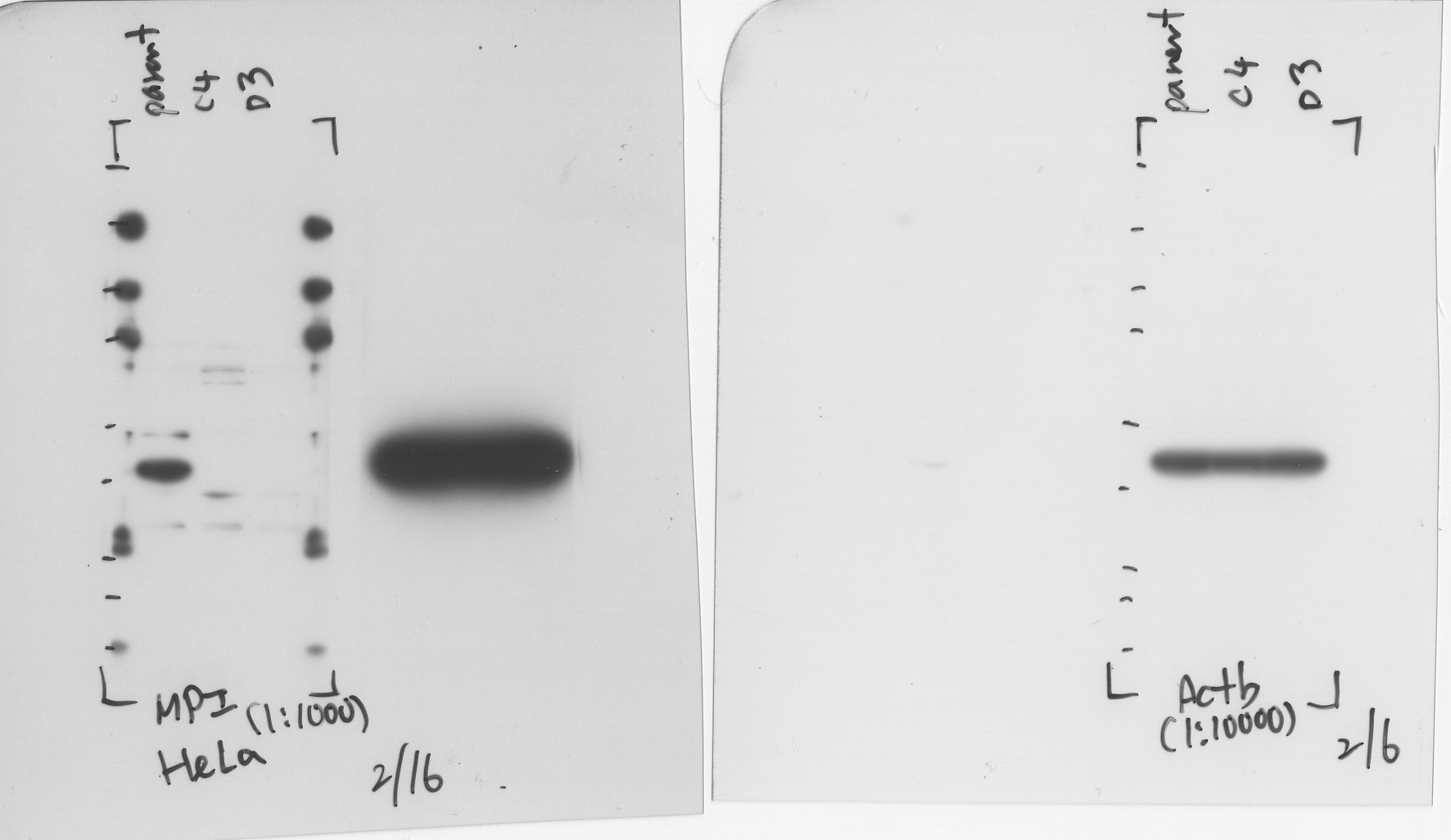

Supplement: Figure 1—figure supplement 3—source data 1. [file elife-83870-fig1-figsupp3-data1.zip › Figure 1-figure supplement 3-source data 1/Figure 1-figure supplement 2-source data 1 (MPI_ACTB).tif]

Figure 1-figure supplement 2-source data 1

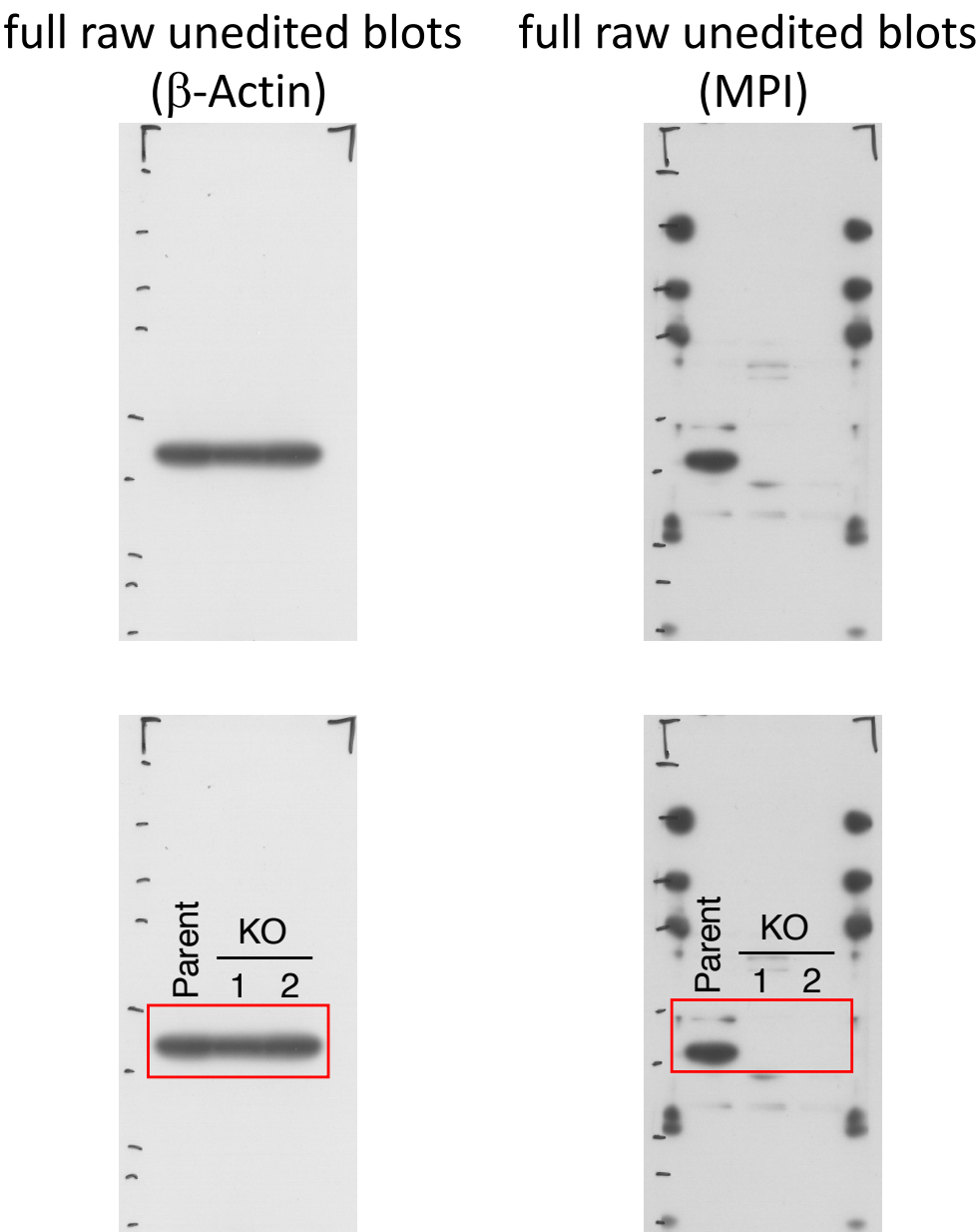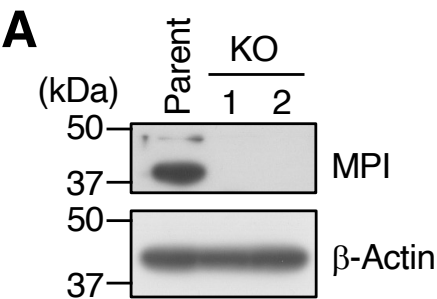

Supplement: Figure 1—figure supplement 3—source data 1. [file elife-83870-fig1-figsupp3-data1.zip › Figure 1-figure supplement 3-source data 1/Figure 1-figure supplement 2-source data 1.pdf]

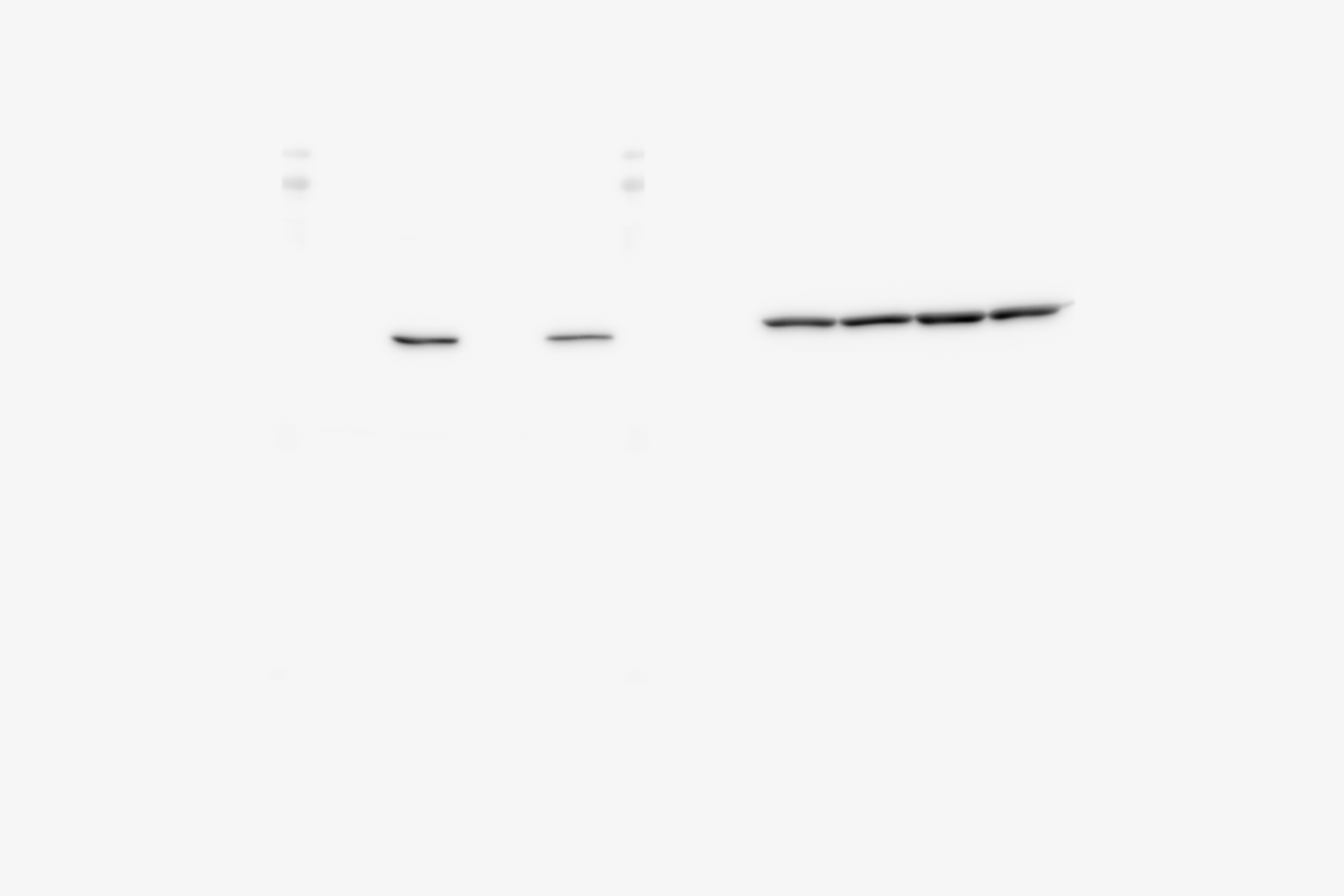

Supplement: Figure 1—figure supplement 3—source data 2. [file elife-83870-fig1-figsupp3-data2.zip › Figure 1-figure supplement 3-source data 2/Figure 1-figure supplement 2-source data 2 (MPI_ACTB).tif]

Figure 1-figure supplement 2-source data 2

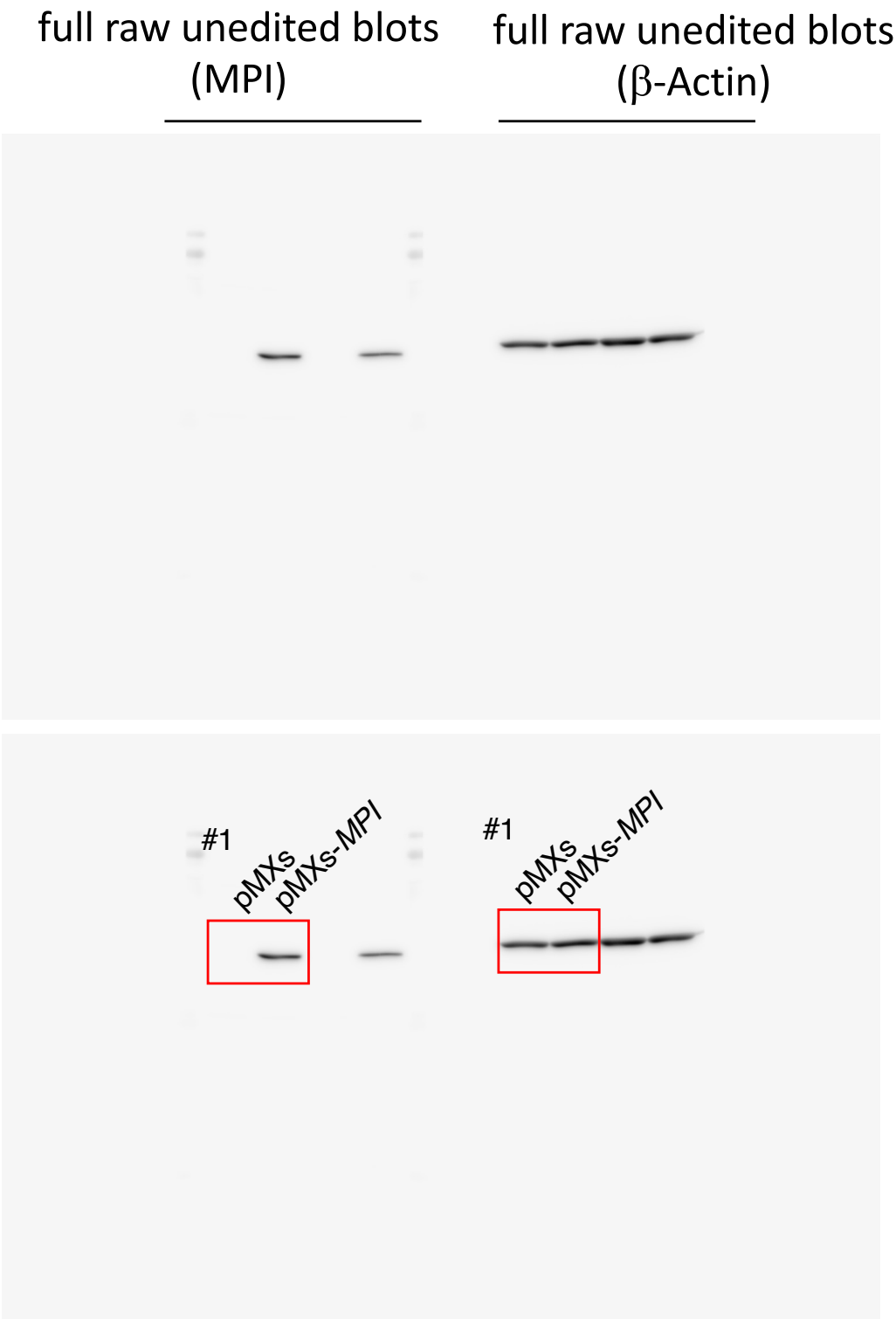

**D**

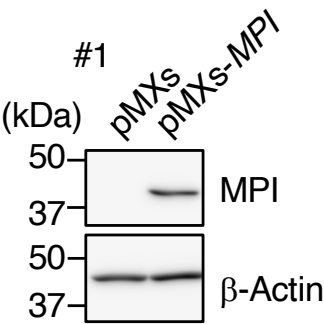

Supplement: Figure 1—figure supplement 3—source data 2. [file elife-83870-fig1-figsupp3-data2.zip › Figure 1-figure supplement 3-source data 2/Figure 1-figure supplement 2-source data 2.pdf]

Figure 1-figure supplement 2-source data 3

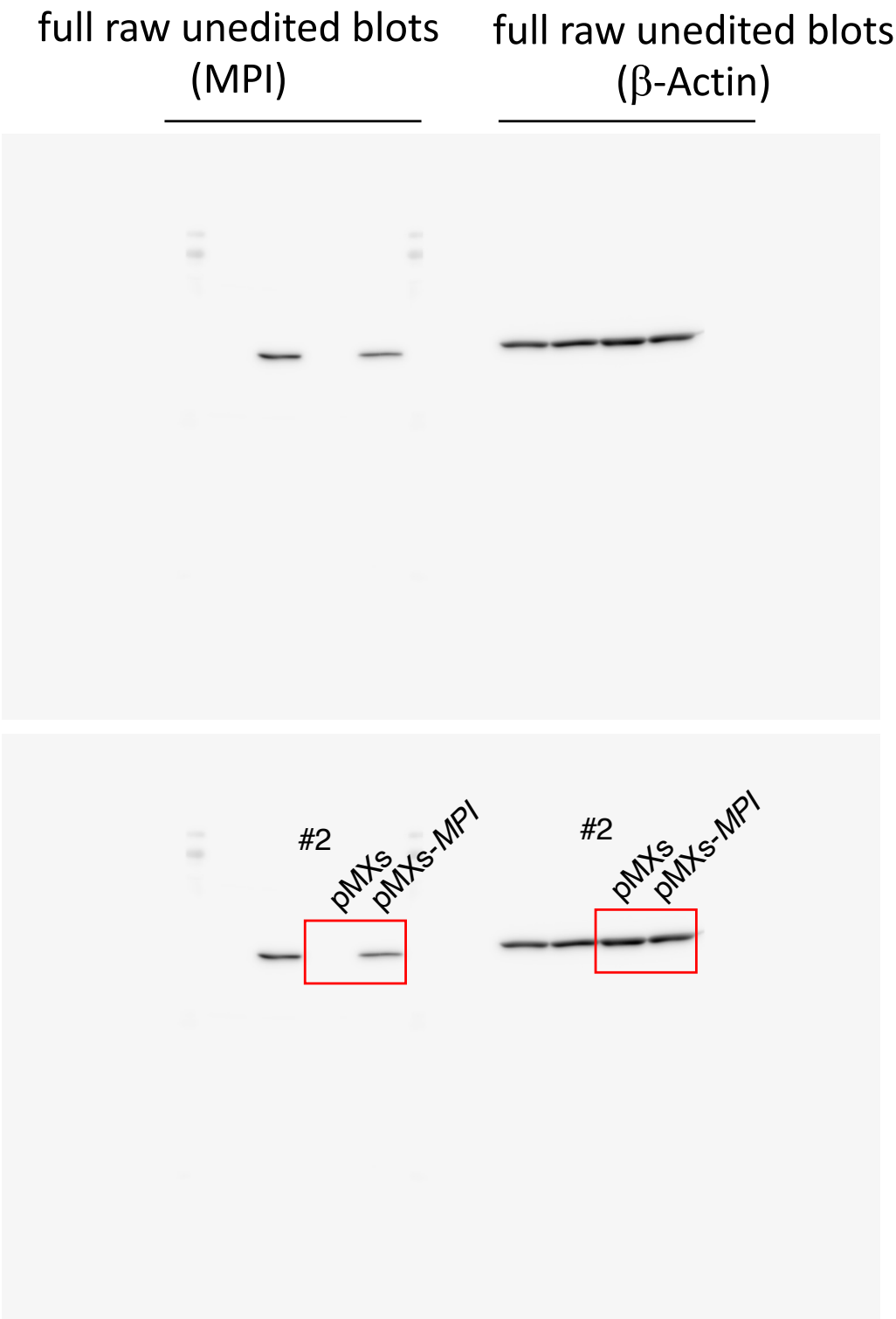

**E**

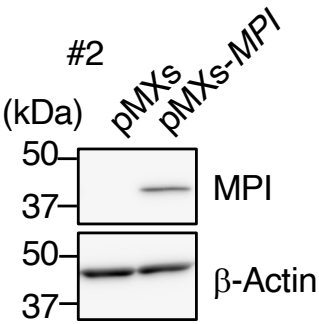

Supplement: Figure 1—figure supplement 3—source data 3. [file elife-83870-fig1-figsupp3-data3.zip › Figure 1-figure supplement 3-source data 3/Figure 1-figure supplement 2-source data 3.pdf]

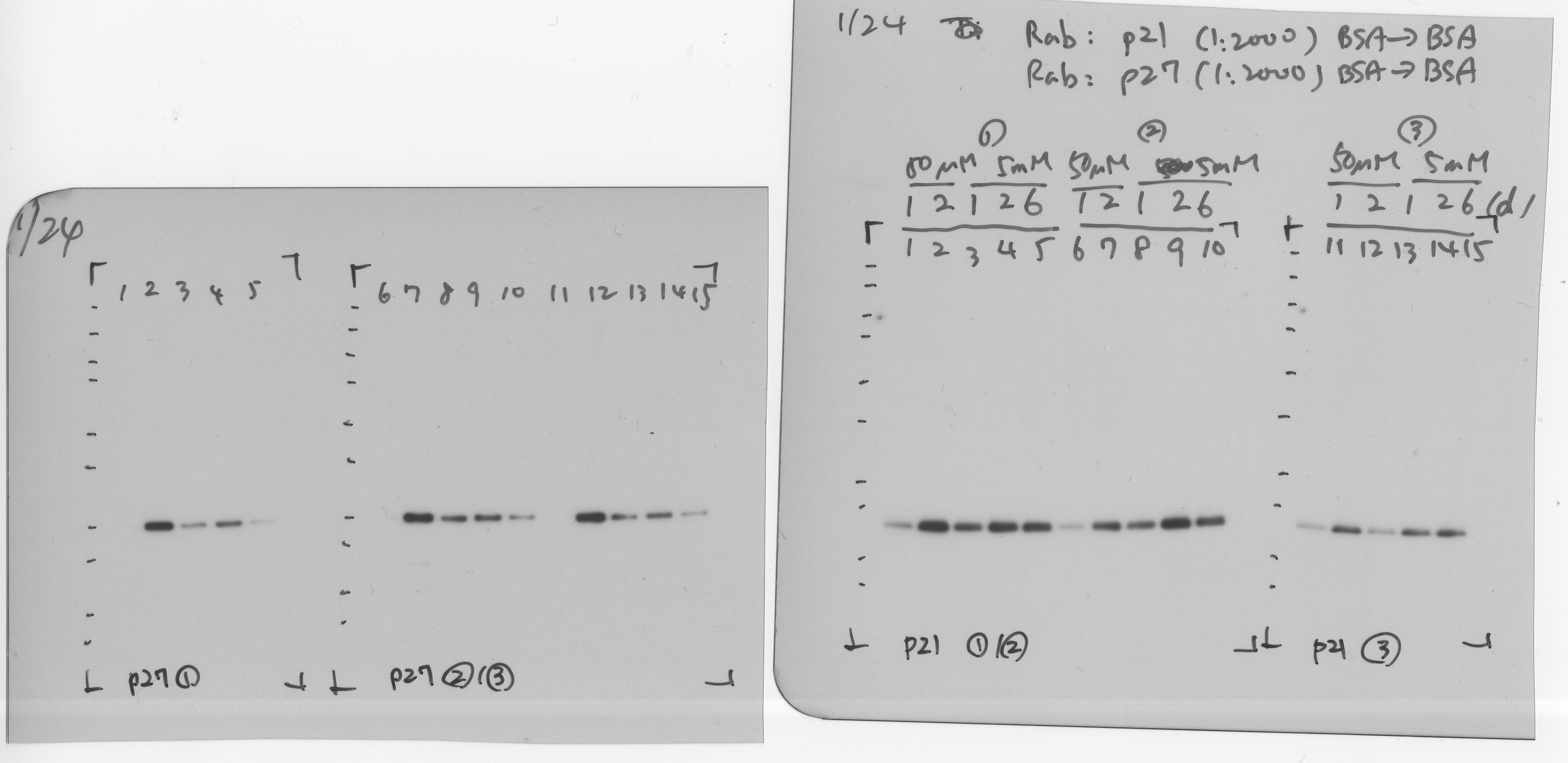

Supplement: Figure 2—source data 1. [file elife-83870-fig2-data1.zip › Figure 2-source data 1/Figure 2-source data 1 (P21_P27).tif]

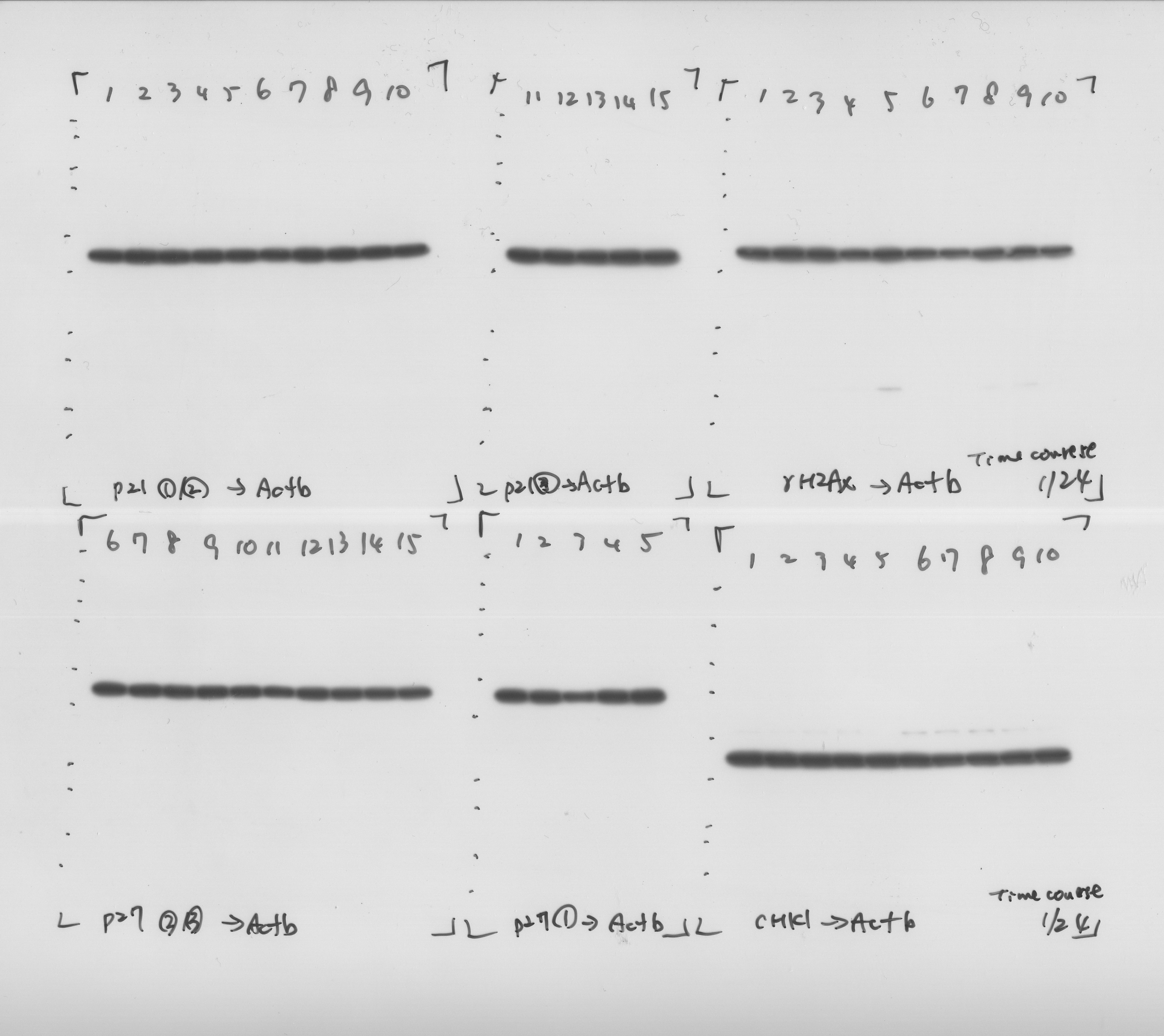

Supplement: Figure 2—source data 1. [file elife-83870-fig2-data1.zip › Figure 2-source data 1/Figure 2-source data 1 (ACT).tif]

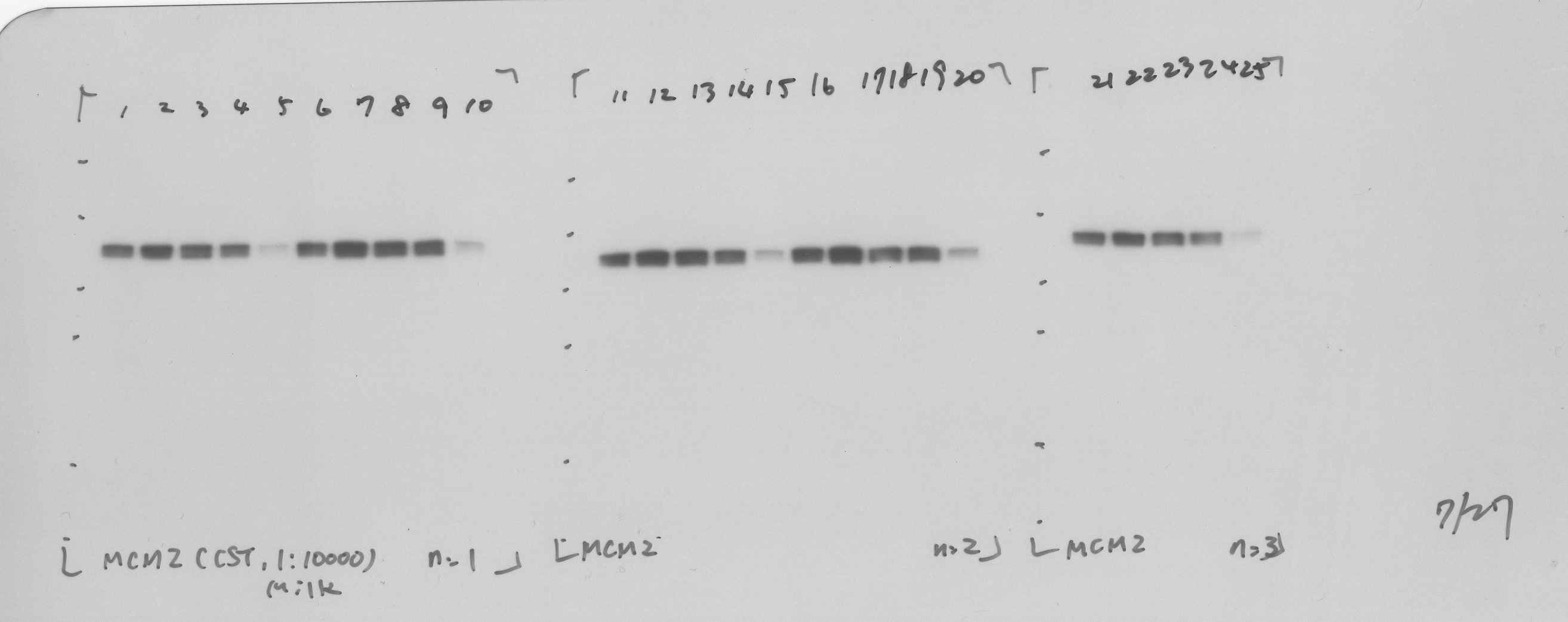

Supplement: Figure 3—source data 1. [file elife-83870-fig3-data1.zip › Figure 3-source data 1/Figure 3-source data 1 (MCM2).tif]

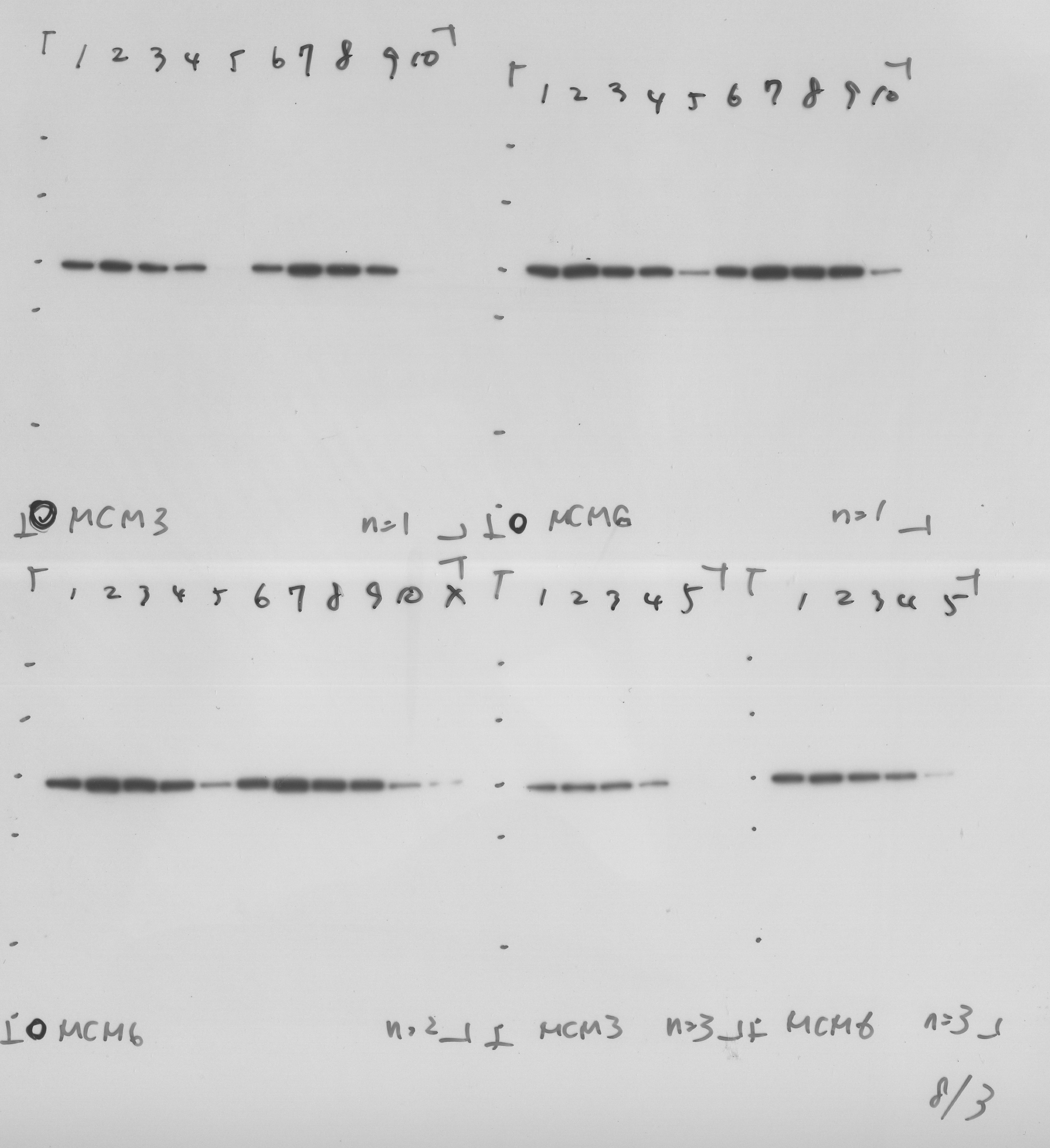

Supplement: Figure 3—source data 1. [file elife-83870-fig3-data1.zip › Figure 3-source data 1/Figure 3-source data 1 (MCM3).tif]

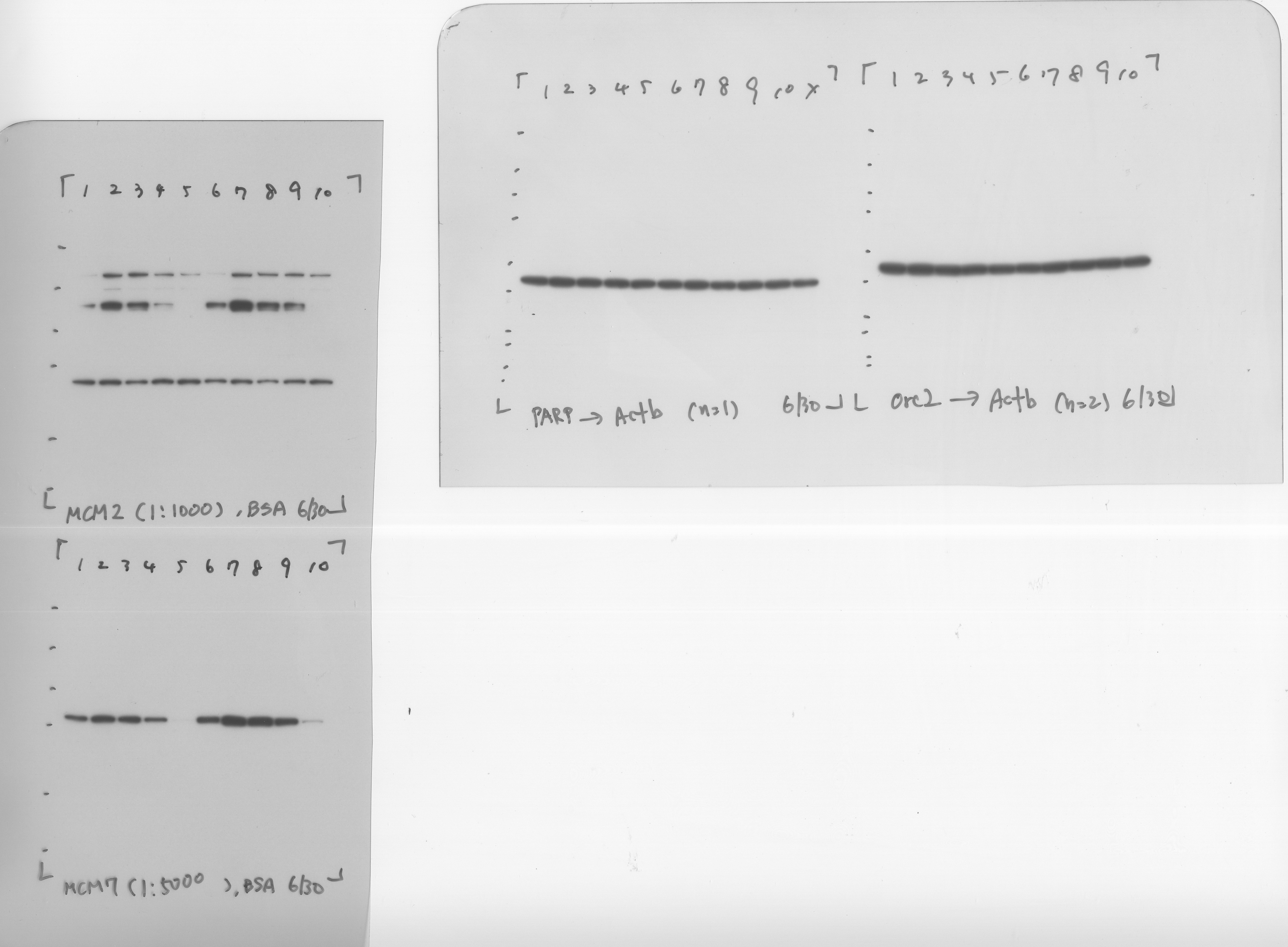

Supplement: Figure 3—source data 1. [file elife-83870-fig3-data1.zip › Figure 3-source data 1/Figure 3-source data 1 (ACTB_reprobe from PARP).tif]

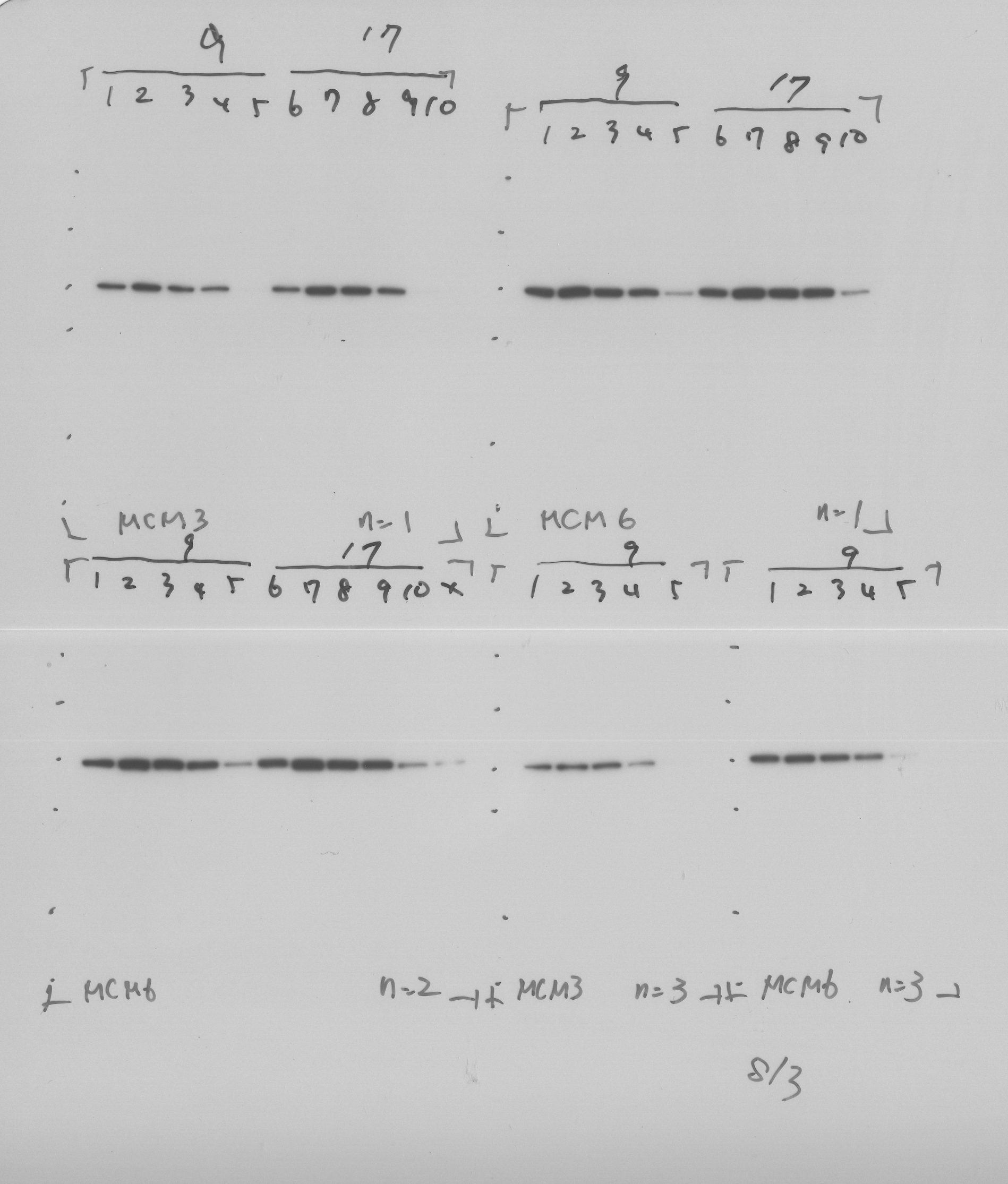

Supplement: Figure 3—source data 2. [file elife-83870-fig3-data2.zip › Figure 3-source data 2/Figure 3-source data 2 (MCM6).tif]

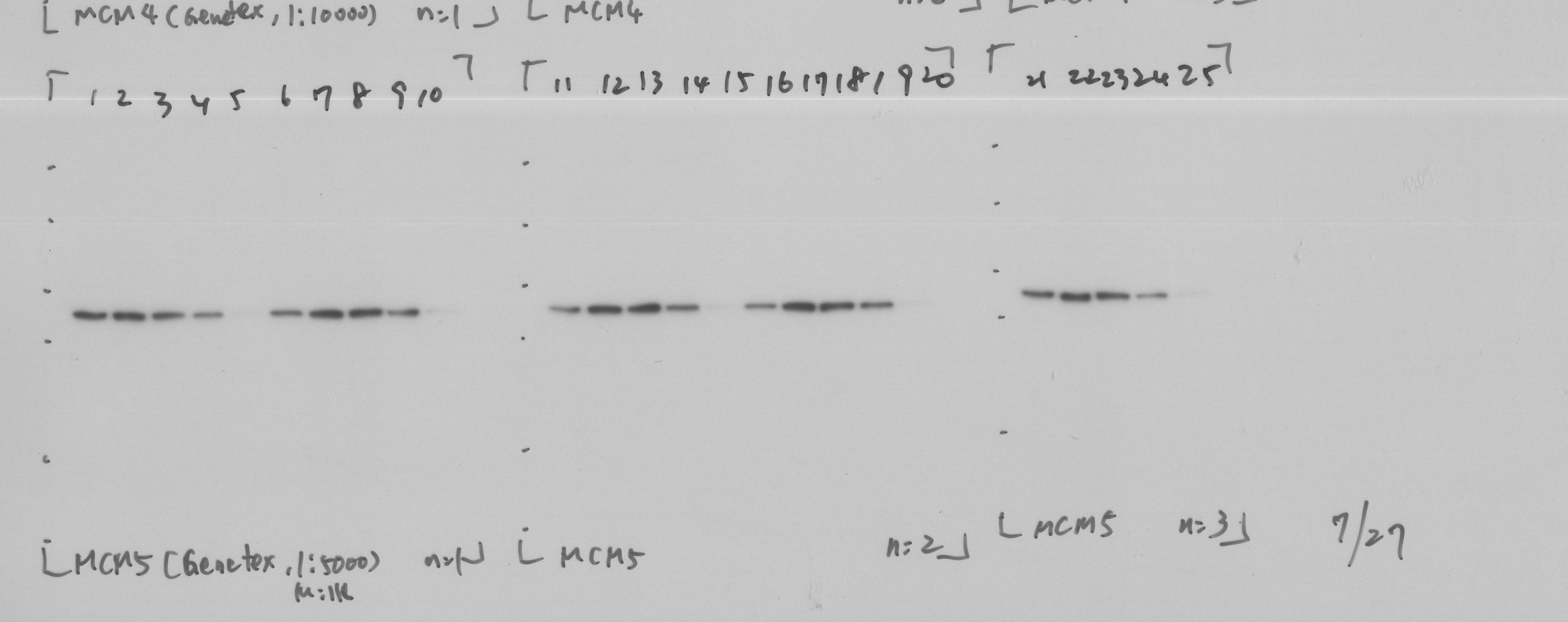

Supplement: Figure 3—source data 2. [file elife-83870-fig3-data2.zip › Figure 3-source data 2/Figure 3-source data 2 (MCM5).tif]

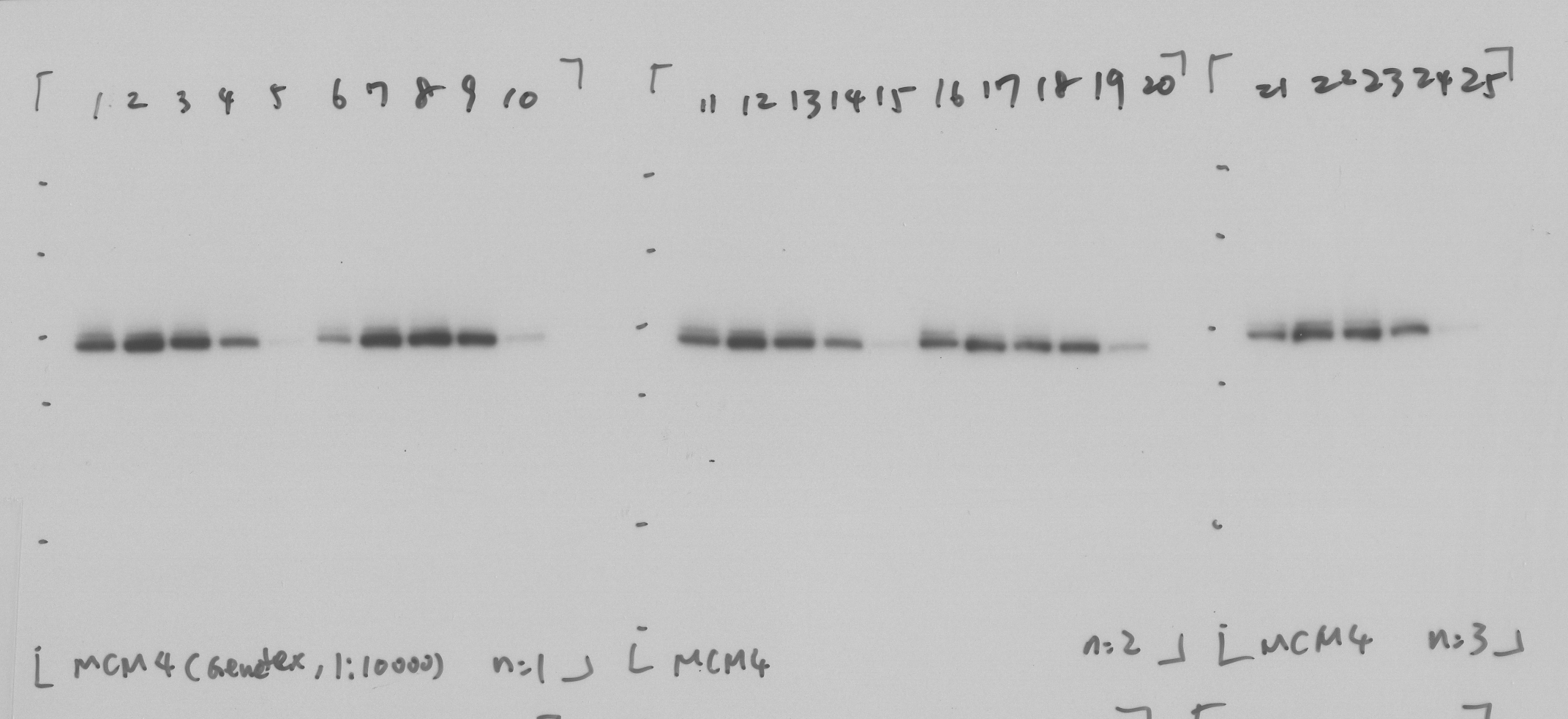

Supplement: Figure 3—source data 2. [file elife-83870-fig3-data2.zip › Figure 3-source data 2/Figure 3-source data 2 (MCM4).tif]

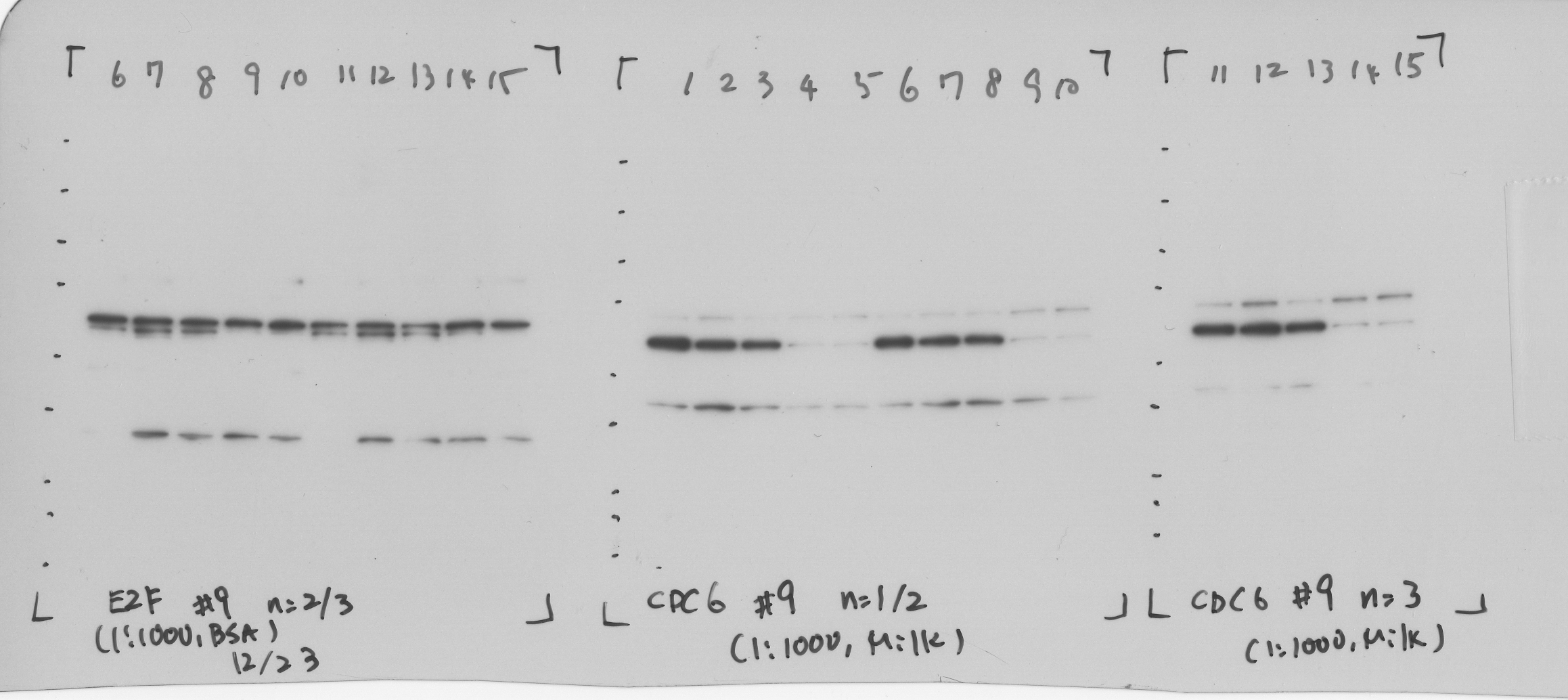

Supplement: Figure 3—source data 3. [file elife-83870-fig3-data3.zip › Figure 3-source data 3/Figure 3-source data 3 (CDC6).tif]

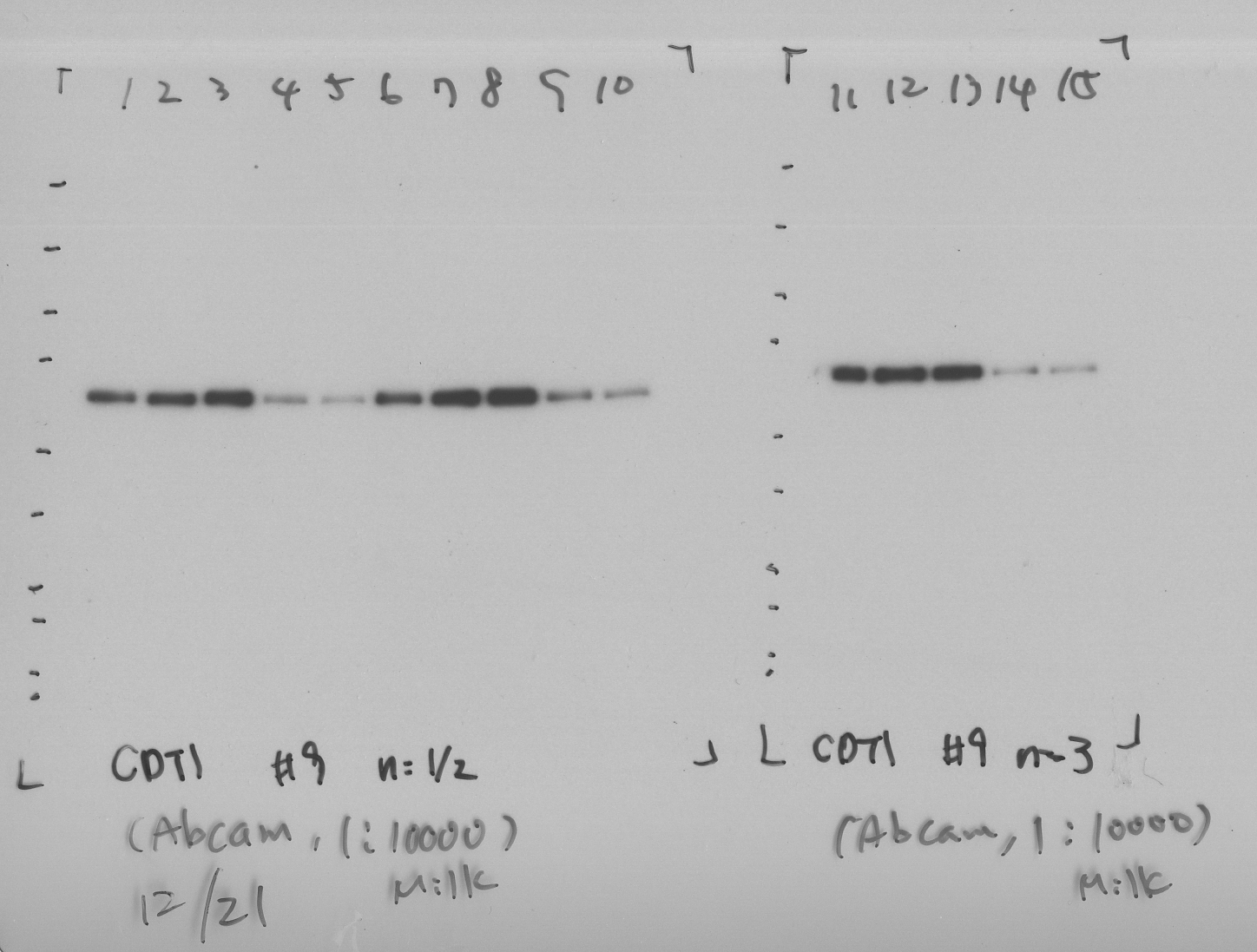

Supplement: Figure 3—source data 3. [file elife-83870-fig3-data3.zip › Figure 3-source data 3/Figure 3-source data 3 (CDT1).tif]

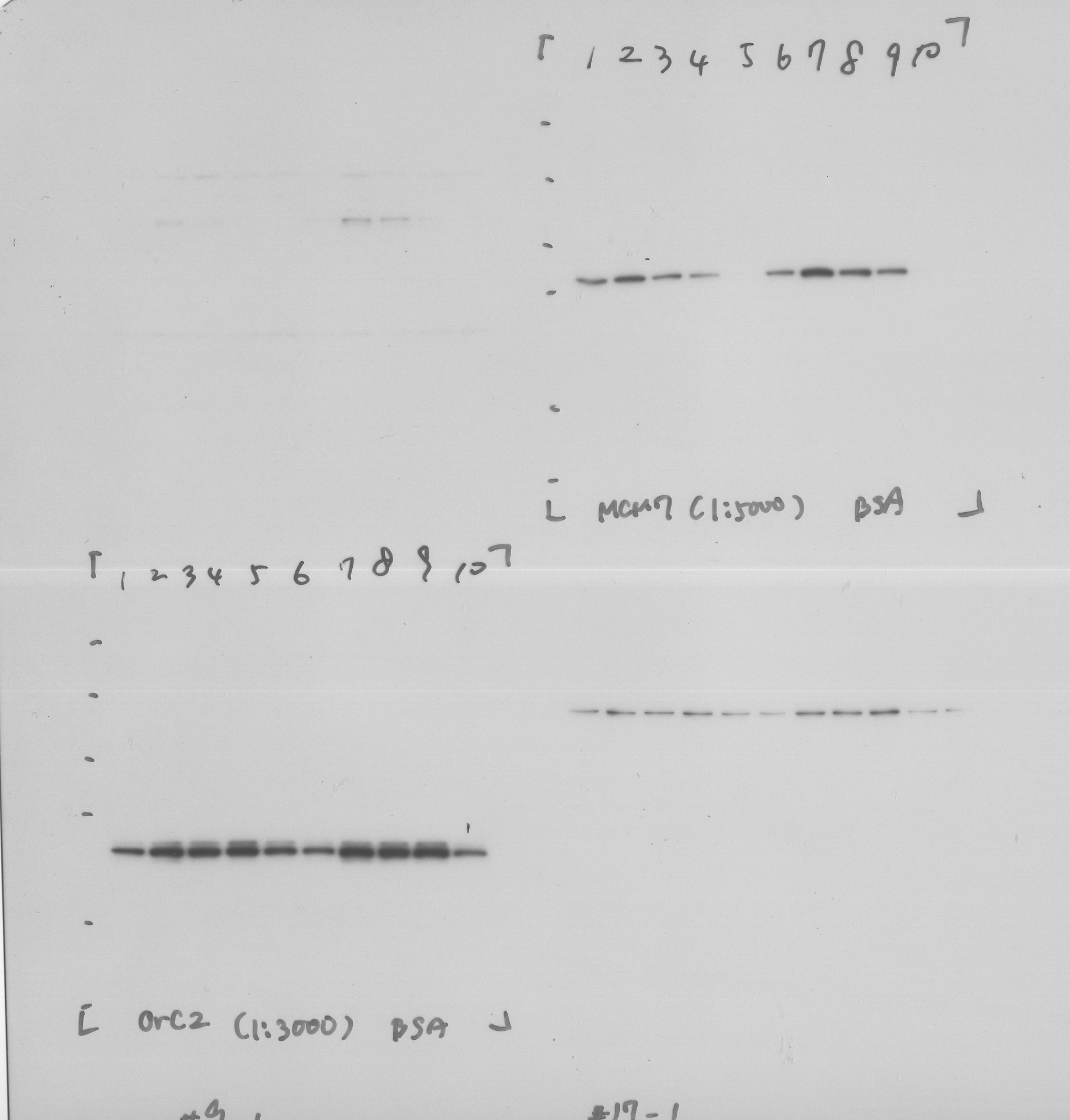

Supplement: Figure 3—source data 4. [file elife-83870-fig3-data4.zip › Figure 3-source data 4/Figure 3-source data 4.tif]

Figure 3-source data 4

full raw unedited blots  
(ORC2)

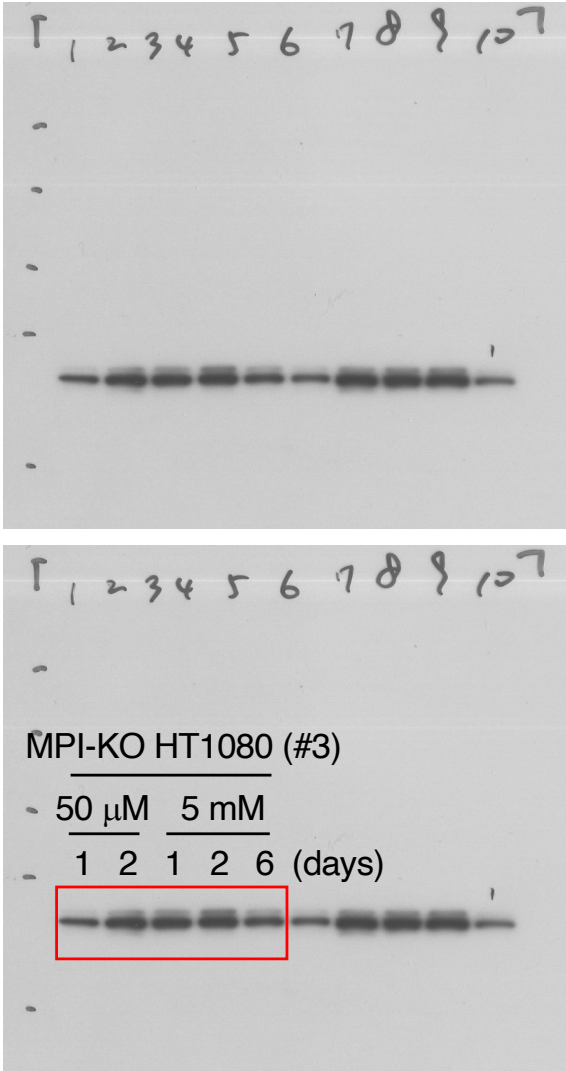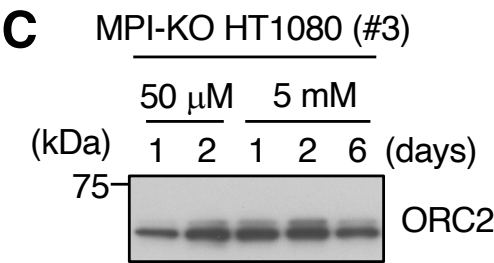

Supplement: Figure 3—source data 4. [file elife-83870-fig3-data4.zip › Figure 3-source data 4/Figure 3-source data 4.pdf]

Figure 4-source data 1

Whole cell lysate

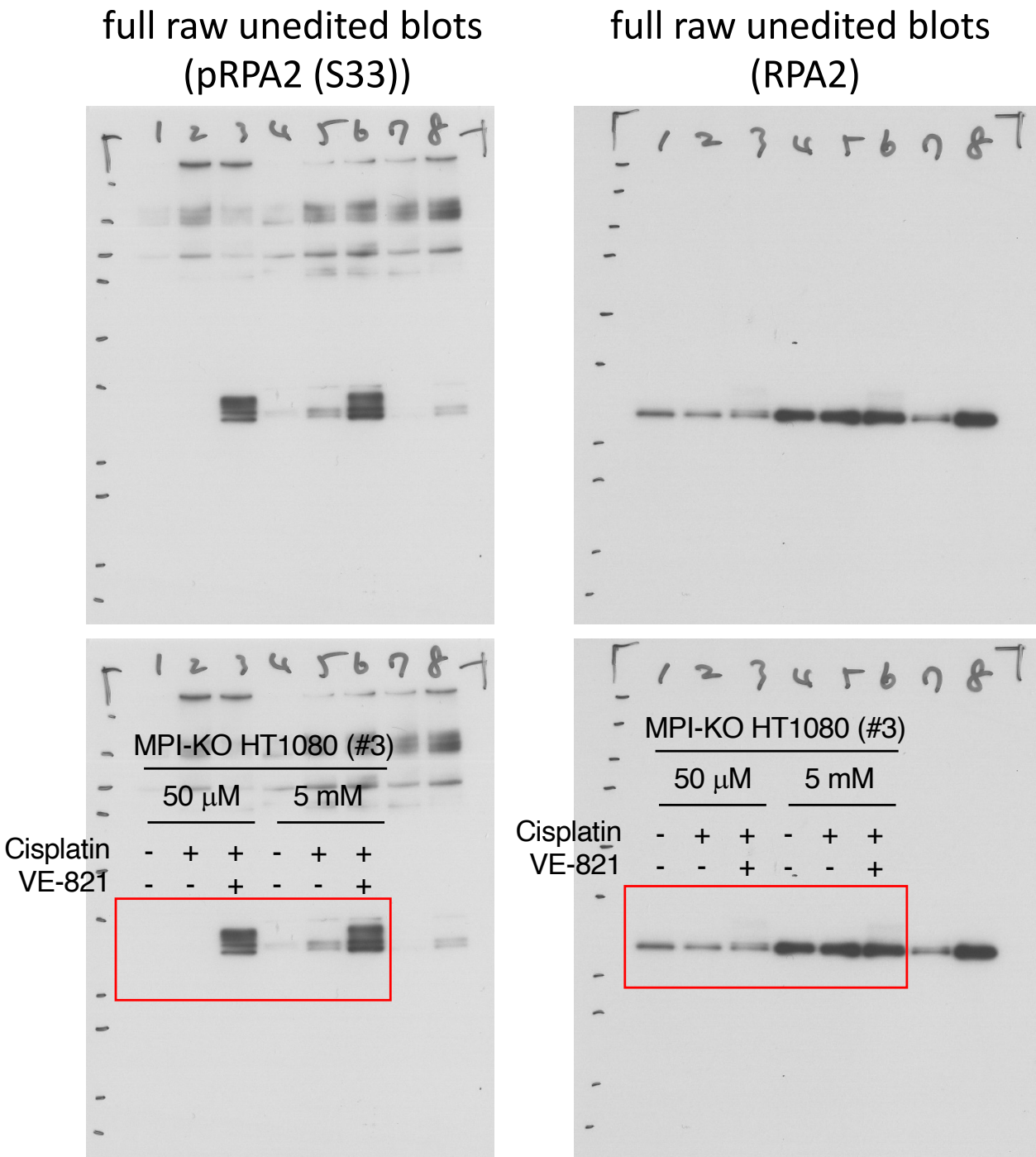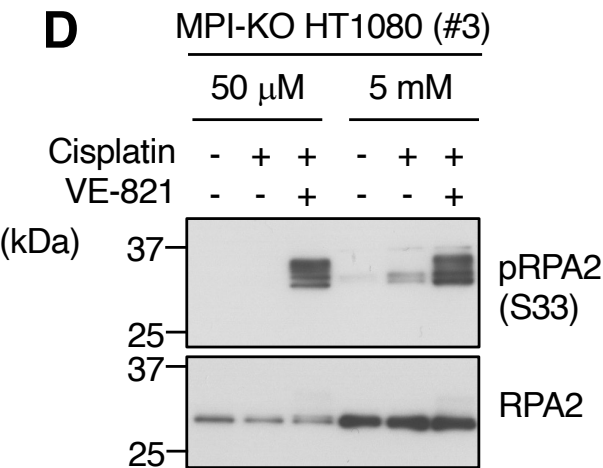

Supplement: Figure 4—source data 1. [file elife-83870-fig4-data1.zip › Figure 4-source data 1/Figure 4-source data 1.pdf]

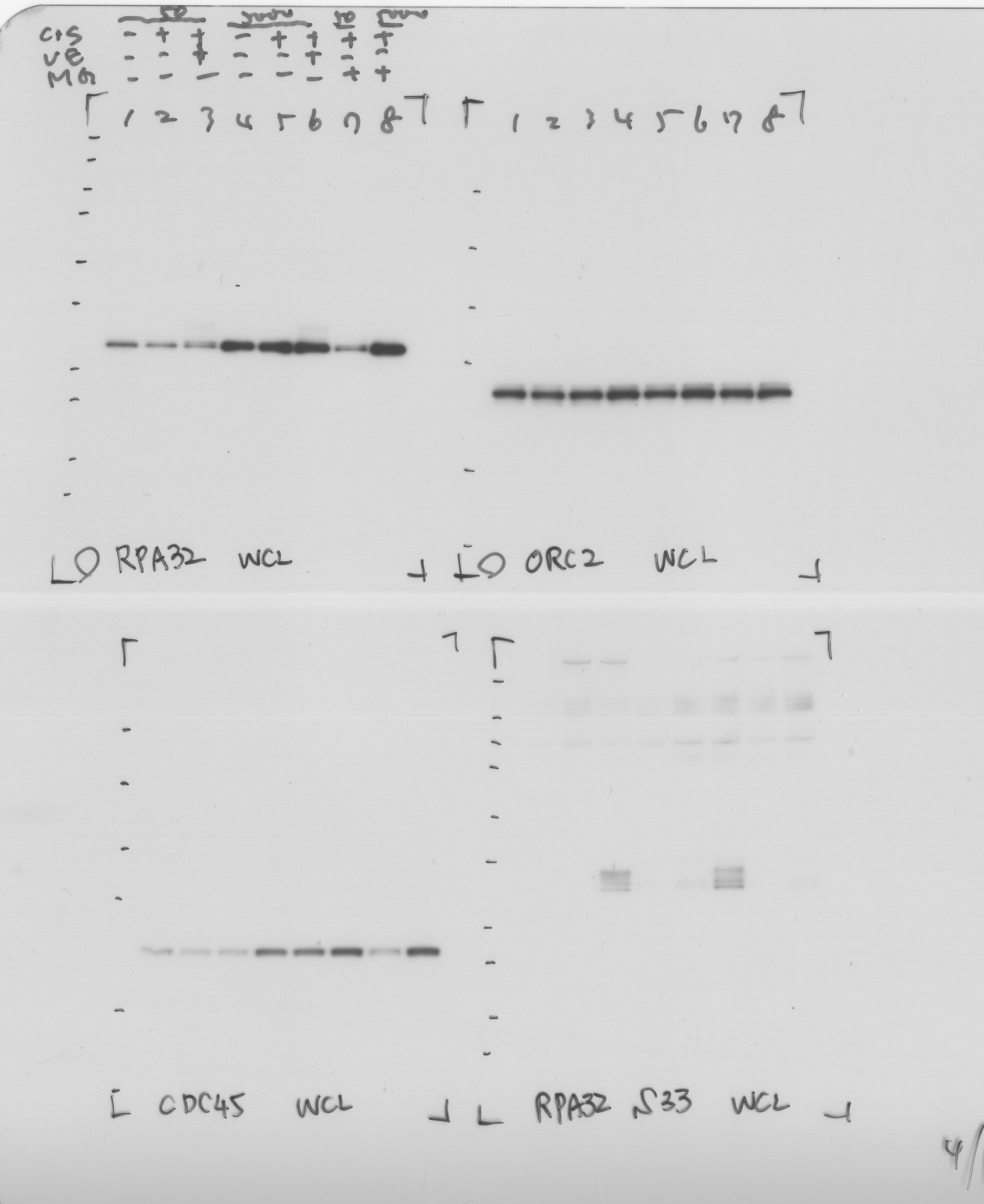

Supplement: Figure 4—source data 1. [file elife-83870-fig4-data1.zip › Figure 4-source data 1/Figure 4-source data 1 (RPA2).tif]

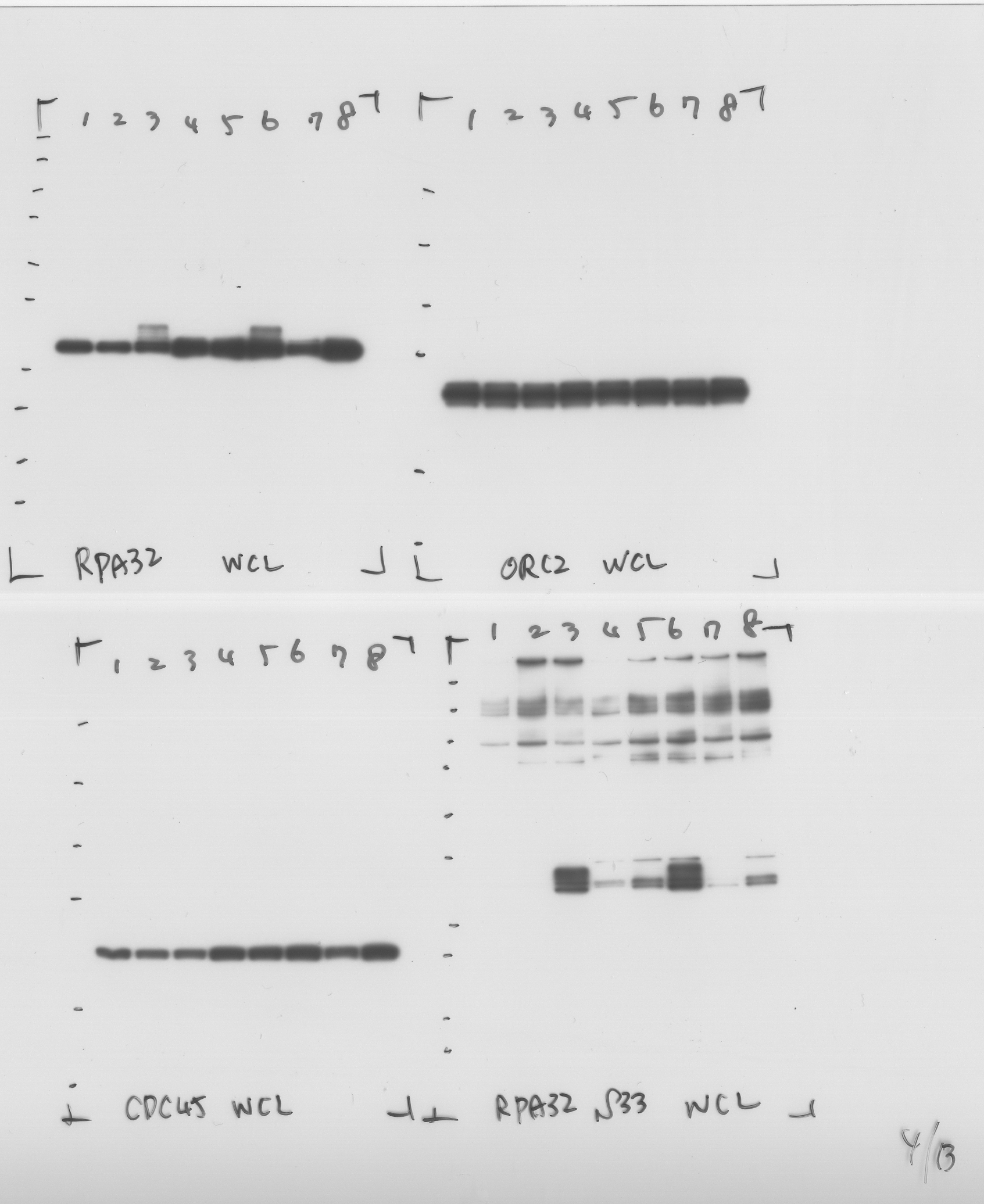

Supplement: Figure 4—source data 1. [file elife-83870-fig4-data1.zip › Figure 4-source data 1/Figure 4-source data 1 (pRPA (S33)).tif]

Figure 4-source data 2

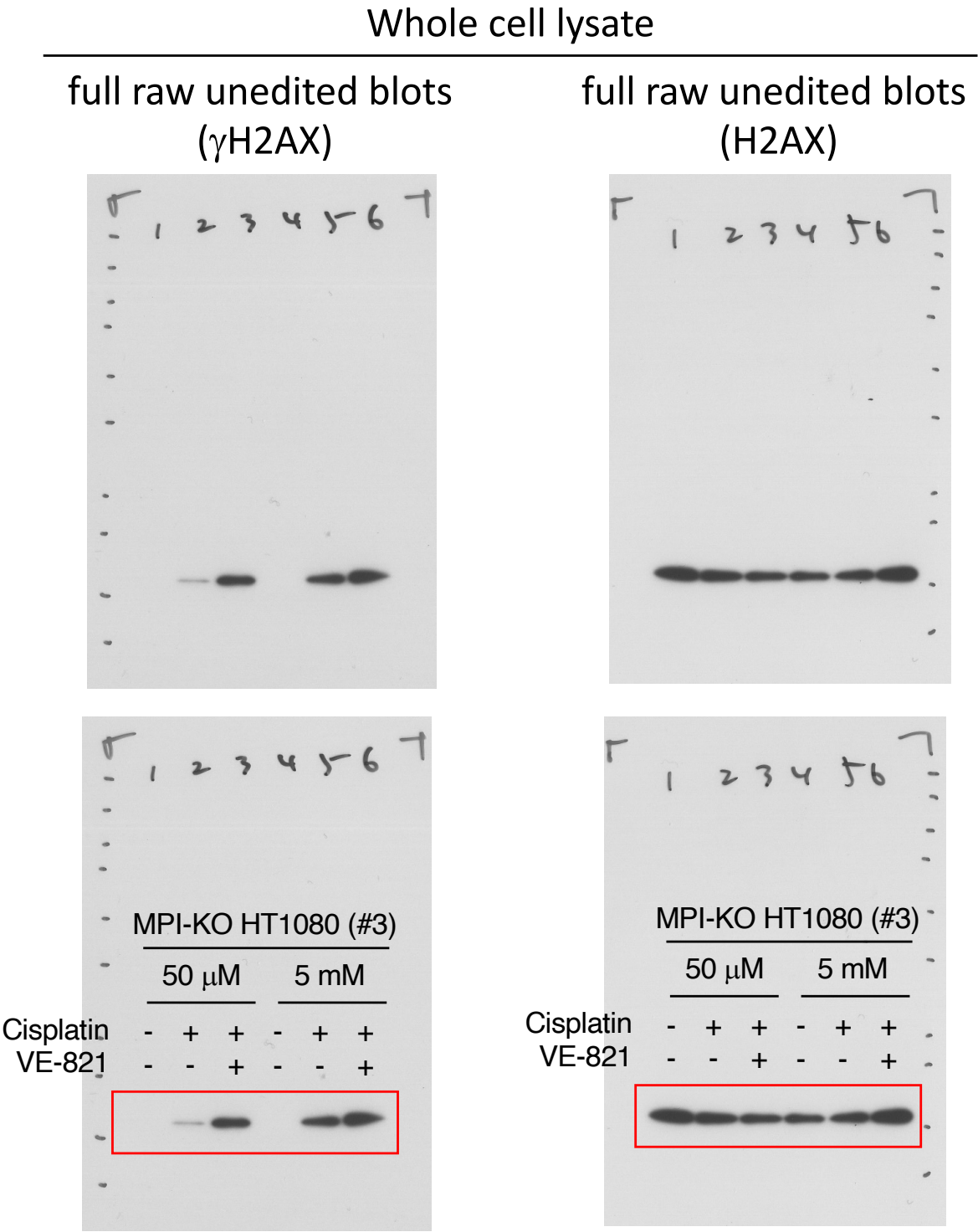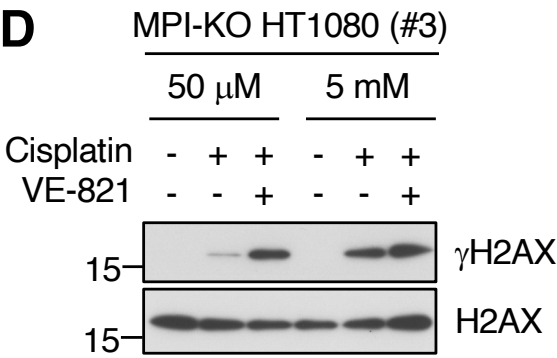

Supplement: Figure 4—source data 2. [file elife-83870-fig4-data2.zip › Figure 4-source data 2/Figure 4-source data 2.pdf]

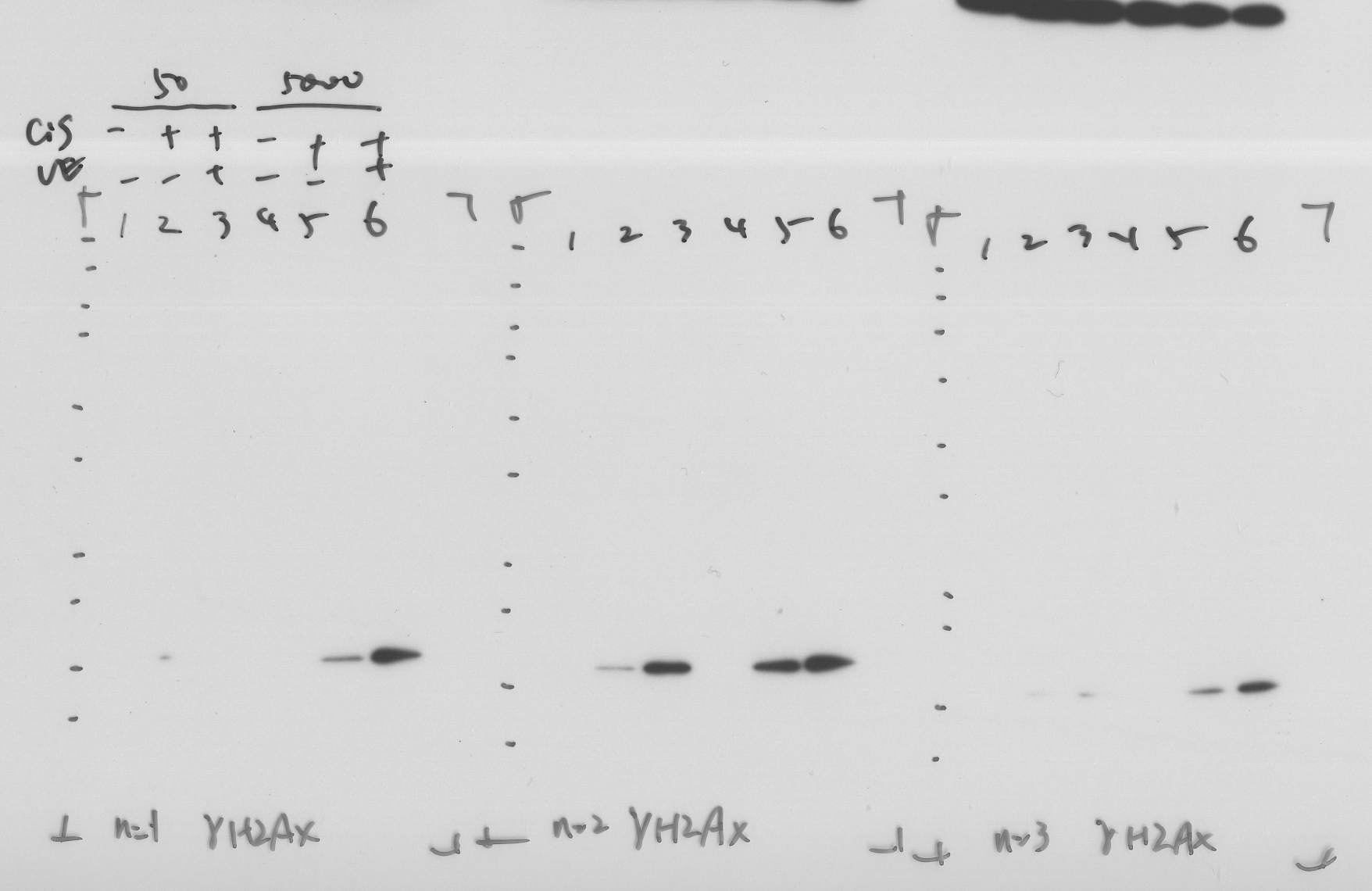

Supplement: Figure 4—source data 2. [file elife-83870-fig4-data2.zip › Figure 4-source data 2/Figure 4-source data 2 (gammaH2AX).tif]

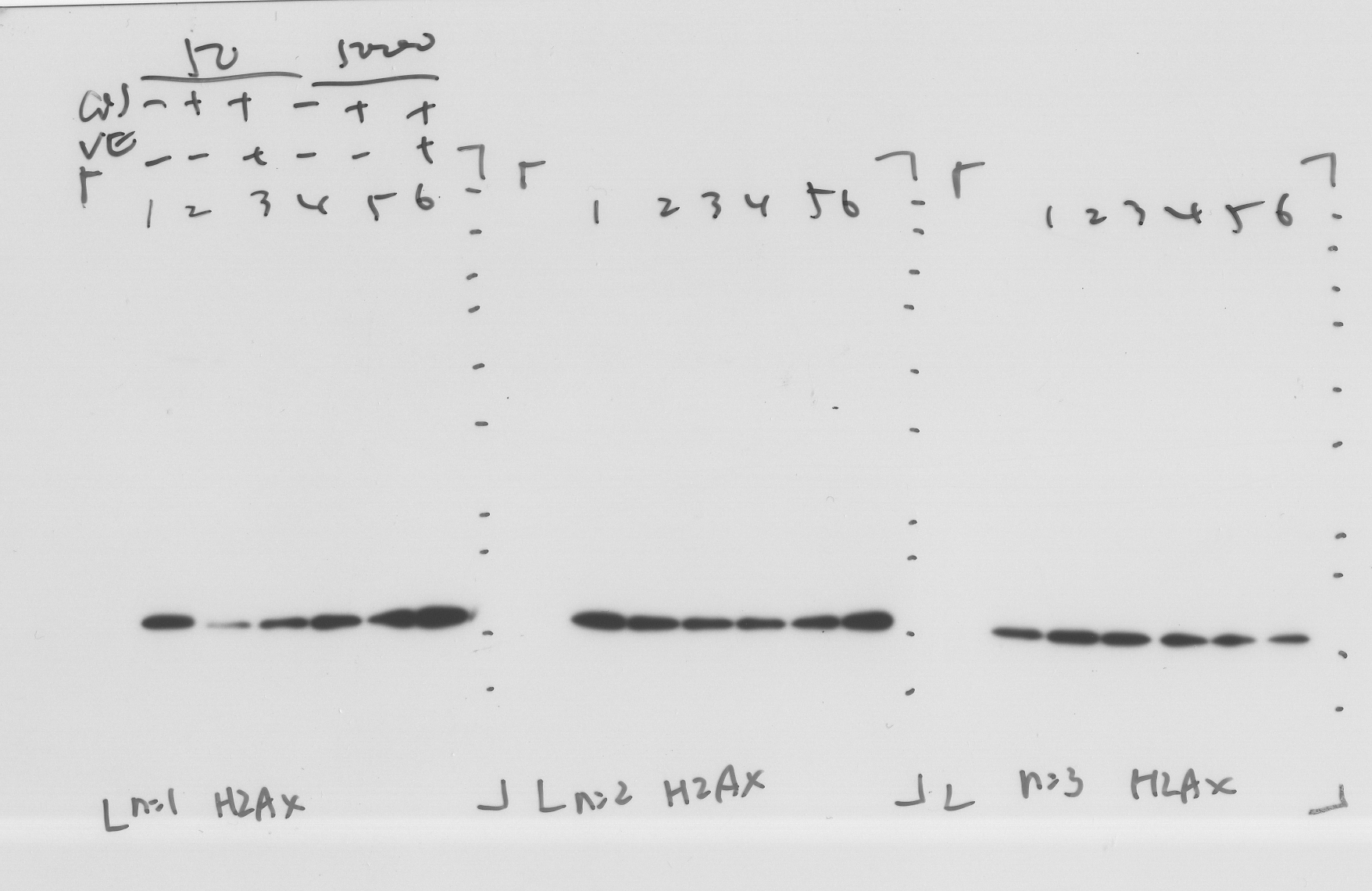

Supplement: Figure 4—source data 2. [file elife-83870-fig4-data2.zip › Figure 4-source data 2/Figure 4-source data 2 (H2AX).tif]

Figure 4-source data 3

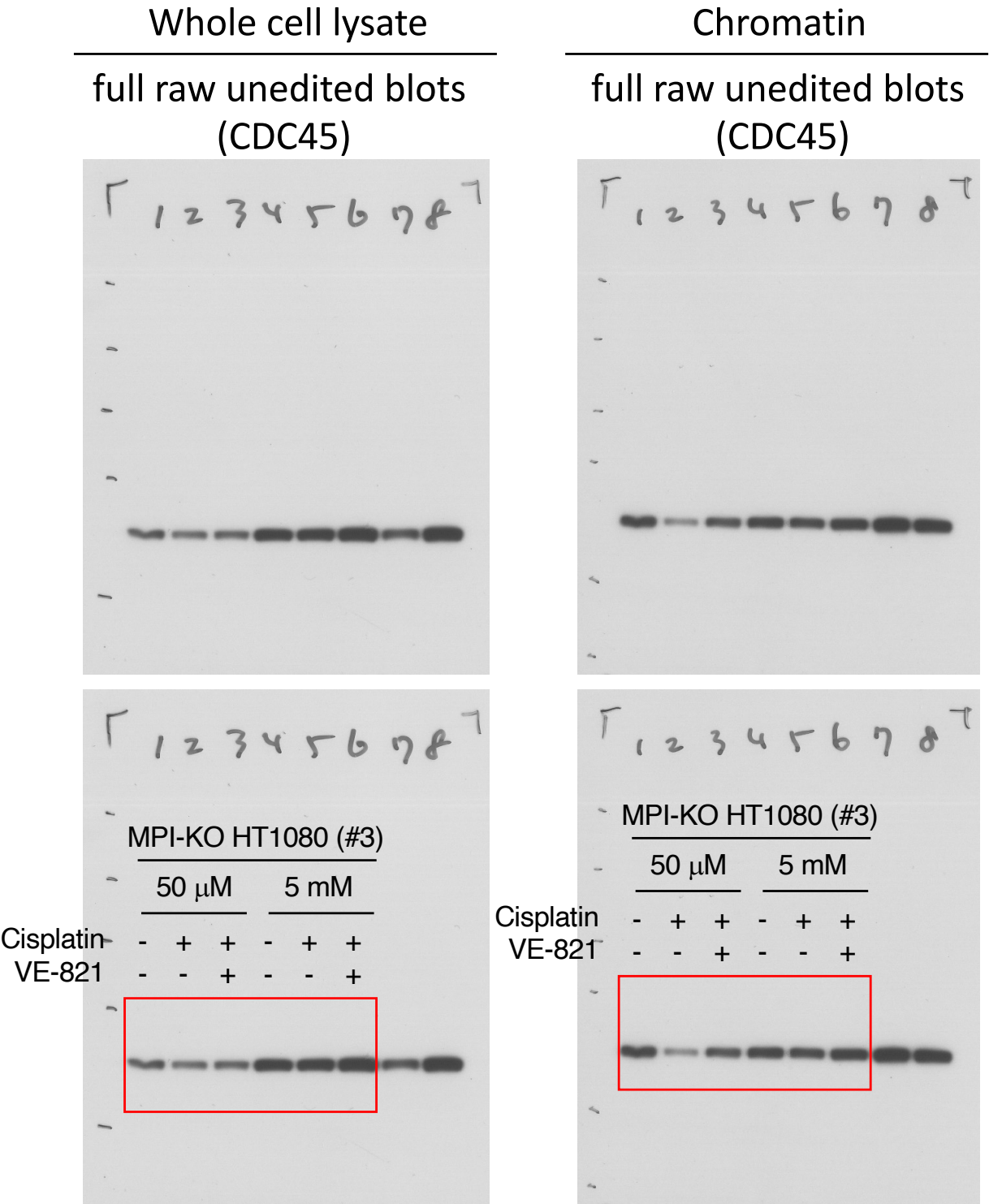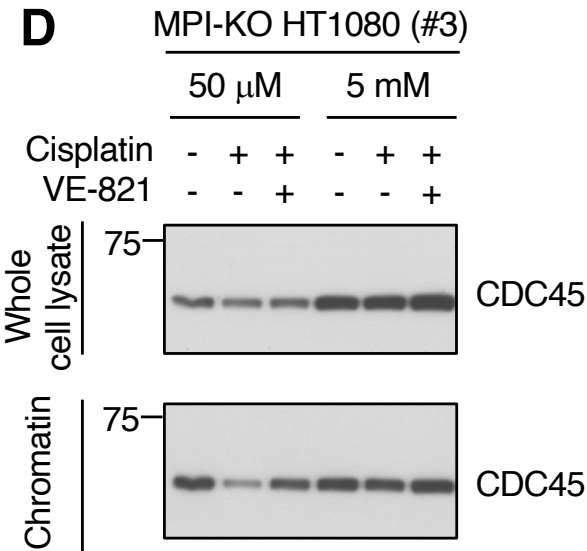

Supplement: Figure 4—source data 3. [file elife-83870-fig4-data3.zip › Figure 4-source data 3/Figure 4-source data 3.pdf]

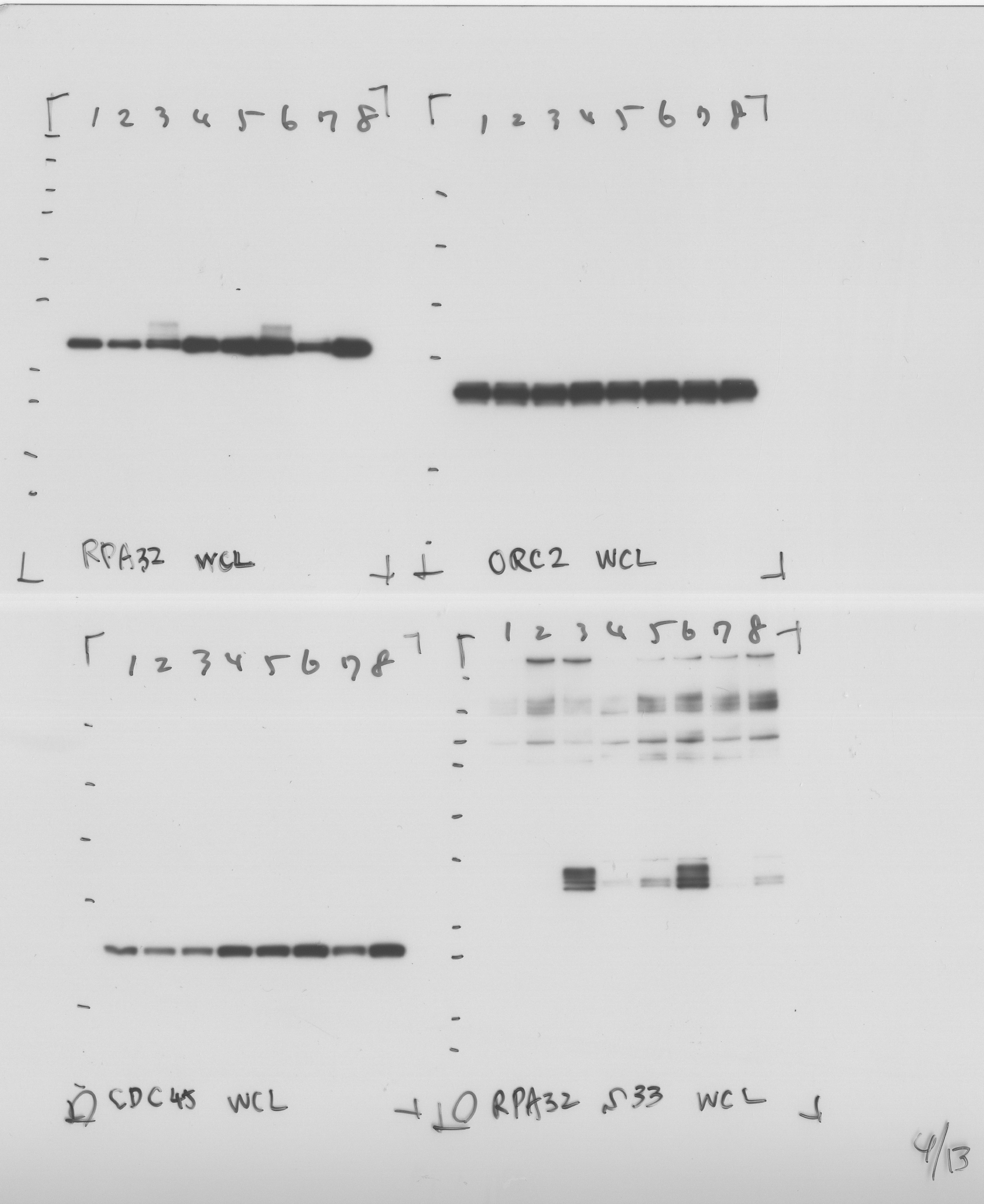

Supplement: Figure 4—source data 3. [file elife-83870-fig4-data3.zip › Figure 4-source data 3/Figure 4-source data 3 (CDC45_WCL).tif]

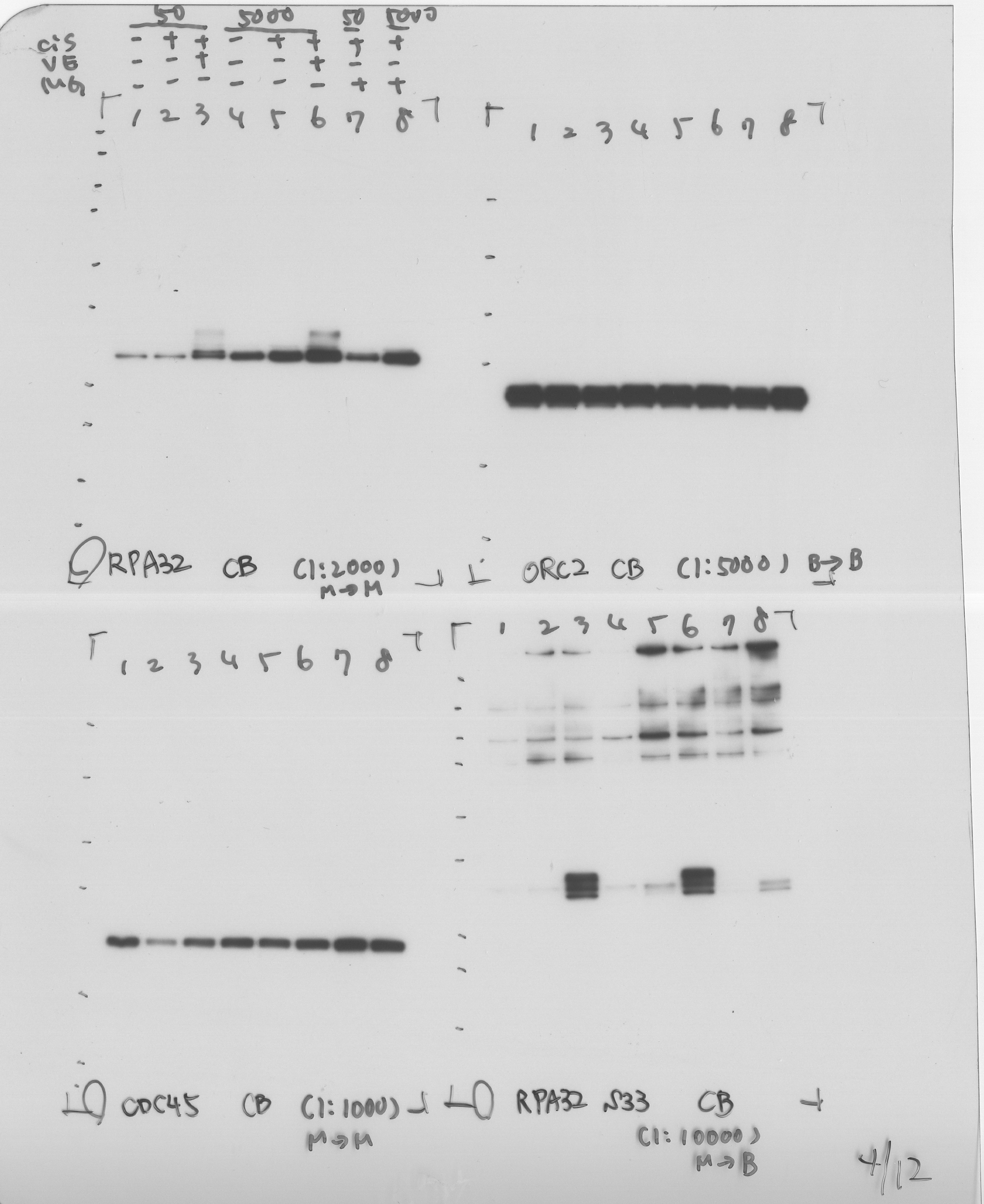

Supplement: Figure 4—source data 3. [file elife-83870-fig4-data3.zip › Figure 4-source data 3/Figure 4-source data 3 (CDC45_chromatin).tif]

Figure 4-source data 4

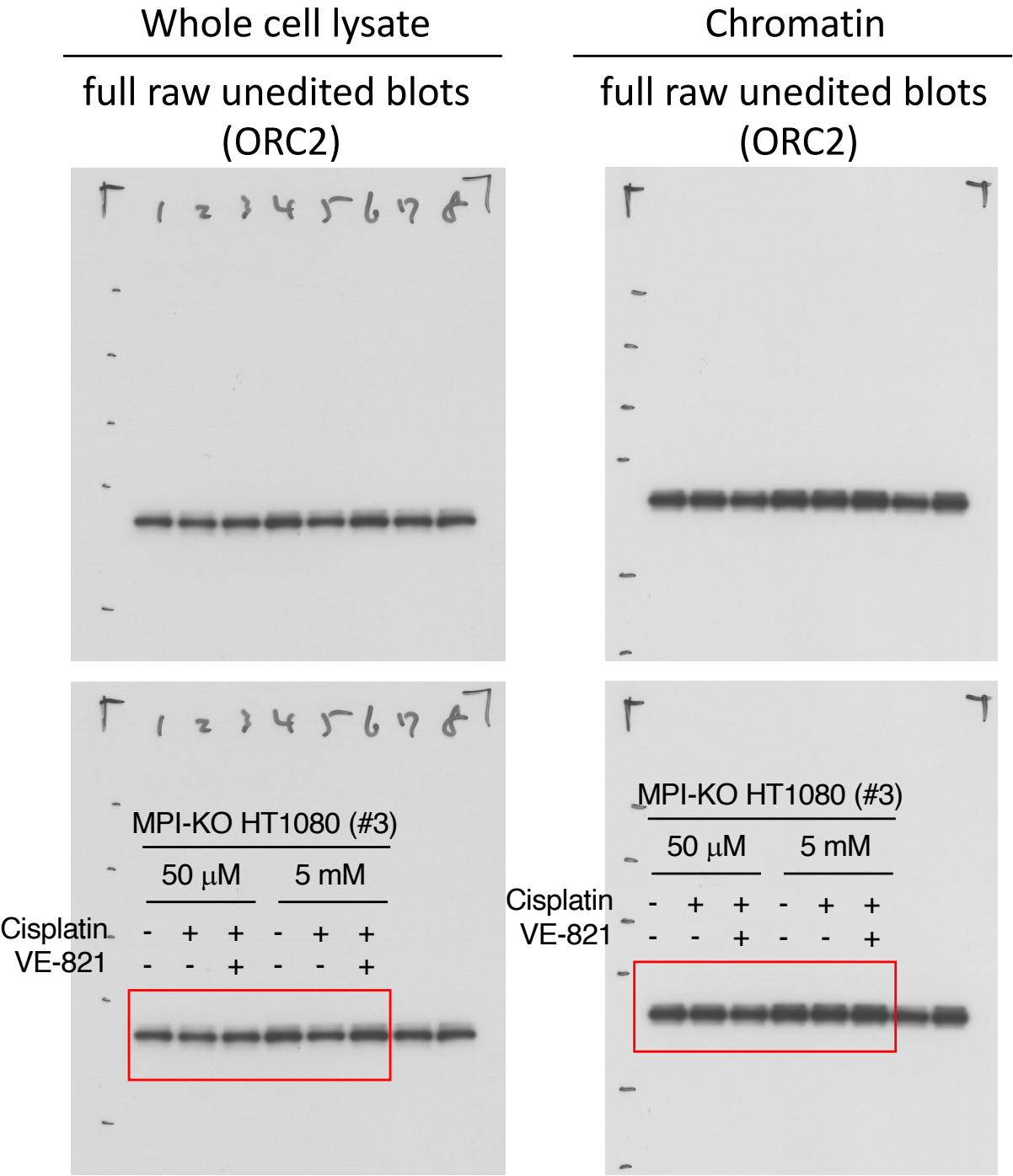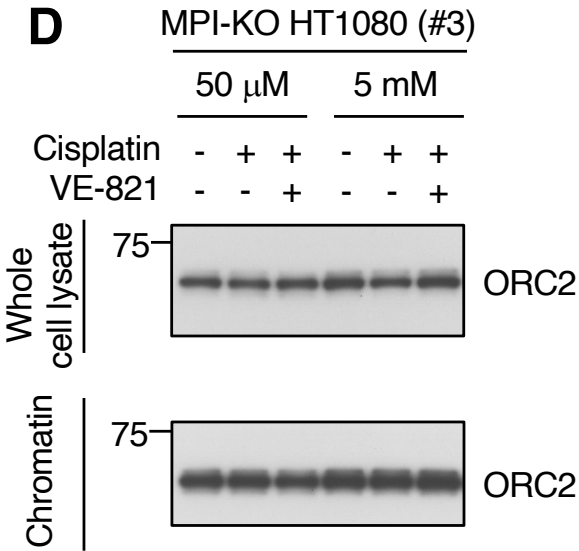

Supplement: Figure 4—source data 4. [file elife-83870-fig4-data4.zip › Figure 4-source data 4/Figure 4-source data 4.pdf]

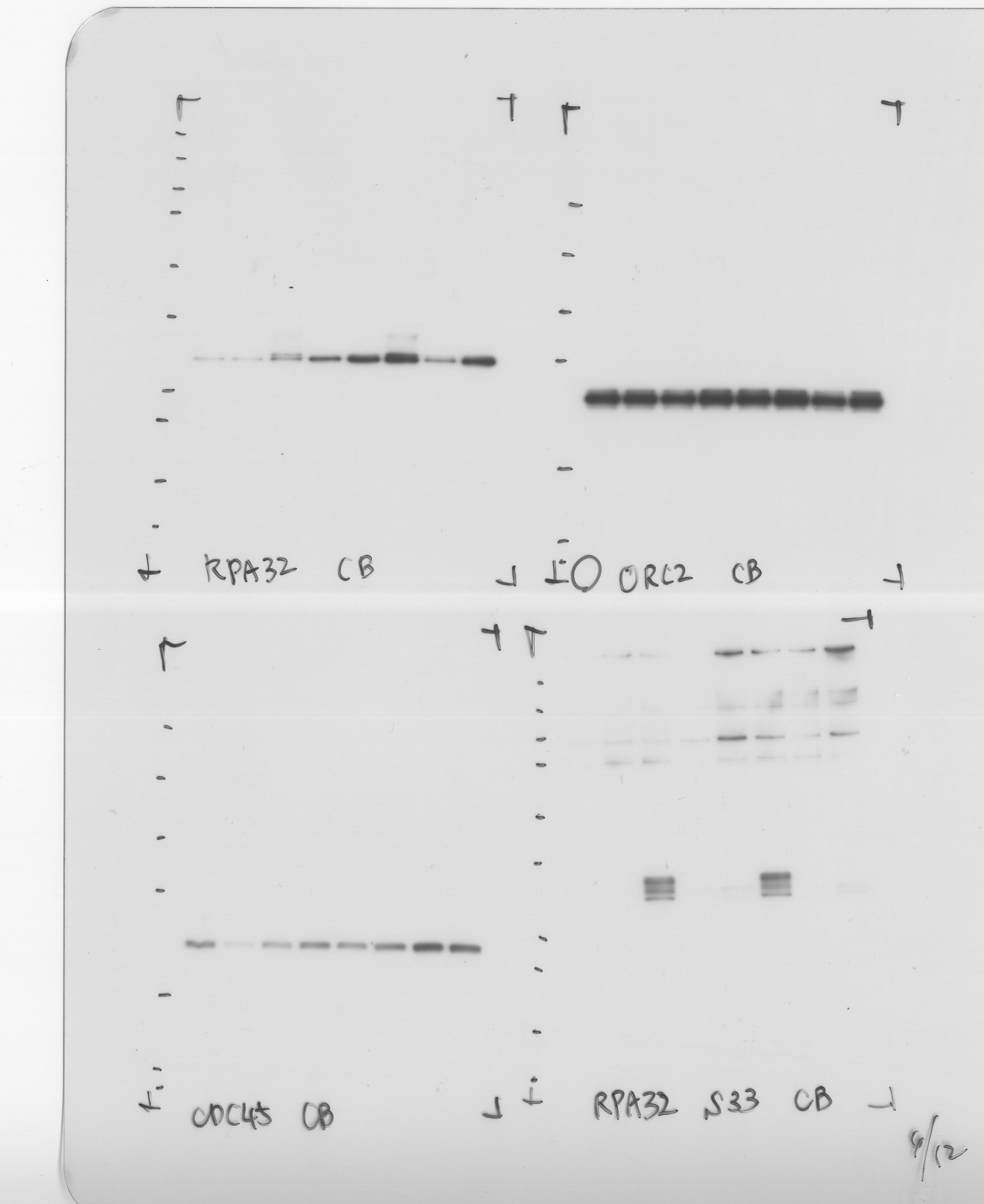

Supplement: Figure 4—source data 4. [file elife-83870-fig4-data4.zip › Figure 4-source data 4/Figure 4-source data 4 (ORC2_chromatin).tif]

Figure XX-figure supplement YY-source data 1

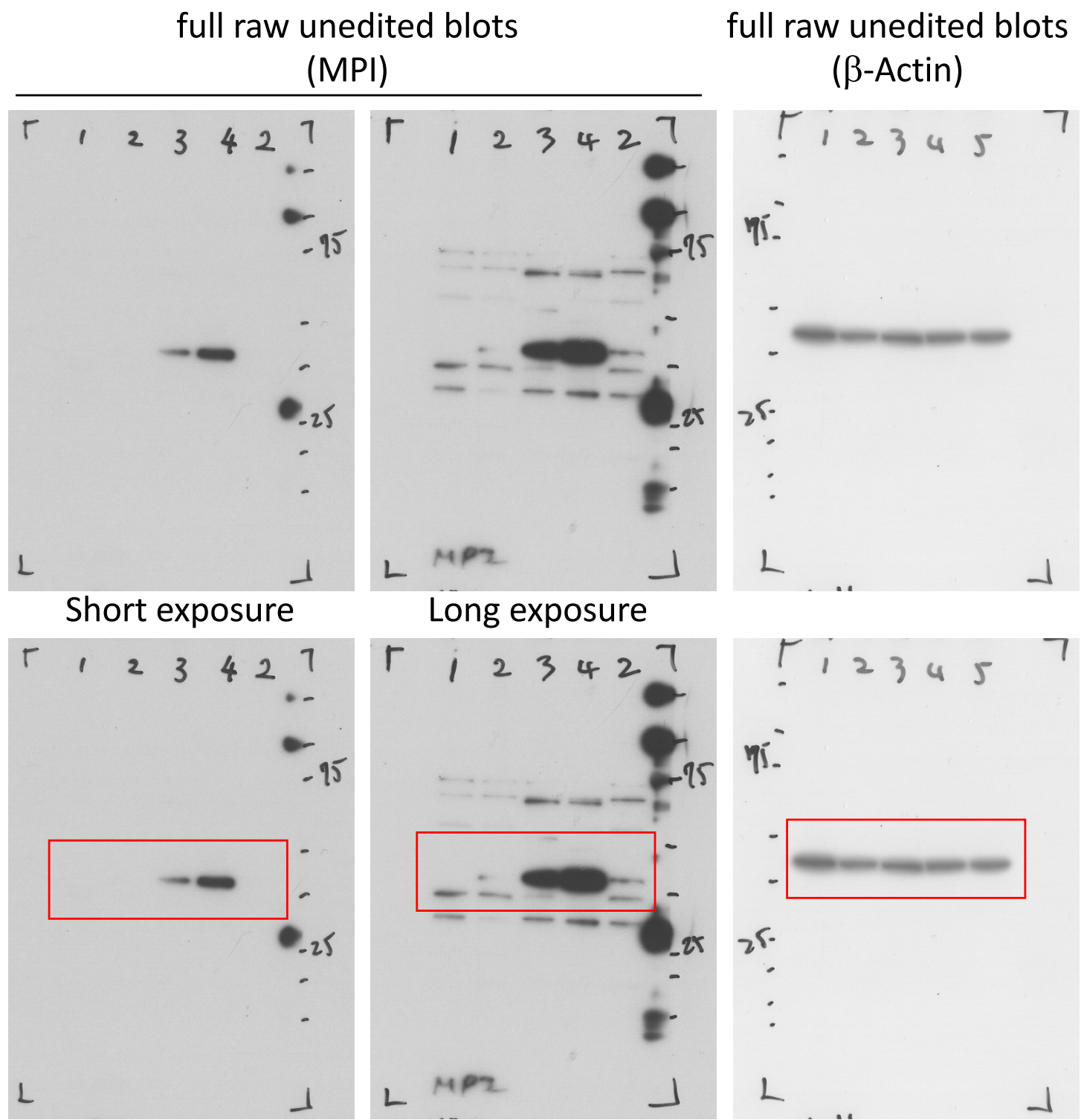

**A**

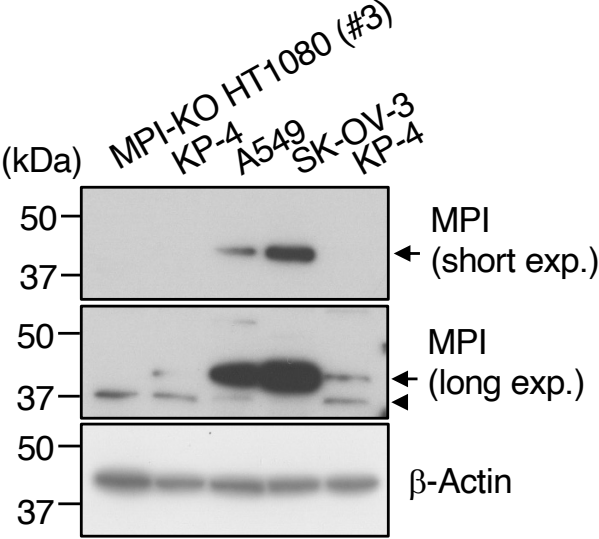

Supplement: Figure 4—figure supplement 3—source data 1. [file elife-83870-fig4-figsupp3-data1.zip › Figure 4-figure supplement 3-source data 1/Figure 4-figure supplement 3-source data 1.pdf]

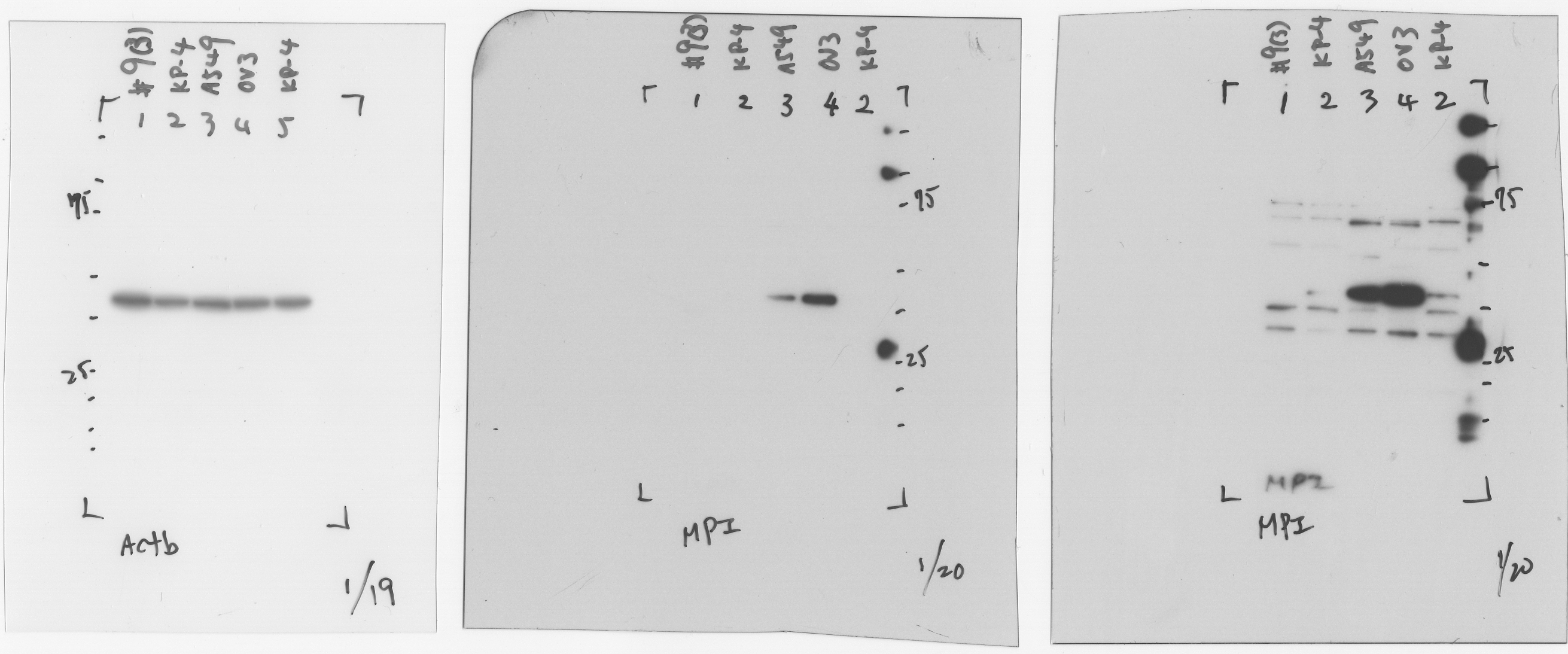

Supplement: Figure 4—figure supplement 3—source data 1. [file elife-83870-fig4-figsupp3-data1.zip › Figure 4-figure supplement 3-source data 1/Figure 4-figure supplement 3-source data 1.tif]

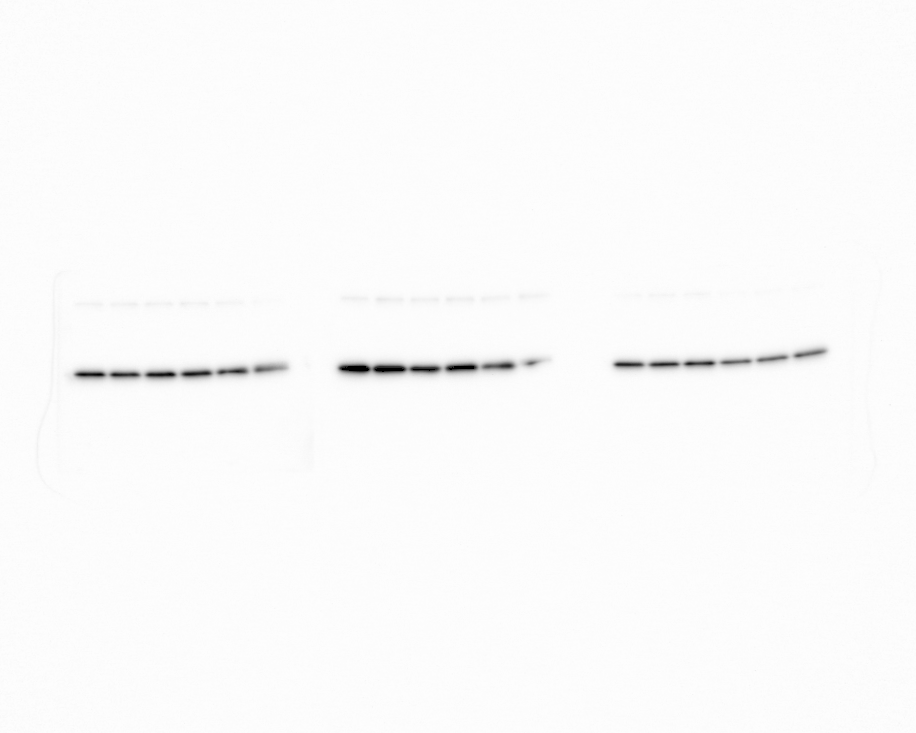

Supplement: Figure 4—figure supplement 3—source data 2. [file elife-83870-fig4-figsupp3-data2.zip › Figure 4-figure supplement 3-source data 2/Figure 4-figure supplement 3-source data 2 (H2AX).tif]

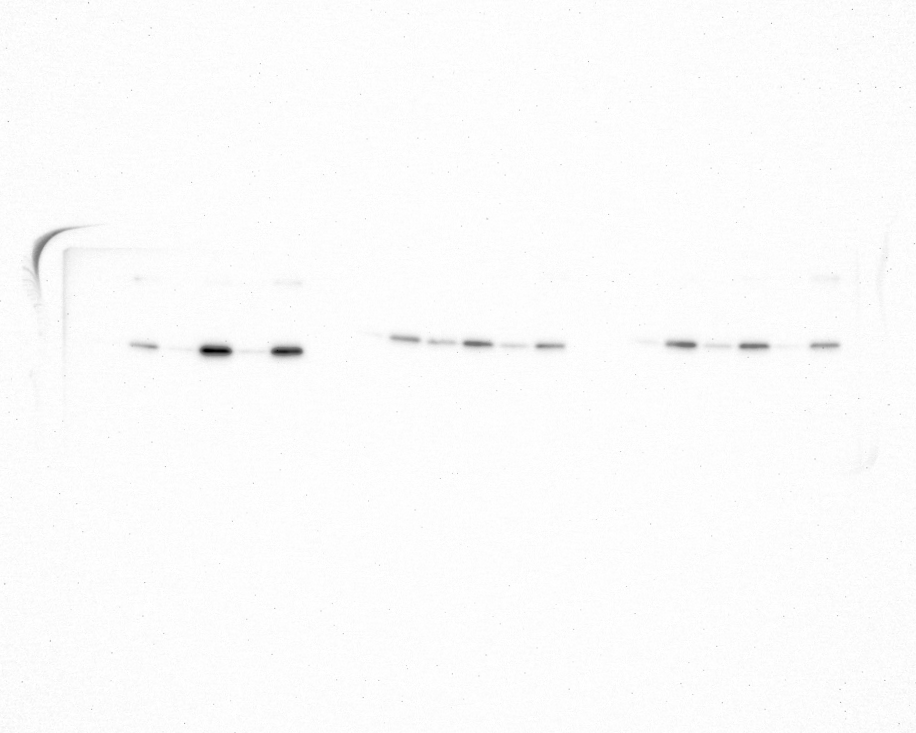

Supplement: Figure 4—figure supplement 3—source data 2. [file elife-83870-fig4-figsupp3-data2.zip › Figure 4-figure supplement 3-source data 2/Figure 4-figure supplement 3-source data 2 (gH2AX).tif]

Figure 1-source data 1

full raw unedited blots ( $\beta$ -Actin)

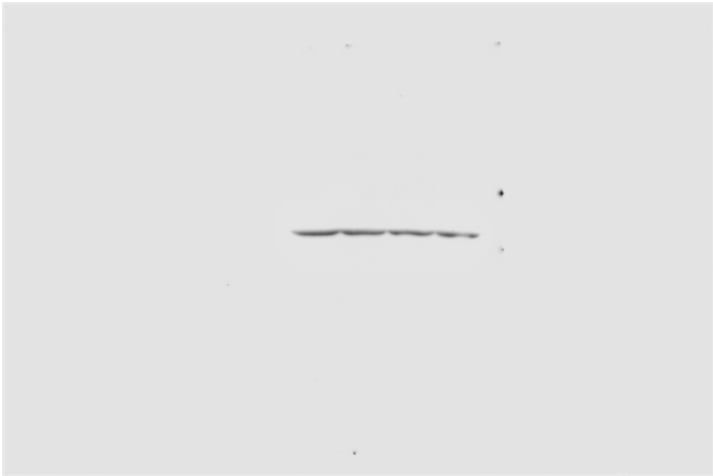

full raw unedited blots (MPI)

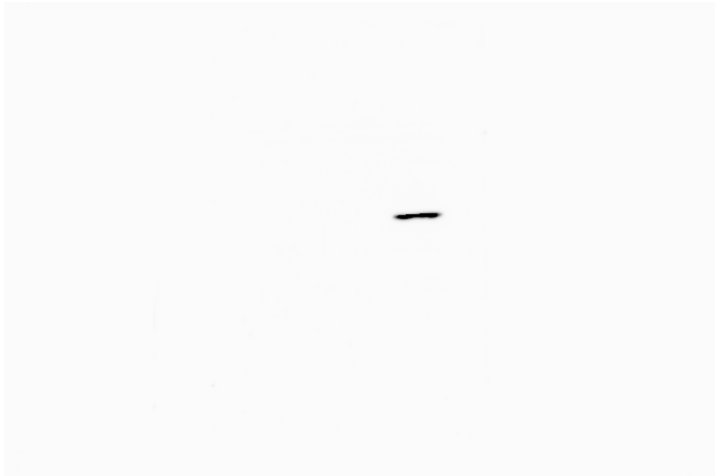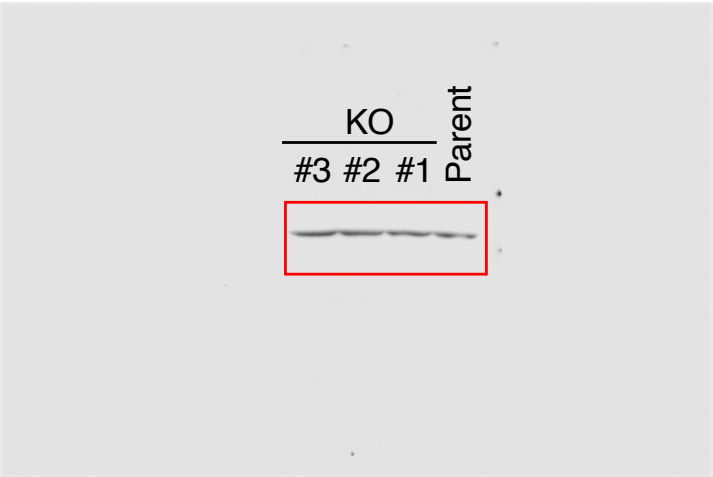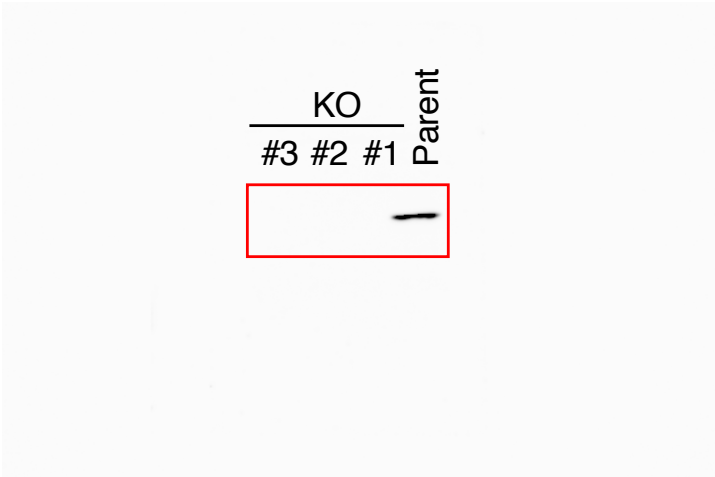

**A**

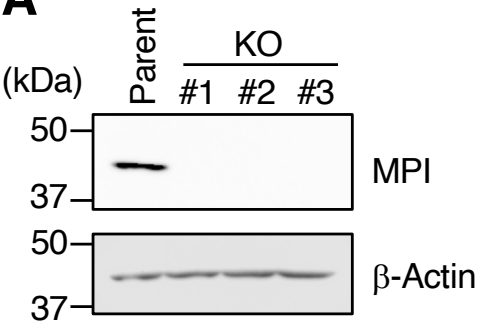

Supplement: Figure 4—figure supplement 3—source data 2. [file elife-83870-fig4-figsupp3-data2.zip › Figure 4-figure supplement 3-source data 2/Figure 4-figure supplement 3-source data 2.pdf]

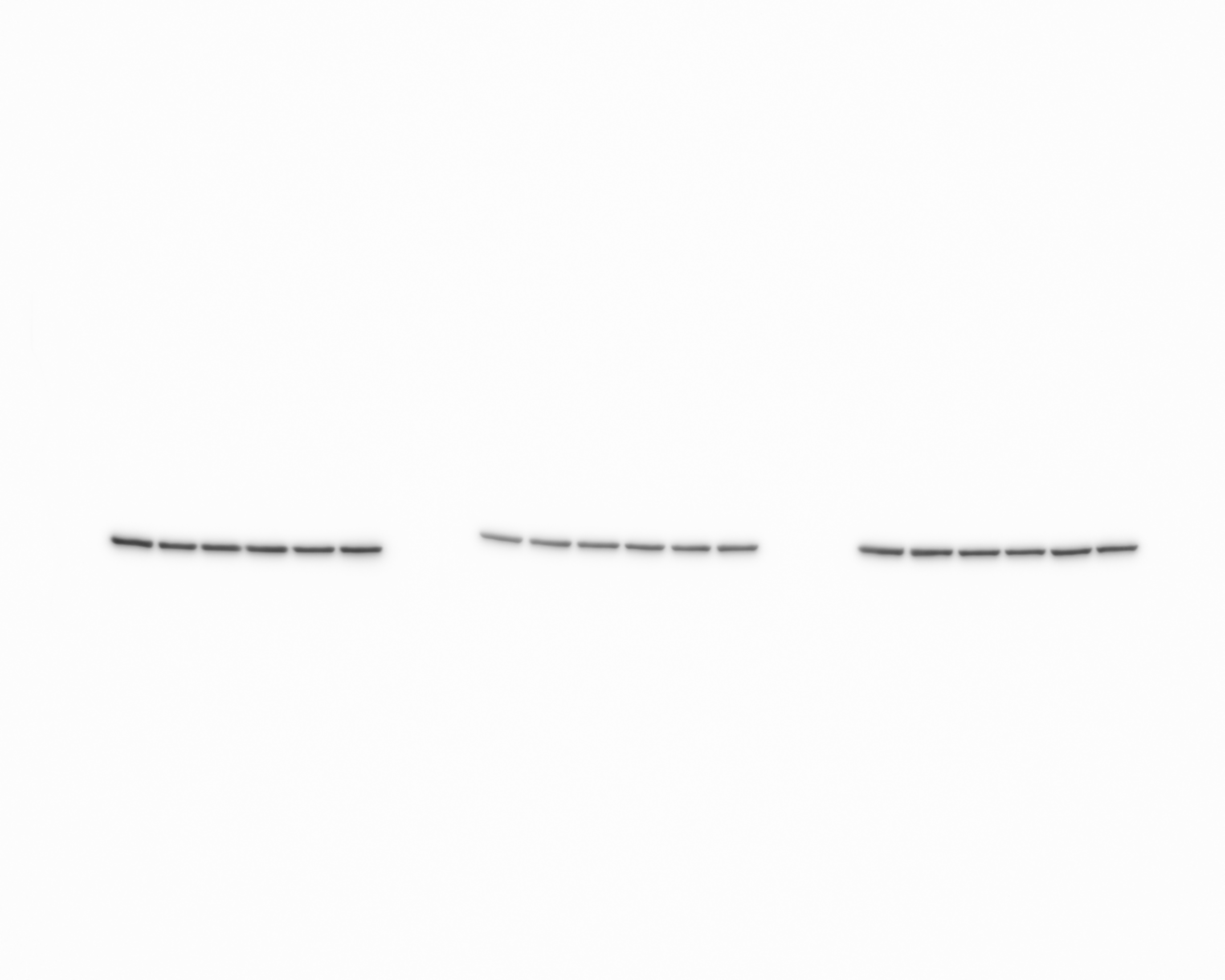

Supplement: Figure 4—figure supplement 3—source data 2. [file elife-83870-fig4-figsupp3-data2.zip › Figure 4-figure supplement 3-source data 2/Figure 4-figure supplement 3-source data 2 (Actb).tif]

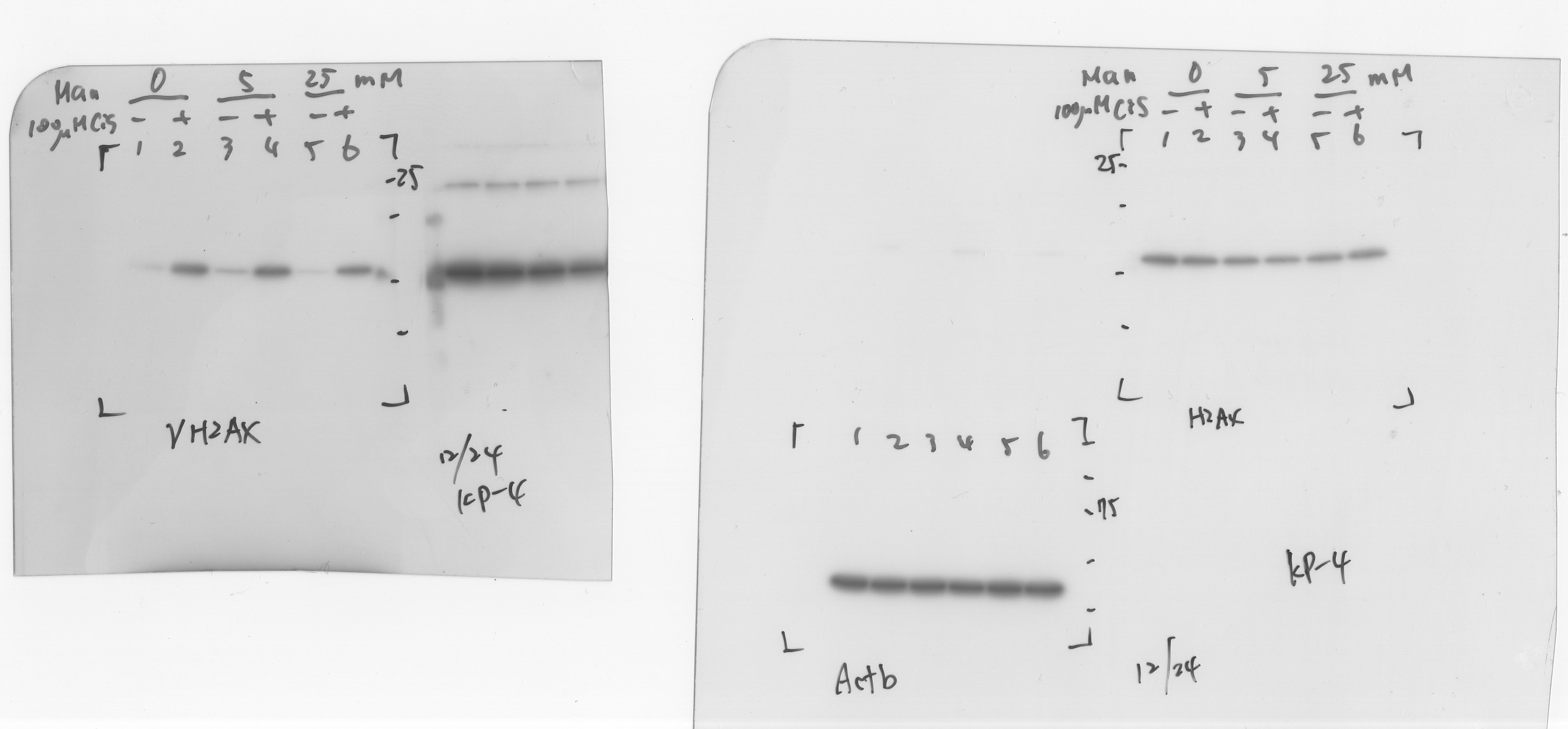

Supplement: Figure 4—figure supplement 3—source data 3. [file elife-83870-fig4-figsupp3-data3.zip › Figure 4-figure supplement 3-source data 3/Figure 4-figure supplement 3-source data 3 (KP-4).tif]

Figure 1-source data 1

full raw unedited blots ( $\beta$ -Actin)

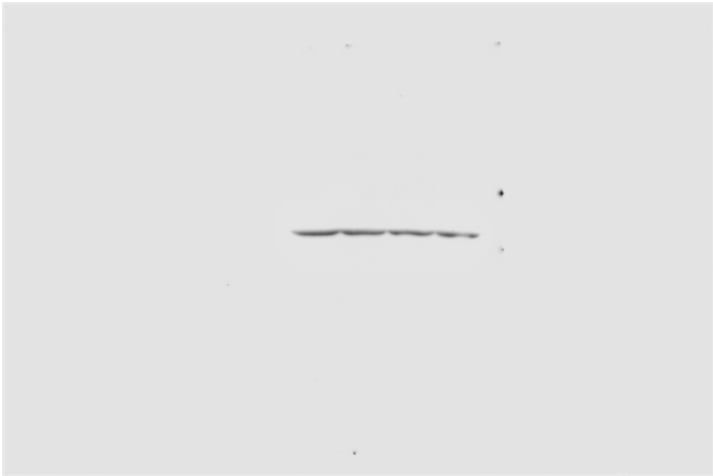

full raw unedited blots (MPI)

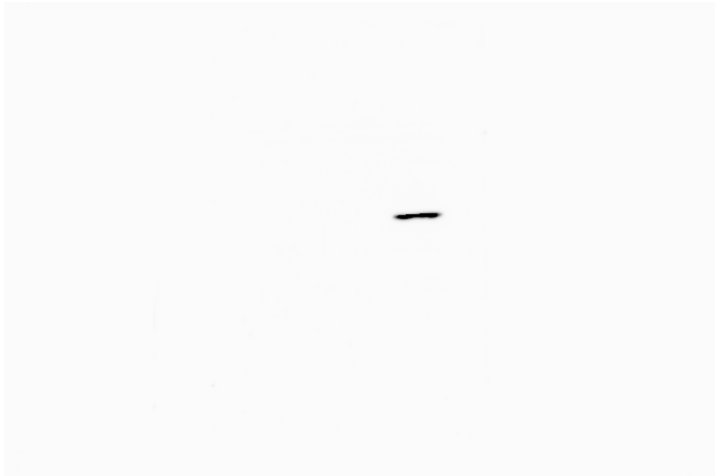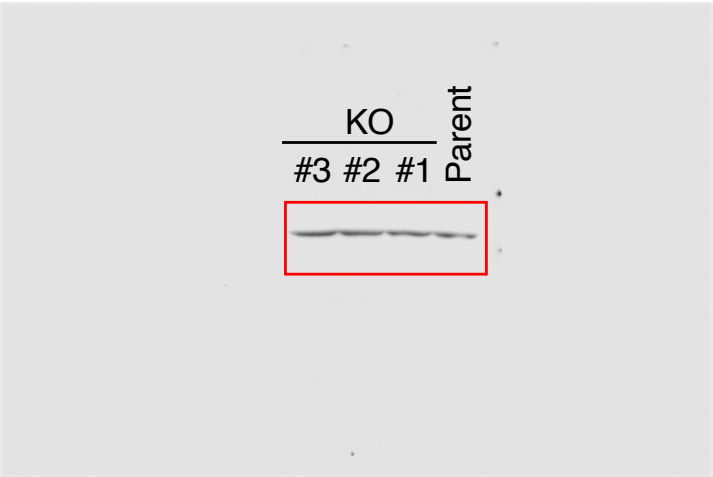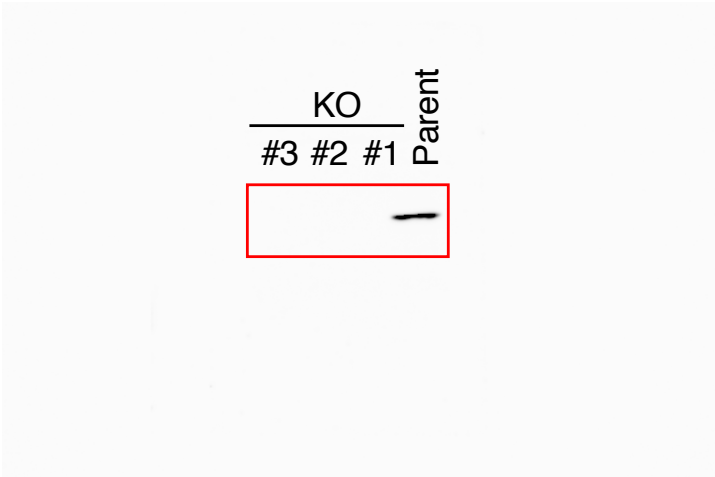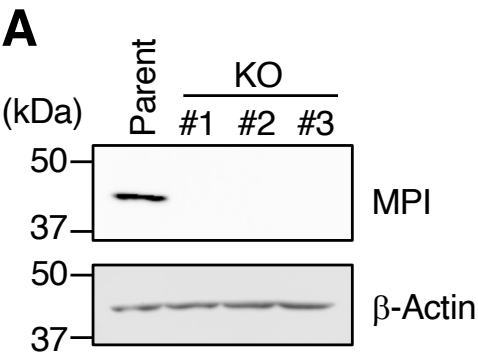

Supplement: Figure 4—figure supplement 3—source data 3. [file elife-83870-fig4-figsupp3-data3.zip › Figure 4-figure supplement 3-source data 3/Figure 4-figure supplement 3-source data 3.pdf]

Figure 1-source data 1

full raw unedited blots ( $\beta$ -Actin)

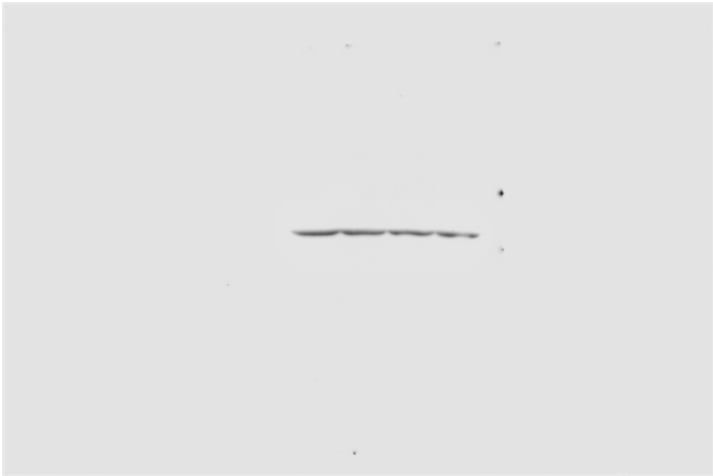

full raw unedited blots (MPI)

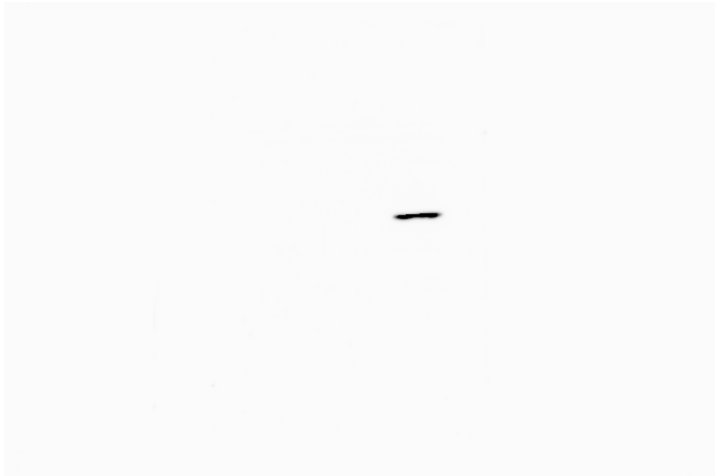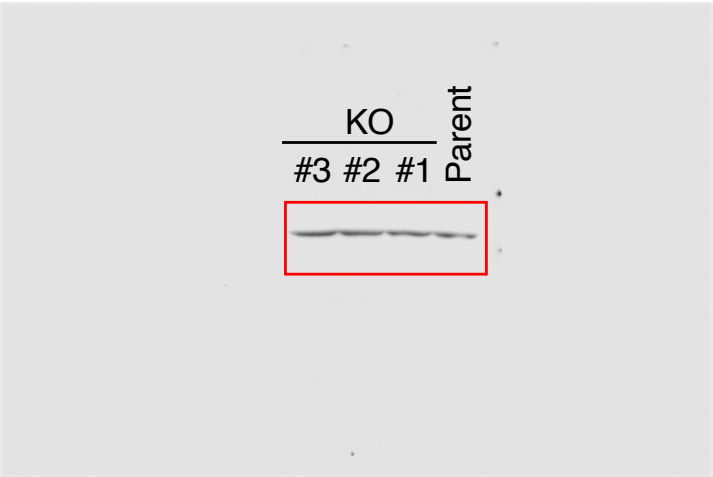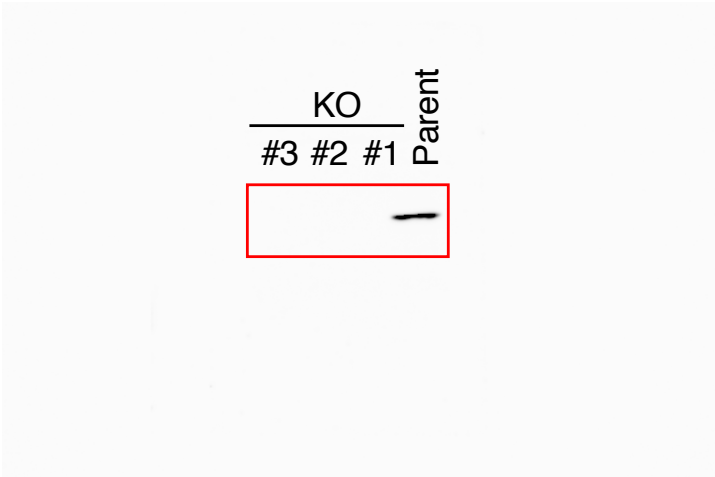

**A**

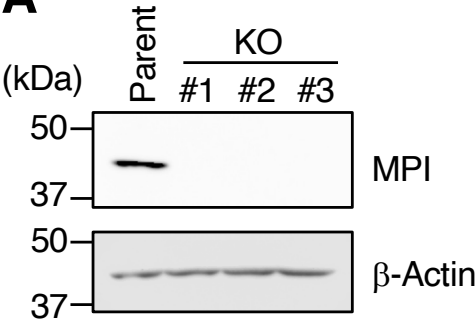

Supplement: Figure 4—figure supplement 3—source data 4. [file elife-83870-fig4-figsupp3-data4.zip › Figure 4-figure supplement 3-source data 4/Figure 4-figure supplement 3-source data 4.pdf]

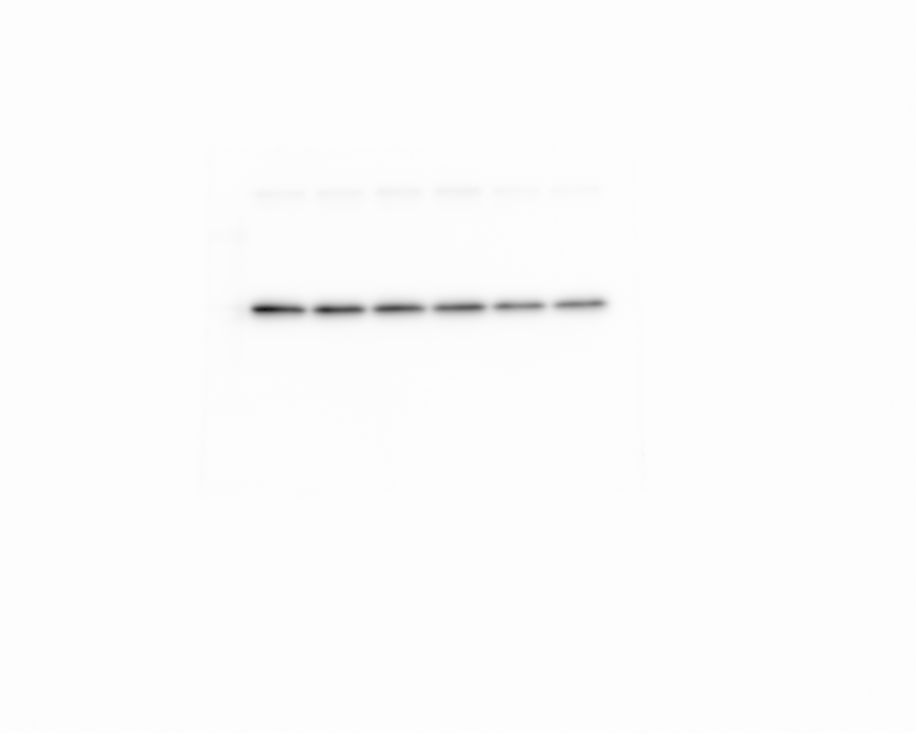

Supplement: Figure 4—figure supplement 3—source data 5. [file elife-83870-fig4-figsupp3-data5.zip › Figure 4-figure supplement 3-source data 5/Figure 4-figure supplement 3-source data 2 (H2AX_SKOV3).tif]

Figure 1-source data 1

full raw unedited blots ( $\beta$ -Actin)

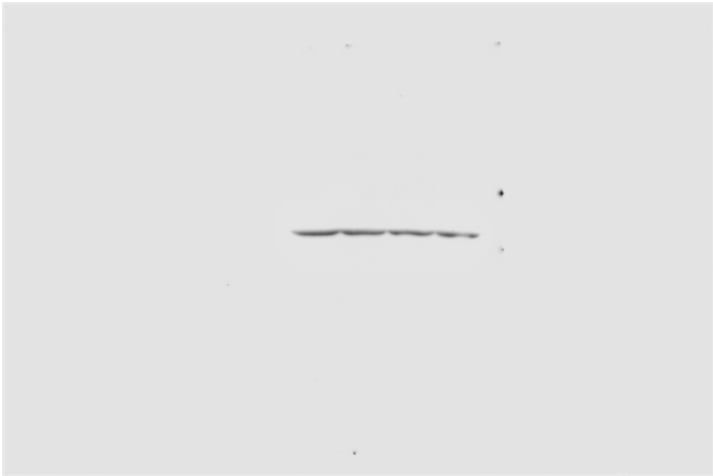

full raw unedited blots (MPI)

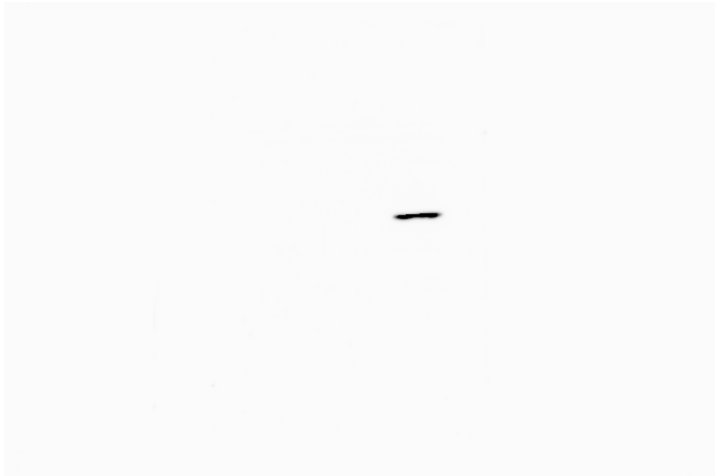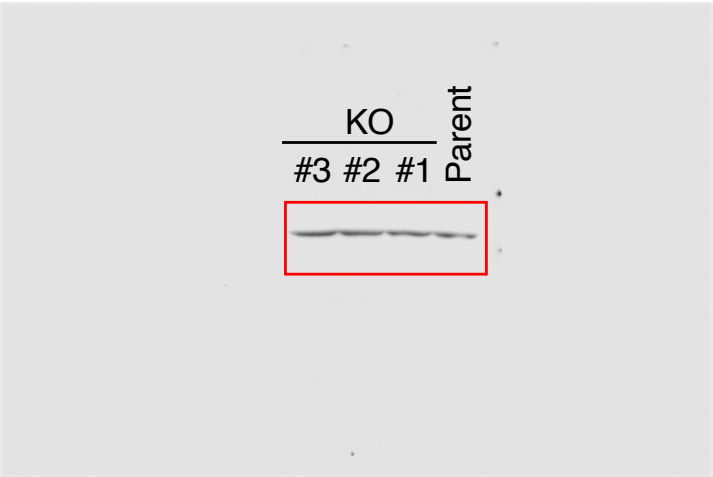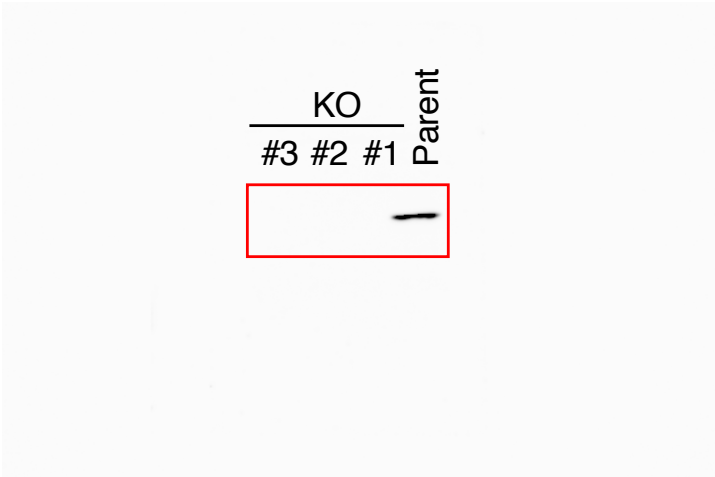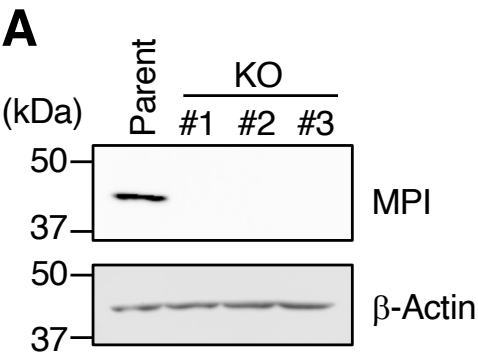

Supplement: Figure 4—figure supplement 3—source data 5. [file elife-83870-fig4-figsupp3-data5.zip › Figure 4-figure supplement 3-source data 5/Figure 4-figure supplement 3-source data 5.pdf]

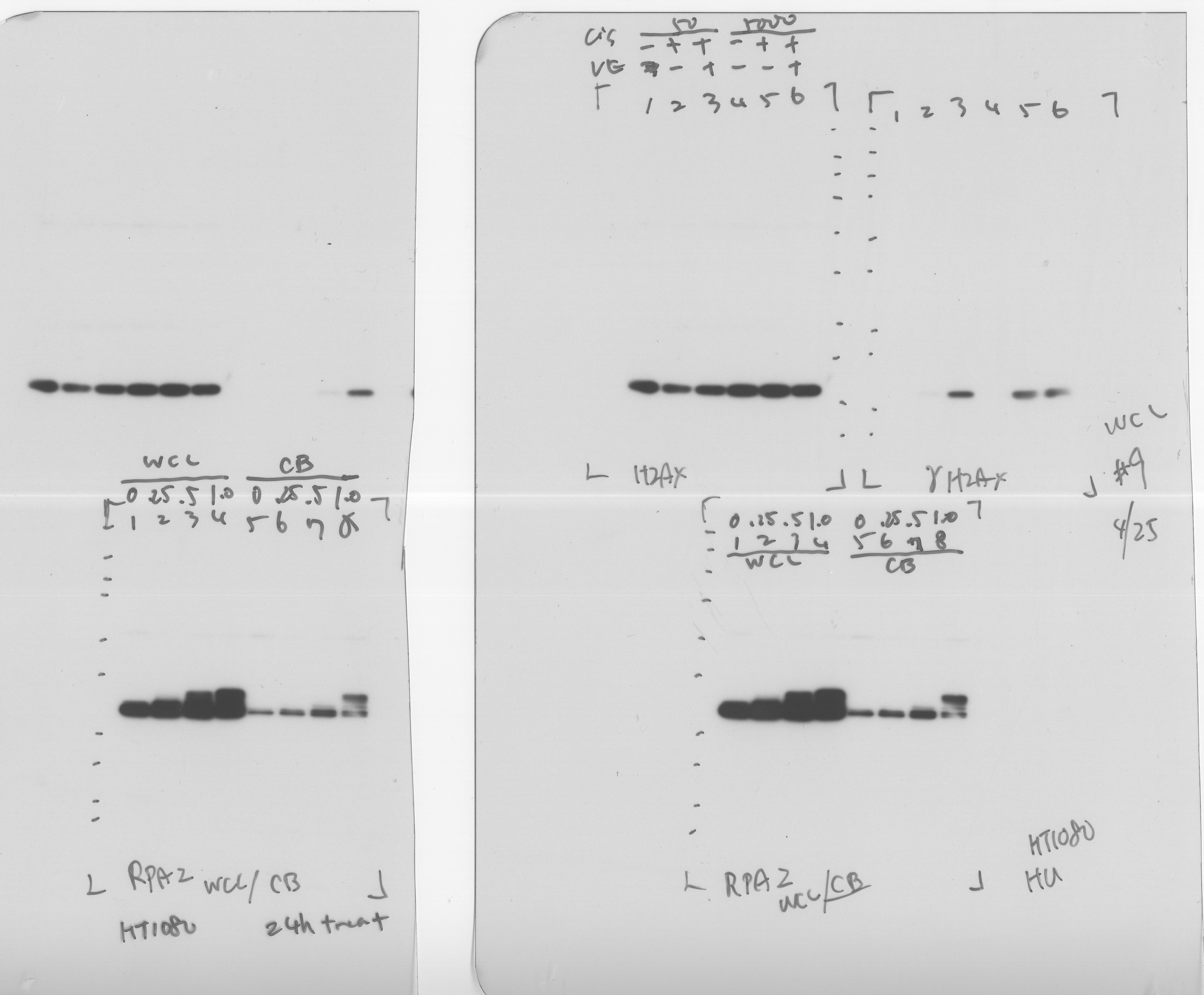

Supplement: Figure 7—source data 1. [file elife-83870-fig7-data1.zip › Figure 7-source data 1/Figure 7-source data 1 (H2AX).tif]

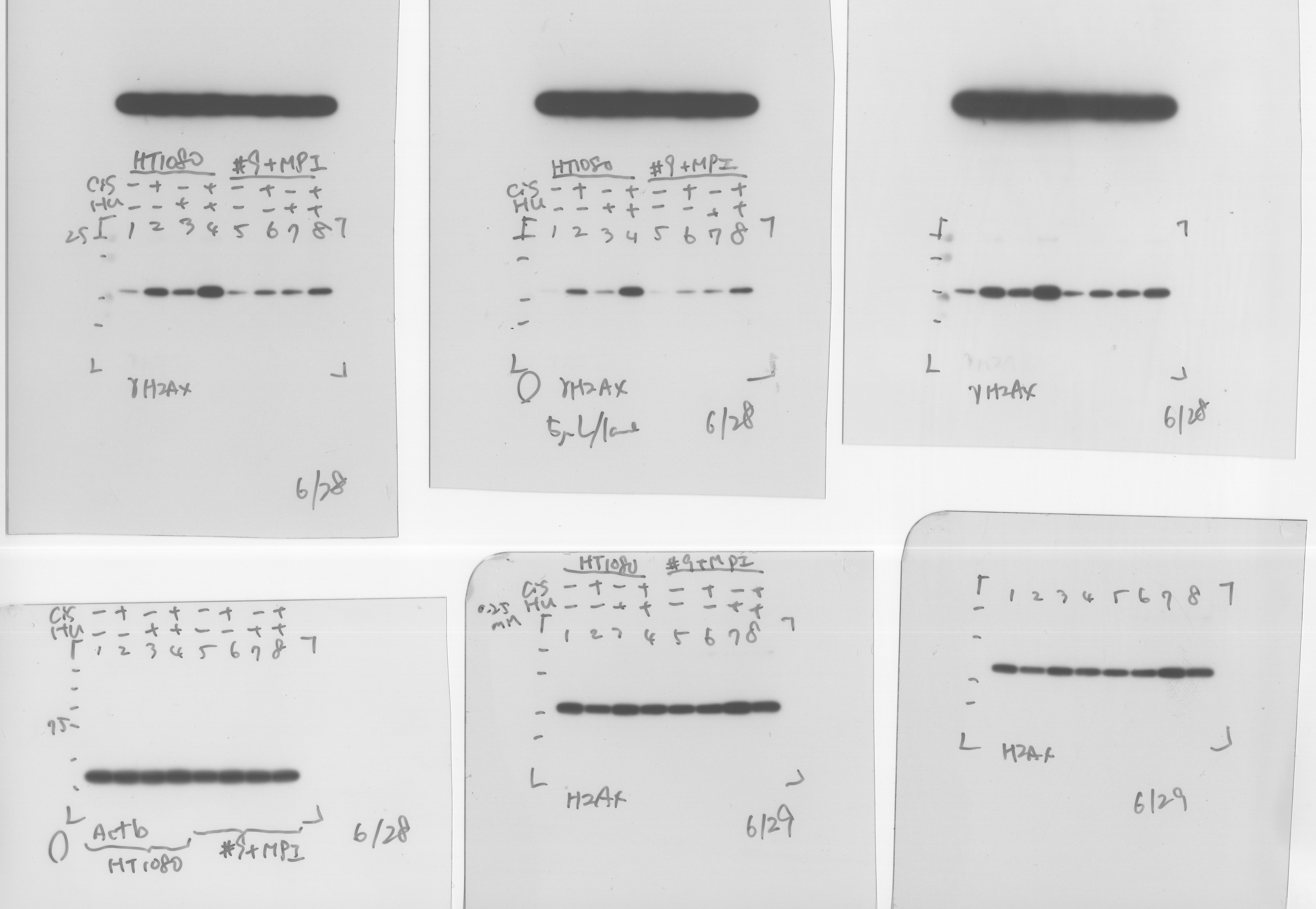

Supplement: Figure 7—source data 1. [file elife-83870-fig7-data1.zip › Figure 7-source data 1/Figure 7-source data 1 (gammaH2AX_ACTB).tif]

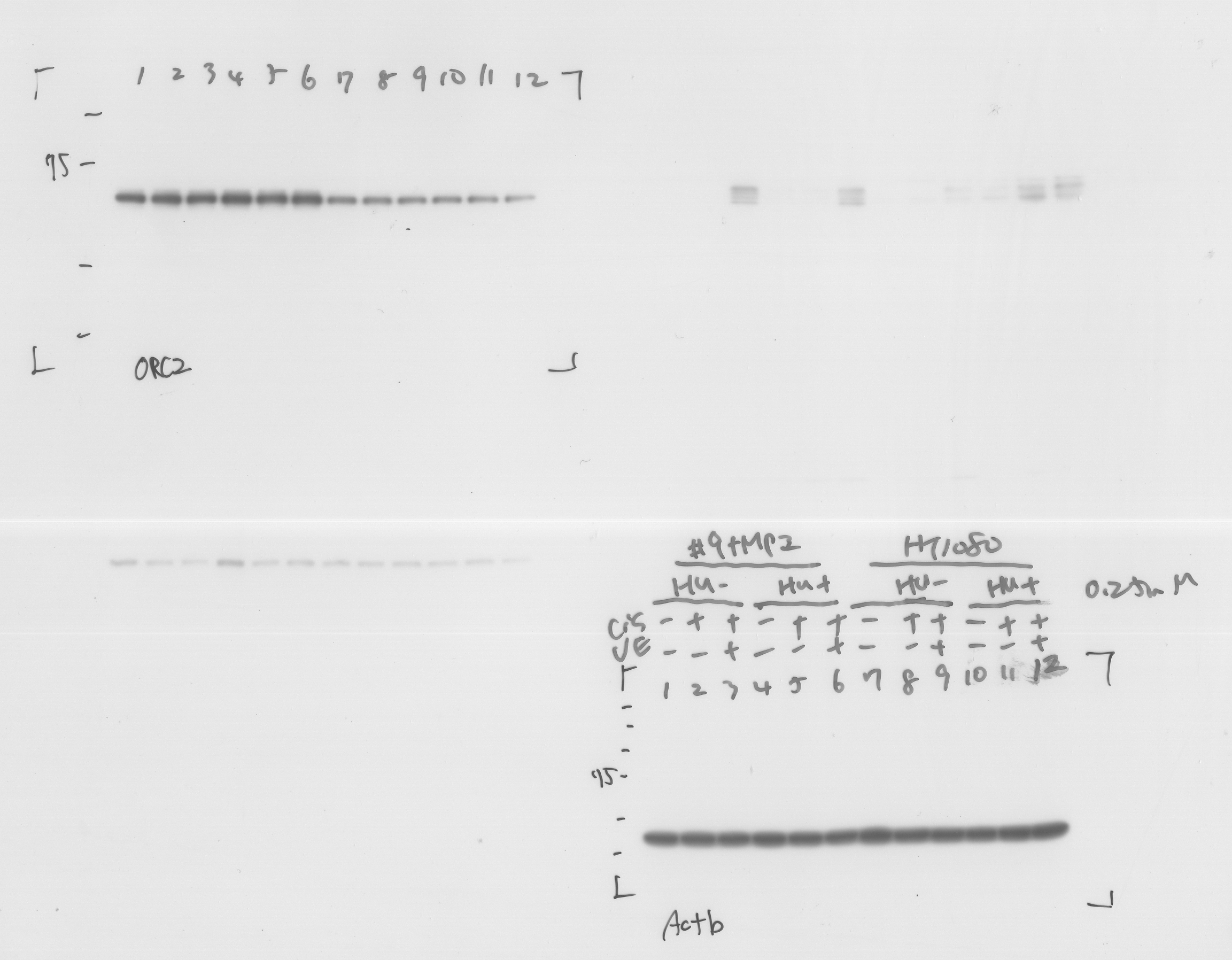

Supplement: Figure 7—source data 2. [file elife-83870-fig7-data2.zip › Figure 7-source data 2/Figure 7-source data 2 (ACTB).tif]

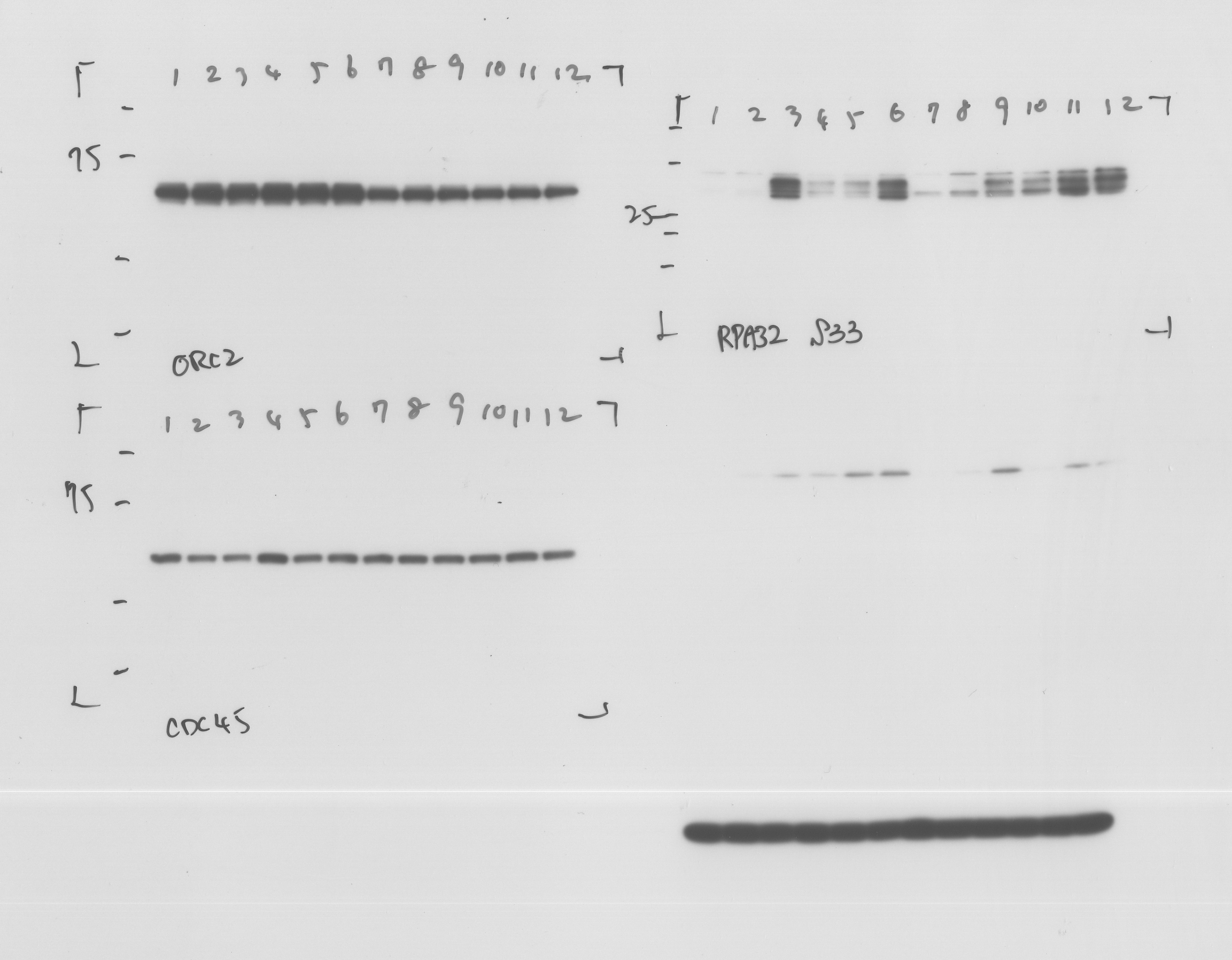

Supplement: Figure 7—source data 2. [file elife-83870-fig7-data2.zip › Figure 7-source data 2/Figure 7-source data 2 (pRPA2 (S33)).tif]

Figure 7-source data 2

Whole cell lysate

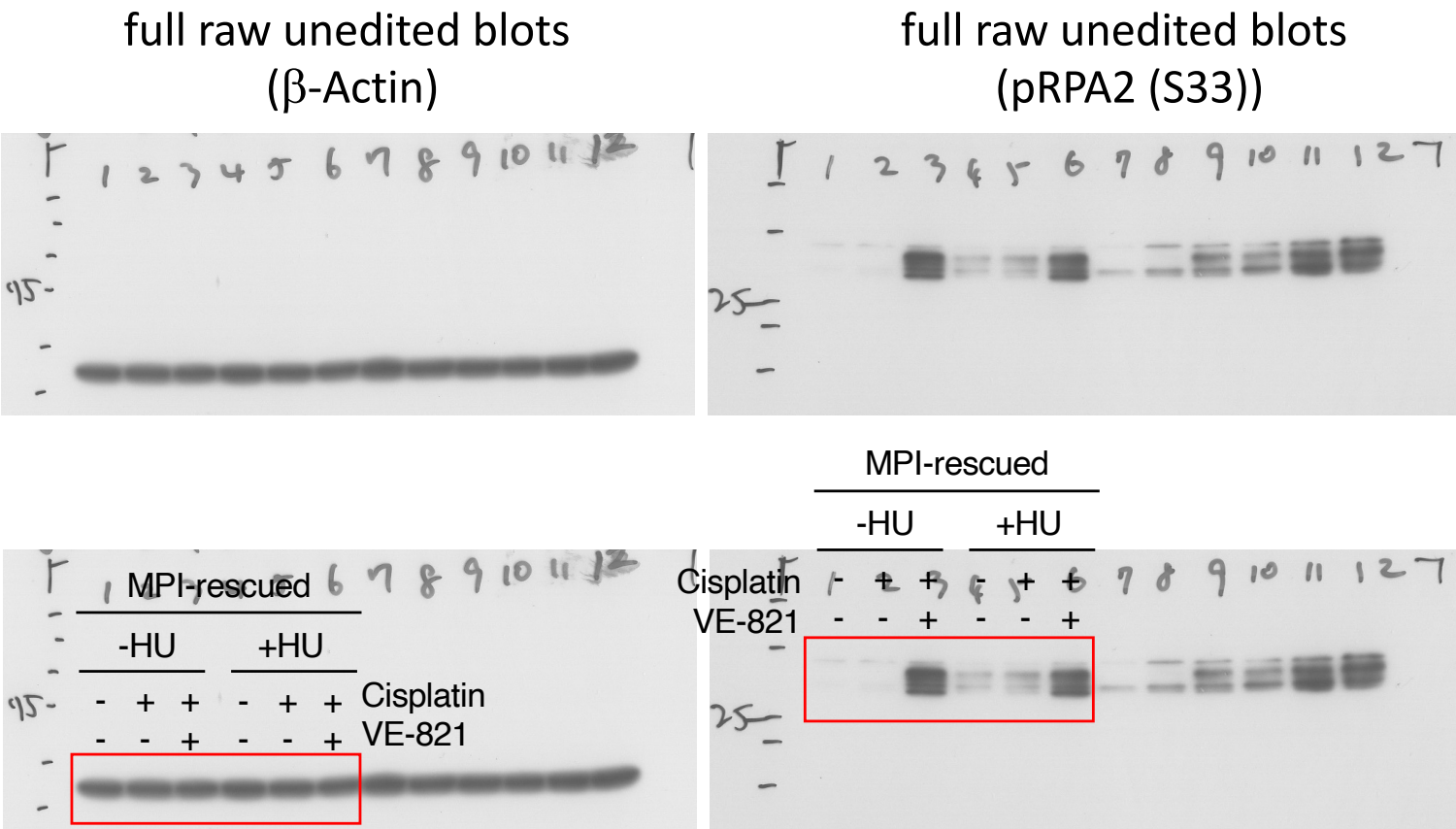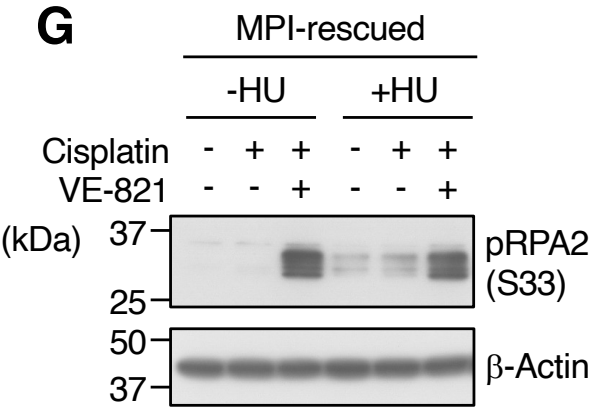

Supplement: Figure 7—source data 2. [file elife-83870-fig7-data2.zip › Figure 7-source data 2/Figure 7-source data 2.pdf]

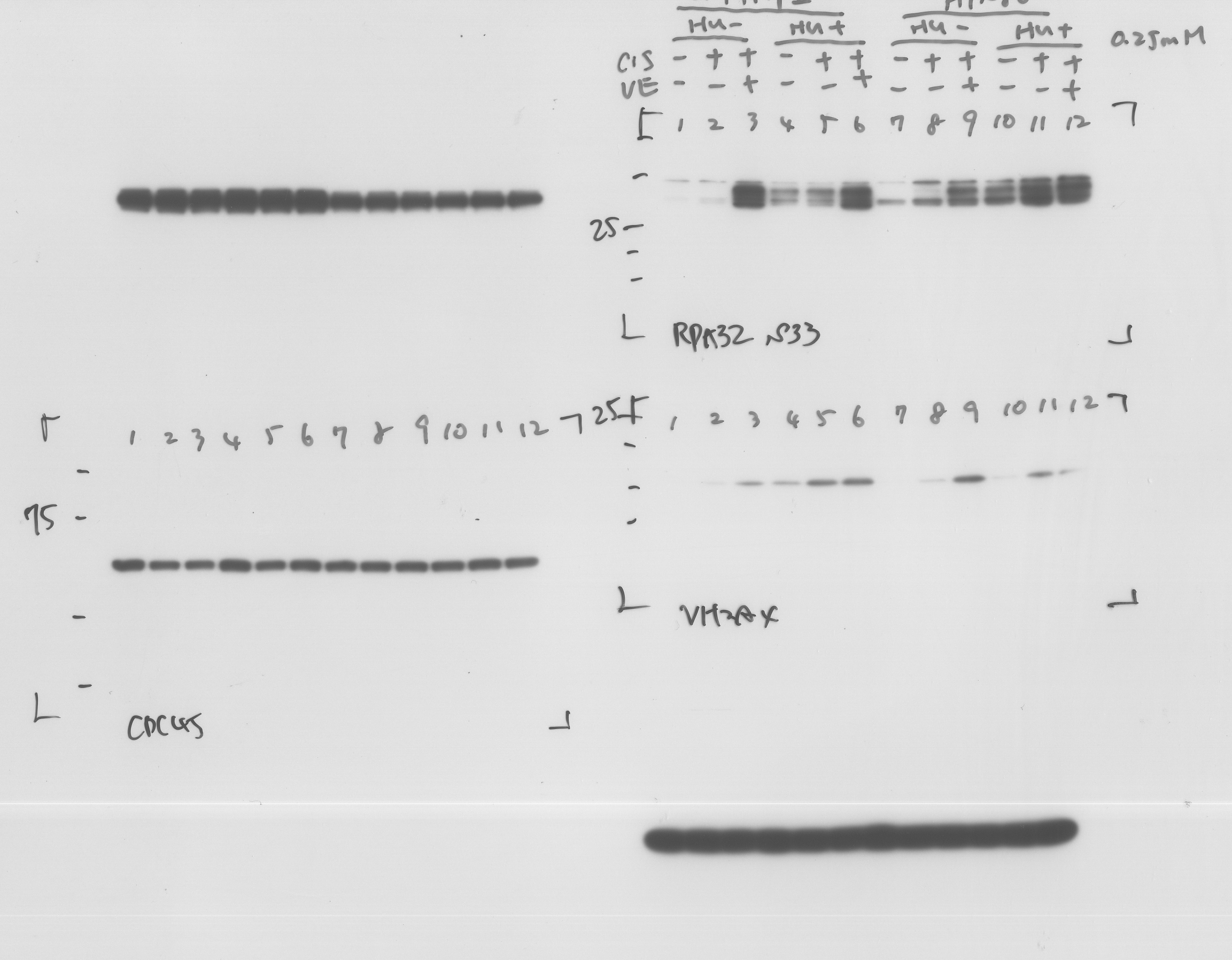

Supplement: Figure 7—source data 3. [file elife-83870-fig7-data3.zip › Figure 7-source data 3/Figure 7-source data 3 (CDC45_WCL).tif]

Figure 7-source data 3

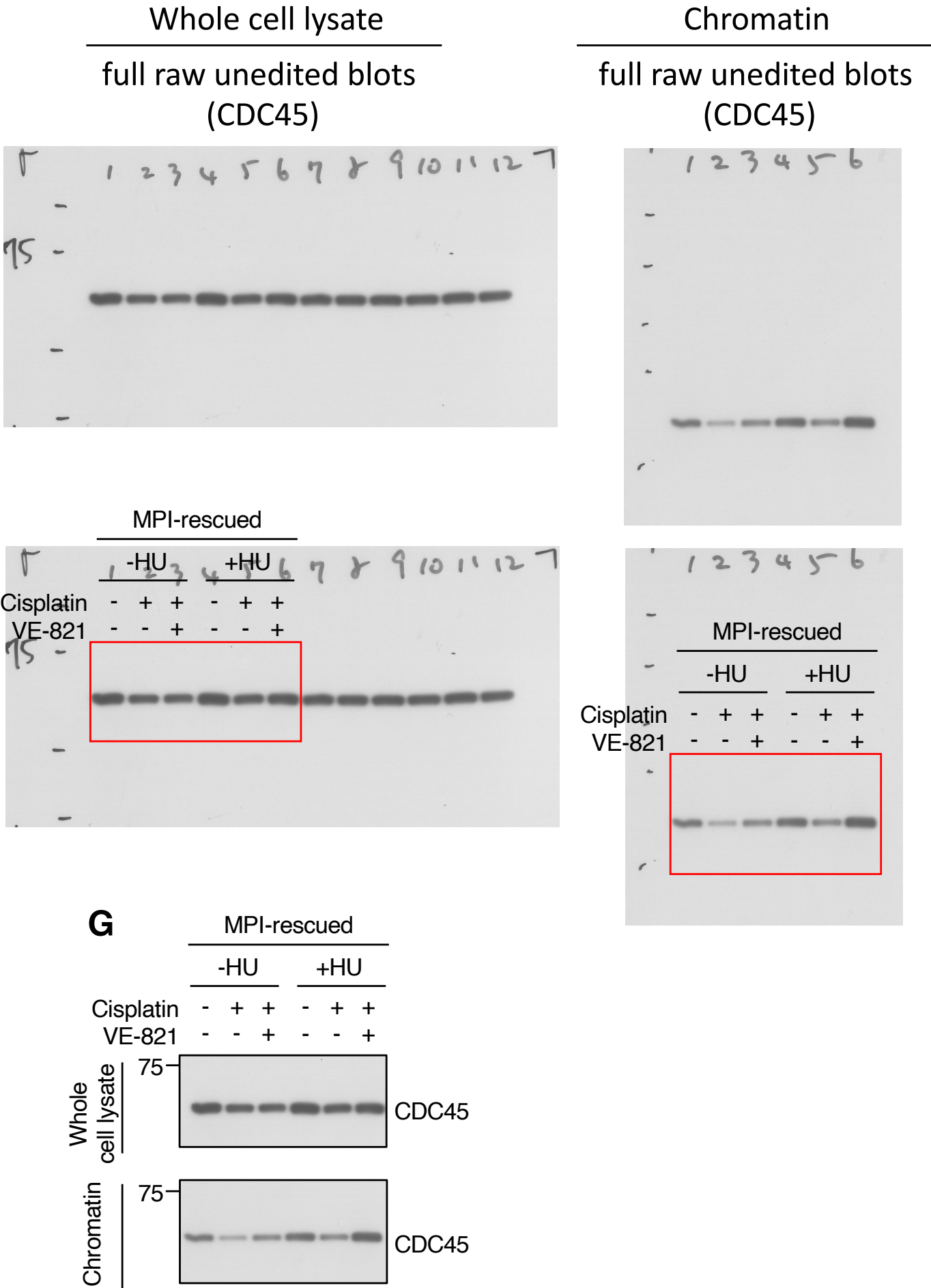

Supplement: Figure 7—source data 3. [file elife-83870-fig7-data3.zip › Figure 7-source data 3/Figure 7-source data 3.pdf]

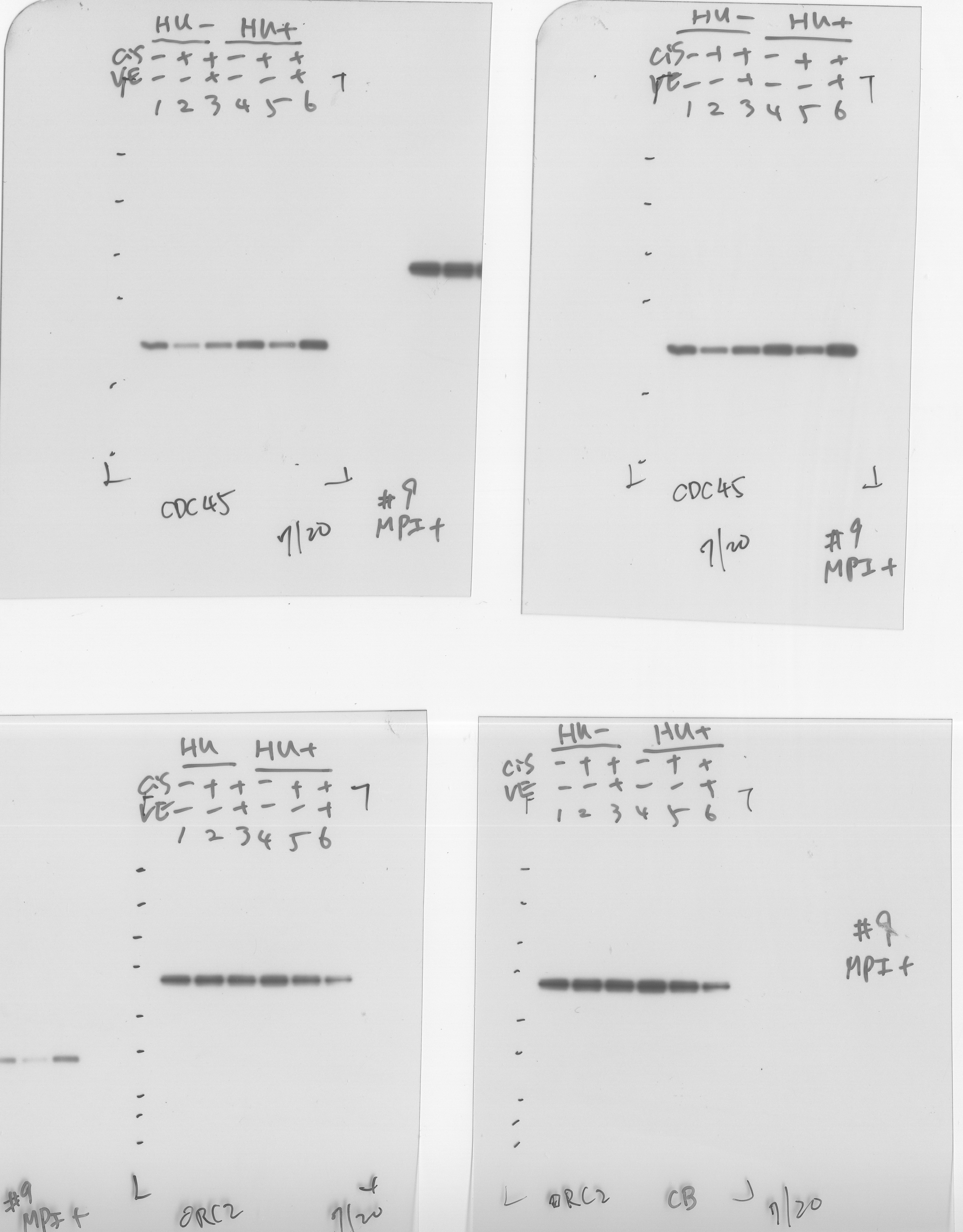

Supplement: Figure 7—source data 3. [file elife-83870-fig7-data3.zip › Figure 7-source data 3/Figure 7-source data 3 (CDC45_chromatin).tif]

Figure 7-source data 4

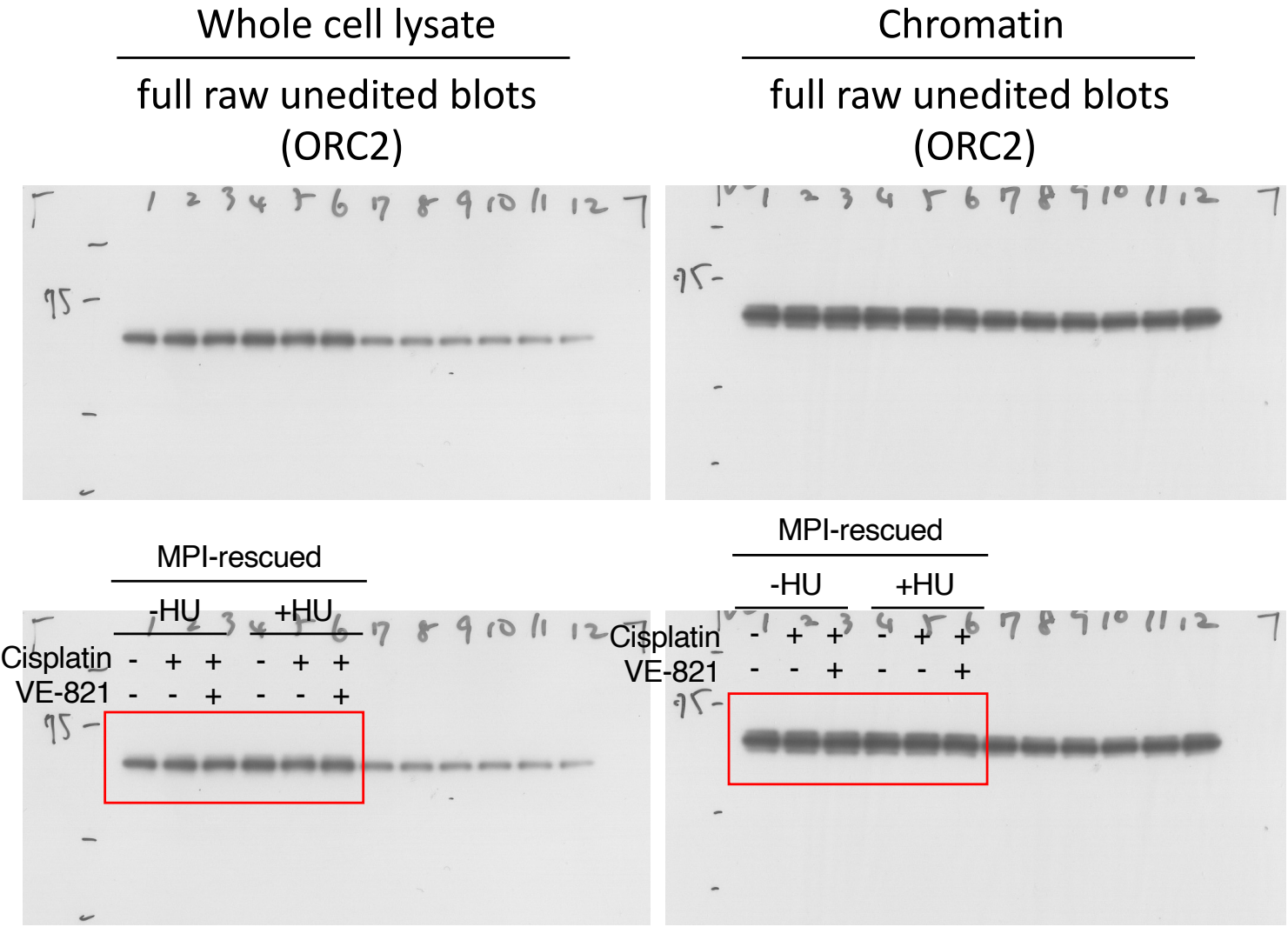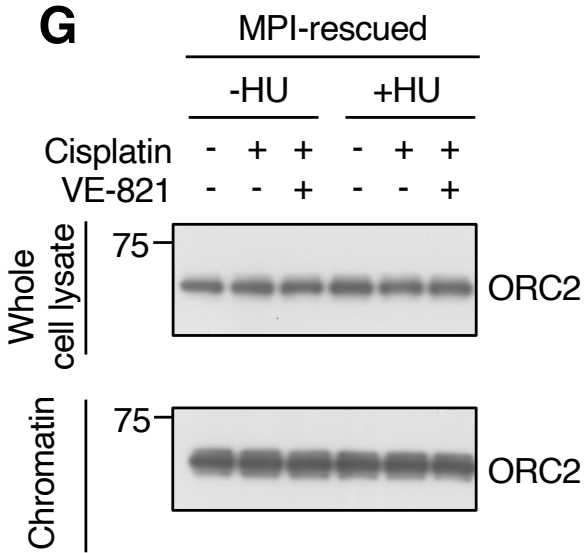

Supplement: Figure 7—source data 4. [file elife-83870-fig7-data4.zip › Figure 7-source data 4/Figure 7-source data 4.pdf]

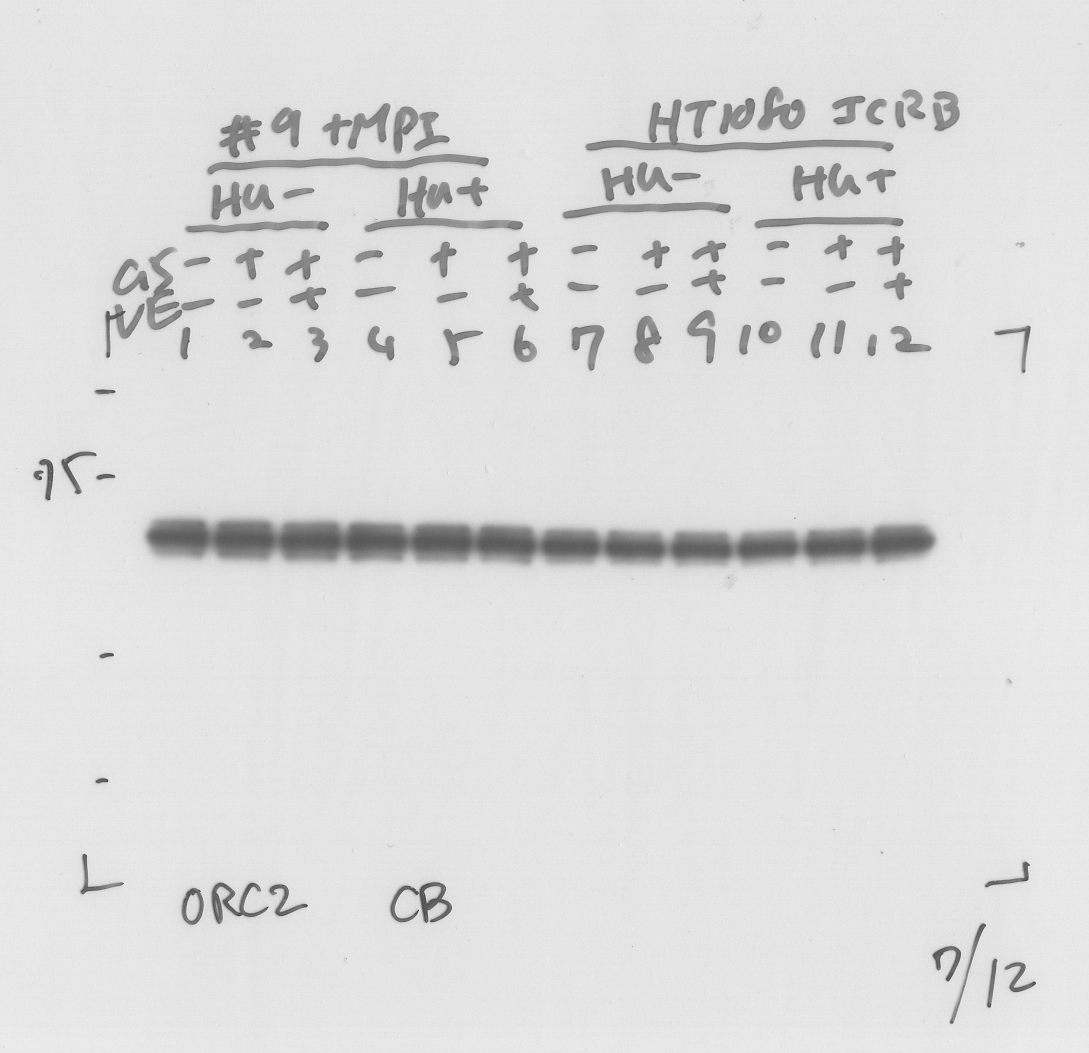

Supplement: Figure 7—source data 4. [file elife-83870-fig7-data4.zip › Figure 7-source data 4/Figure 7-source data 4 (ORC2_chromatin).tif]
