# Supplementary material for: Metabolic clogging of mannose triggers dNTP loss and genomic instability in human cancer cells
Source: eLife. 2023 Jul 18;12:e83870. doi: 10.7554/eLife.83870 (PMC10353863; doi:10.7554/eLife.83870)

Figure 1-figure supplement 1-source data 1

full raw unedited blots (β-Actin)

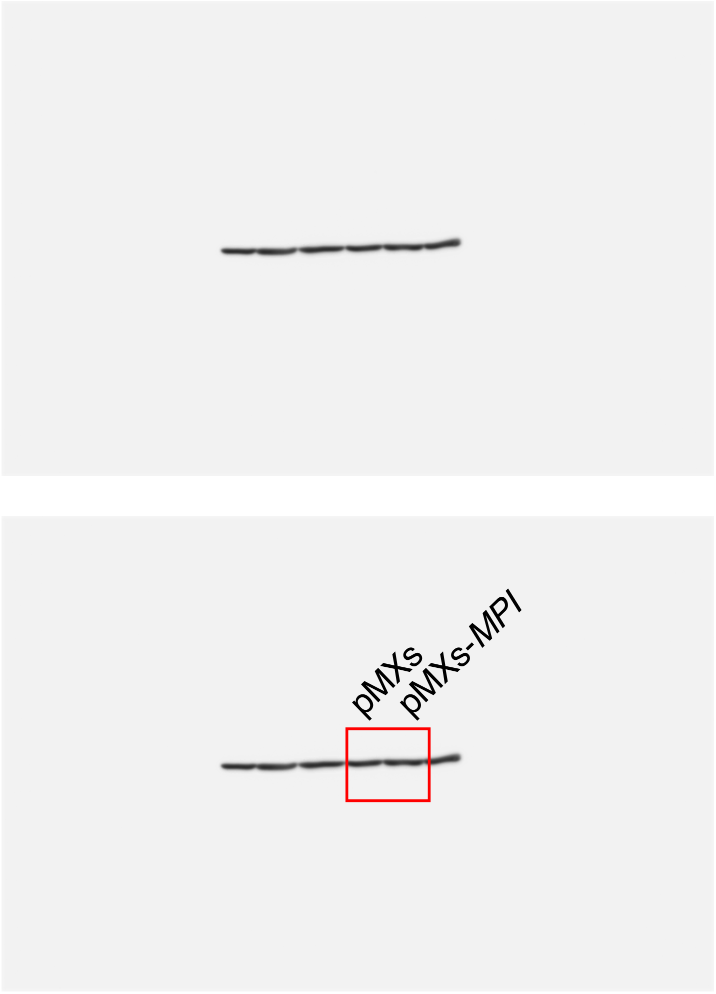

full raw unedited blots (MPI)

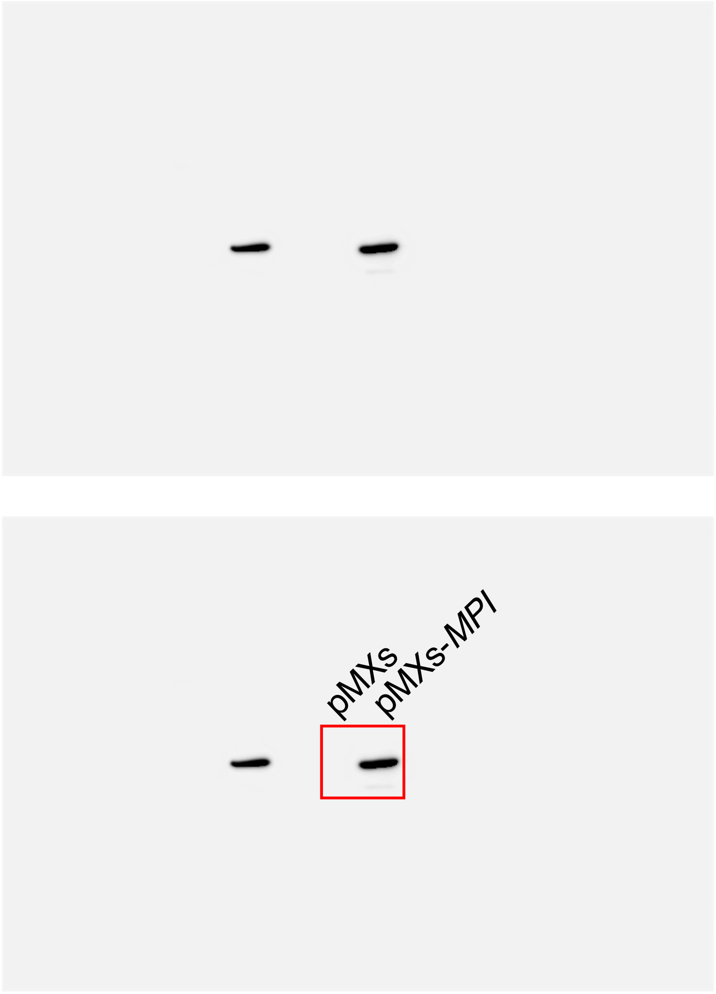

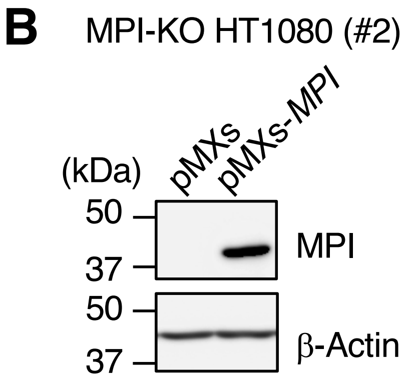

Supplement: Figure 1—figure supplement 2—source data 1. [file elife-83870-fig1-figsupp2-data1.zip › Figure 1-figure supplement 2-source data 1/Figure 1-figure supplement 1-source data 1.pdf]
