# Supplementary material for: Metabolic clogging of mannose triggers dNTP loss and genomic instability in human cancer cells
Source: eLife. 2023 Jul 18;12:e83870. doi: 10.7554/eLife.83870 (PMC10353863; doi:10.7554/eLife.83870)

Figure 3-source data 1

full raw unedited blots (β-Actin)	full raw unedited blots (MCM2)	full raw unedited blots (MCM3)

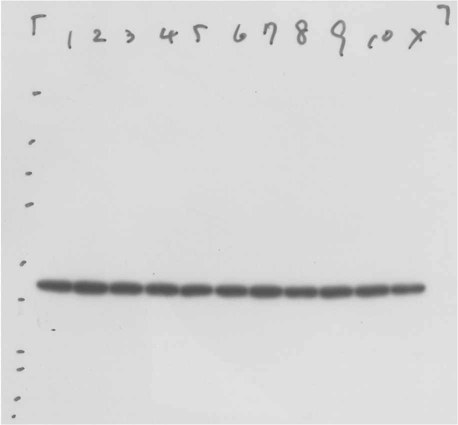

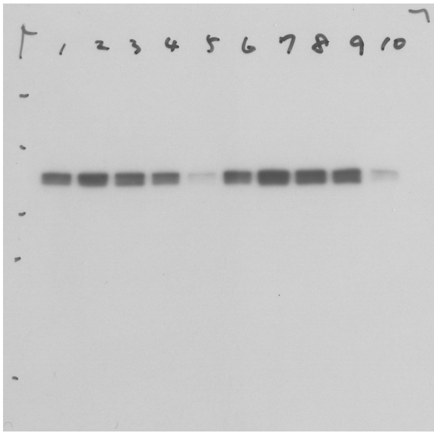

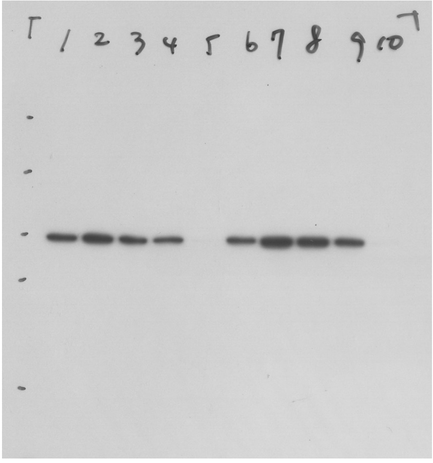

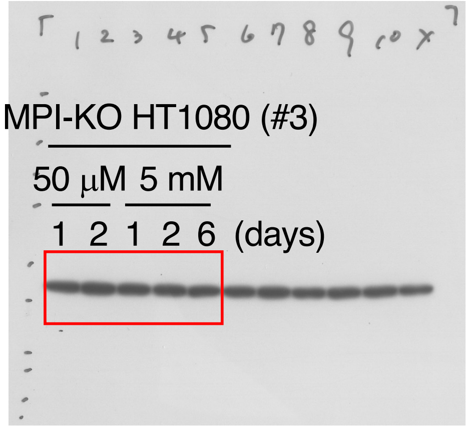

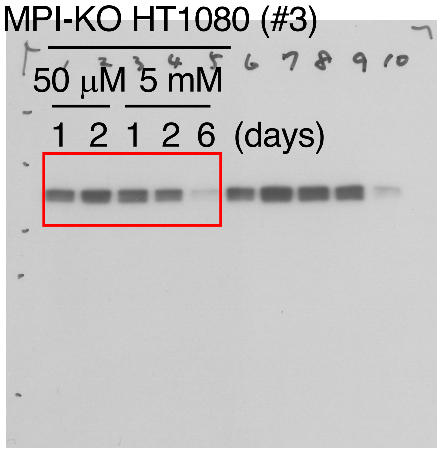

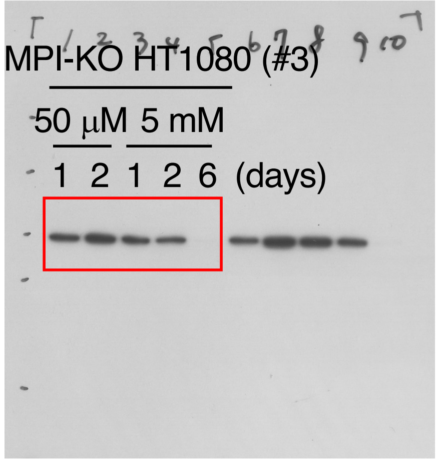

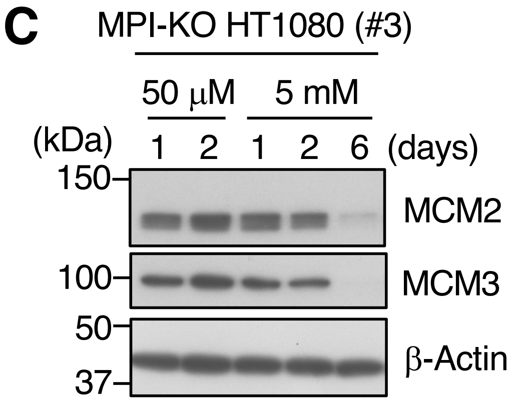

Supplement: Figure 3—source data 1. [file elife-83870-fig3-data1.zip › Figure 3-source data 1/Figure 3-source data 1 .pdf]
