# Supplementary material for: Metabolic clogging of mannose triggers dNTP loss and genomic instability in human cancer cells
Source: eLife. 2023 Jul 18;12:e83870. doi: 10.7554/eLife.83870 (PMC10353863; doi:10.7554/eLife.83870)

Figure 3-source data 2

full raw unedited blots (MCM4)

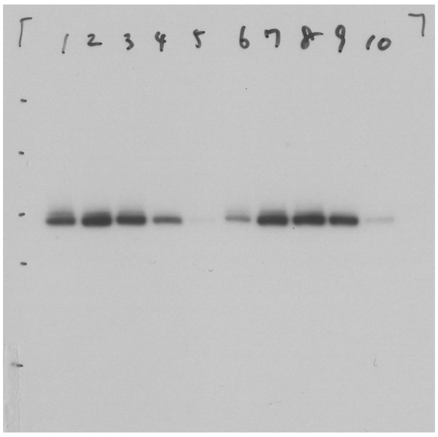

full raw unedited blots (MCM5)

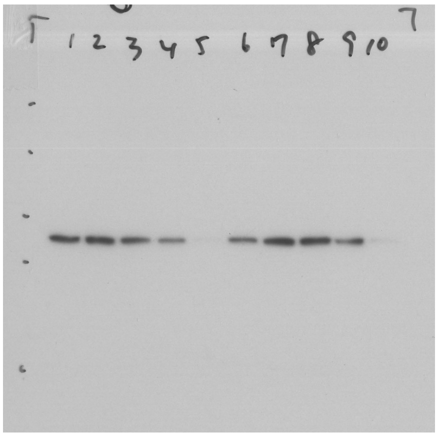

full raw unedited blots (MCM6)

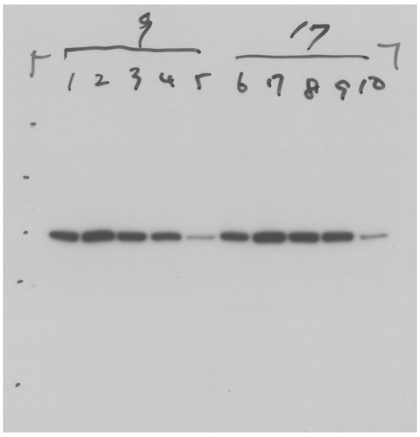

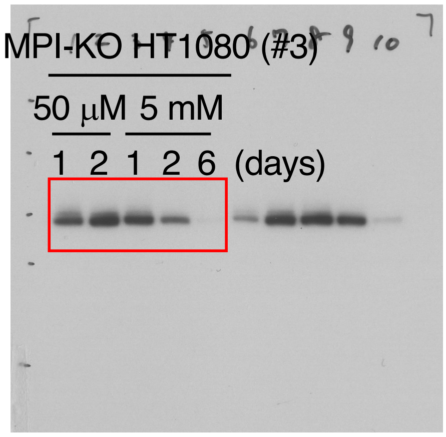

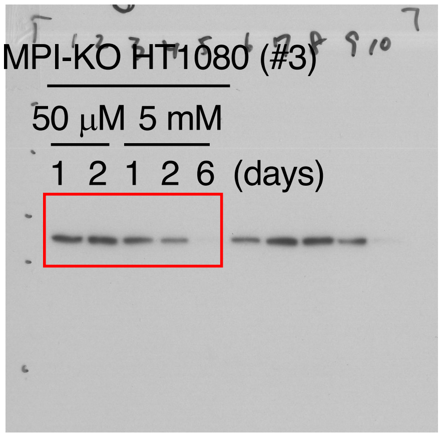

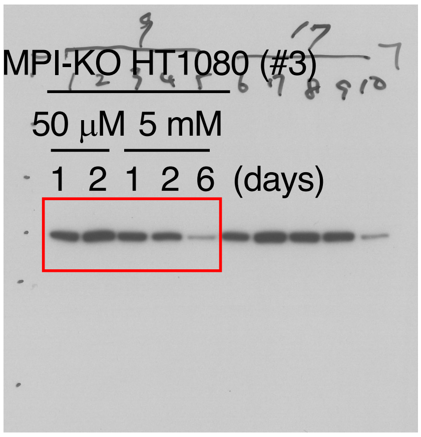

C

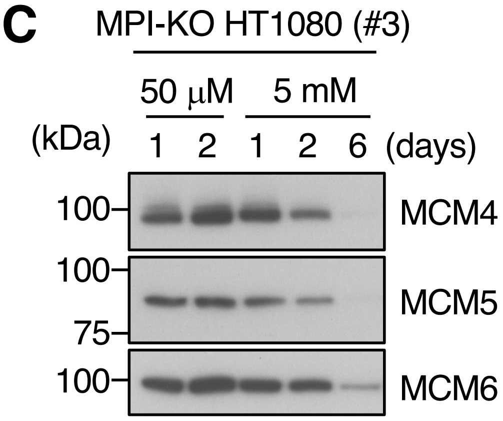

Supplement: Figure 3—source data 2. [file elife-83870-fig3-data2.zip › Figure 3-source data 2/Figure 3-source data 2 .pdf]
