# Supplementary material for: Metabolic clogging of mannose triggers dNTP loss and genomic instability in human cancer cells
Source: eLife. 2023 Jul 18;12:e83870. doi: 10.7554/eLife.83870 (PMC10353863; doi:10.7554/eLife.83870)

Figure 3-source data 3

full raw unedited blots (MCM7) | full raw unedited blots (CDC6) | full raw unedited blots (CDT1)

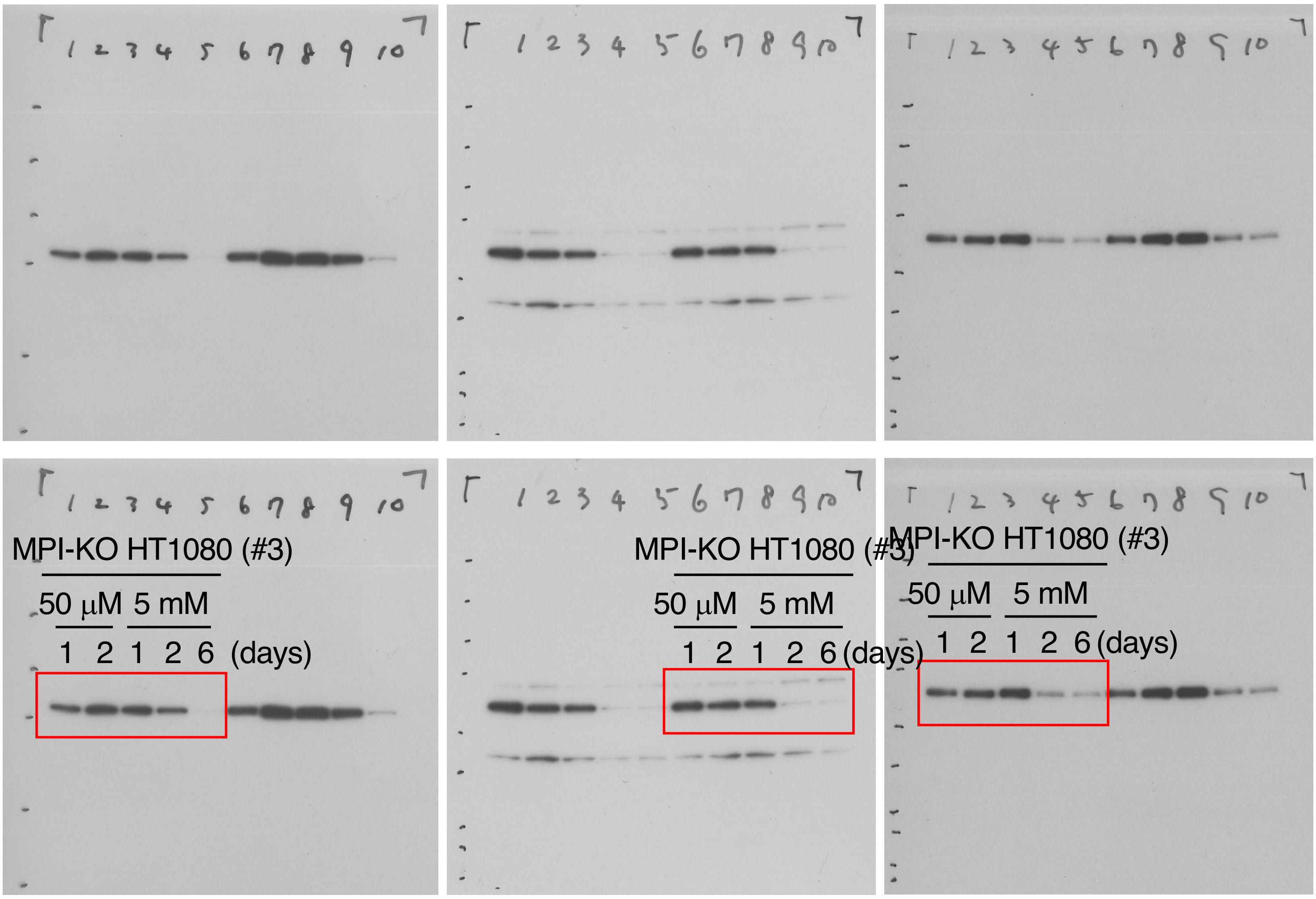

C

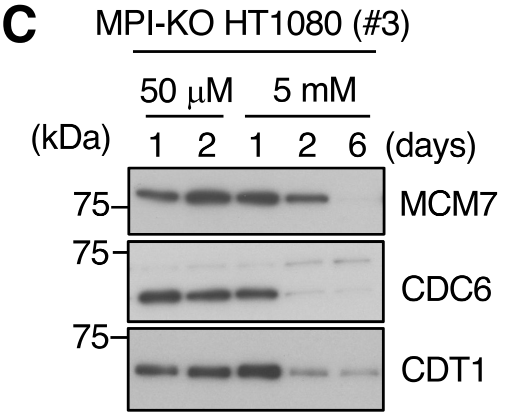

Supplement: Figure 3—source data 3. [file elife-83870-fig3-data3.zip › Figure 3-source data 3/Figure 3-source data 3.pdf]
