# Supplementary material for: Metabolic clogging of mannose triggers dNTP loss and genomic instability in human cancer cells
Source: eLife. 2023 Jul 18;12:e83870. doi: 10.7554/eLife.83870 (PMC10353863; doi:10.7554/eLife.83870)
Supplement: Supplementary file 1. [file elife-83870-supp1.docx]

**Supplementary File 1.**

List of primers used in this study.

| Primer # | Primer name | Sequences (5’-3’) |
| --- | --- | --- |
| 1 | hMPI-WT-Fw | GCACTGAGTATCCCCCTAAG |
| 2 | hMPI-KO-Fw | CTGTGGGGAGCACAGCATAA |
| 3 | hMPI-WT/KO-Rv | GCCTGCGTTCAAATTGTGGT |
| 4 | CACC_hMPI-Fw | CACCATGGCCGCTCCGCGAG |
| 5 | hMPI_w/oSTOP-Rv | CAGCAGACAGCAGGCAC |
| 6 | Infusion_hMPI-Fw | AGTTAATTAAGGATCGCCACCATGGCCGCTCCG |
| 7 | Infusion_hMPI-Rv | ACTGTGCTGGCGGCCTTACAGCAGACAGCAGGC |
| 8 | Infusion_mC-hCdt1-Fw | AGTTAATTAAGGATCGCCACCATGGTGAGCAAG |
| 9 | Infusion_mC-hCdt1-Rv | ACTGTGCTGGCGGCCTTATTTCTTTATCTTCTGGCC |
| 10 | qPCR_hACTB-Fw | CACCATTGGCAATGAGCGGTTC |
| 11 | qPCR_hACTB-Rv | AGGTCTTTGCGGATGTCCACGT |
| 12 | qPCR_hCDC6-Fw | GGAGATGTTCGCAAAGCACTGG |
| 13 | qPCR_hCDC6-Rv | GGAATCAGAGGCTCAGAAGGTG |
| 14 | qPCR_hCDT1-Fw | GGAGGTCAGATTACCAGCTCAC |
| 15 | qPCR_hCDT1-Rv | TTGACGTGCTCCACCAGCTTCT |
| 16 | qPCR_hMCM2-Fw | TGCCAGCATTGCTCCTTCCATC |
| 17 | qPCR_hMCM2-Rv | AAACTGCGACTTCGCTGTGCCA |
| 18 | qPCR_hMCM3-Fw | CGAGACCTAGAAAATGGCAGCC |
| 19 | qPCR_hMCM3-Rv | GCAGTGCAAAGCACATACCGCA |
| 20 | qPCR_hMCM4-Fw | CTTGCTTCAGCCTTGGCTCCAA |
| 21 | qPCR_hMCM4-Rv | GTCGCCACACAGCAAGATGTTG |
| 22 | qPCR_hMCM5-Fw | GACTTACTCGCCGAGGAGACAT |
| 23 | qPCR_MCM5-Rv | TGCTGCCTTTCCCAGACGTGTA |
| 24 | qPCR_hMCM6-Fw | GACAACAGGAGAAGGGACCTCT |
| 25 | qPCR_hMCM6-Rv | GGACGCTTTACCACTGGTGTAG |
| 26 | qPCR_hMCM7-Fw | GCCAAGTCTCAGCTCCTGTCAT |
| 27 | qPCR_hMCM7-Rv | CCTCTAAGGTCAGTTCTCCACTC |
