# Supplementary material for: Metabolic clogging of mannose triggers dNTP loss and genomic instability in human cancer cells
Source: eLife. 2023 Jul 18;12:e83870. doi: 10.7554/eLife.83870 (PMC10353863; doi:10.7554/eLife.83870)
Supplement: Supplementary file 2. [file elife-83870-supp2.docx]

Supplementary File 2.

List of plasmids used in this study.

| Plasmid # | Plasmid name | Notes |
| --- | --- | --- |
| 1 | pENTR-hMPI | This study |
| 2 | pMXs-Neo-hMPI | This study |
| 3 | mCherry-hCdt1(1/100)Cy(-)/pcDNA3 | (Sakaue-Sawano *et al*., 2017) |
| 4 | pMXs-Neo-mCherry-hCdt1(1/100)Cy(-) | This study |
| 5 | pMMLV-mVenus-hGem(1/110):IRES:Bsd | This study |
